# *C. elegans* somatostatin/allatostatin C signaling regulates sleep, metabolism, survival, and memory via a sleep-active neuron

**DOI:** 10.1126/sciadv.adv8387

**Published:** 2026-04-15

**Authors:** Byoungjun Park, Lama Mohsen, Inka Busack, Laura Uhlig, Lorenzo Rossi, Gill Pollmeier, Ellen Geens, Majdulin Nabil Istiban, Sajal Mandal, Reshma Dominic Savio, Isabel Beets, Attila Stetak, Henrik Bringmann

**Affiliations:** ^1^ Biotechnology Center and Medical Faculty, Technische Universität Dresden, Am Tatzberg 47/49, 01307 Dresden, Germany.; ^2^ Department of Biology, KU Leuven, Naamsestraat 59, 3000 Leuven, Belgium.; ^3^ Division Cluster Molecular and Cognitive Neurosciences, Universität Basel, Birmannsgasse 8, 4055 Basel, Switzerland.; ^4^ Faculty of Medicine Carl Gustav Carus, Technische Universität Dresden, Fetscherstraße 74, 01307 Dresden, Germany.

## Abstract

Somatostatin/allatostatin C signaling regulates sleep, metabolism, memory, and longevity, but the underlying neuronal mechanisms remain unclear. We investigated the *Caenorhabditis elegans* somatostatin/allatostatin C ortholog NLP-99 and its receptor NPR-16. We found that the wake-active AIY neurons release NLP-99 to activate NPR-16. NPR-16 is G_i/o_ coupled and acts cell nonautonomously to activate the sleep-active RIS neuron while acting autonomously in RIS to inhibit calcium activation and the release of the sleep-inducing FLP-11 neuropeptides. During larval arrest, strong NPR-16 expression in RIS causes NLP-99/NPR-16 signaling to inhibit sleep, reducing lipid storage and survival. In well-fed adults, NPR-16 expression is stronger outside of RIS, and NLP-99/NPR-16 signaling is required for RIS activation and sleep while also inhibiting longevity via RIS. Both NLP-99/NPR-16 and RIS/FLP-11 are required for memory consolidation. These results show that NLP-99/NPR-16 regulates physiological processes via control of RIS. A similar somatostatin-mediated regulation of sleep neurons may underlie physiological regulation in other species.

## Teaser

A conserved somatostatin pathway regulates sleep and physiology through a sleep-active neuron.

## INTRODUCTION

Somatostatin was first identified more than 50 years ago as a hypothalamic hormone that inhibits the release of growth hormone (GH) from the pituitary gland in mammals (*1*). It controls the pulsatile release of GH and, while not playing a major role in developmental growth, helps limit obesity at older ages (*2*). Somatostatin inhibition of GH and other anabolic hormones may influence lifespan, but the role of somatostatin in longevity and aging remains poorly understood (*3*).

GH is released during sleep, where it is thought to promote sleep and support anabolic processes (*4*). Sleep is crucial for various physiological functions, including energy conservation and allocation (*5*), the regulation of metabolism and gene expression (*6*), long-term memory (LTM) consolidation (*7*), and longevity and survival (*8*, *9*). Thus, both somatostatin and sleep regulate overlapping essential physiological processes. However, the molecular and neuronal mechanisms through which somatostatin signaling and sleep coordinate these diverse physiological processes are not well understood.

Sleep is induced by sleep-active neurons that release inhibitory neurotransmitters, such as γ-aminobutyric acid (GABA) and neuropeptides, to inhibit wake-promoting circuits (*10*). As wake-promoting circuits in turn inhibit sleep-promoting circuits, distinct states of sleep and wakefulness can be generated, functioning like a flip-flop switch (*11*). Somatostatin- and GABA-expressing neurons, for example, in the basal forebrain and ventral tegmental area of the midbrain, have been shown to play sleep-promoting roles (*12*, *13*). In the cortex, somatostatin- and GABA-expressing neurons are active during non–rapid eye movement (NREM) sleep, with their activity increasing following sleep deprivation. During NREM sleep, cortical neurons oscillate synchronously between up and down states, a process known as slow oscillation. Up states are characterized by membrane depolarization and wake-like tonic firing, while down states are marked by membrane hyperpolarization and neuronal silence (*14*). Somatostatin-expressing cortical neurons activate immediately before down states. Optogenetic stimulation of these neurons induces prolonged down states, and chemogenetic activation enhances slow oscillation (*14*, *15*). In addition, activation of cortical somatostatin- and GABA-expressing neurons triggers sleep-preparatory behavior, followed by intense recovery sleep (*16*). Although some somatostatin- and GABA-expressing neurons are known to promote sleep, the role of somatostatin in these neurons in sleep promotion is not yet understood. Somatostatin analogs suppress NREM sleep in rodents and elderly humans (*17*, *18*). In addition, a presumptive somatostatin hypomorphic mutant increases NREM sleep (*19*). These findings suggest that somatostatin may overall exert an inhibitory role in sleep. While previous studies have mostly focused on the sleep-promoting role of somatostatin- and GABA-expressing neurons, the sleep-inhibitory role of somatostatin remains largely unclear.

Somatostatin appears to exert both memory-supporting and memory-inhibiting roles. On the one hand, overactivation of somatostatin neurons in the hippocampus is thought to impair memory consolidation, suggesting an inhibitory role for these neurons in memory (*20*). Sleep deprivation increases the activity of somatostatin-expressing neurons, potentially leading to impaired memory (*15*). On the other hand, somatostatin- and GABA-expressing neurons can encode memory (*21*, *22*), and deleting the somatostatin gene impairs memory consolidation (*23*). Thus, both somatostatin and sleep are crucial for memory consolidation (*7*), yet the connection between the memory-promoting role of somatostatin and sleep remains unclear.

In mice, somatostatin activates five heterotrimeric guanine nucleotide–binding protein (G protein)–coupled receptors (GPCRs). In vitro studies show that these receptors reduce intracellular Ca^2+^ and adenosine 3′,5′-monophosphate (cAMP). However, it is not well understood how these receptors regulate anabolism, survival/longevity, sleep, and memory, partly due to the potential redundancy among them (*24*).

In protostomes, such as insects, allatostatin C (AstC) is the ortholog of mammalian somatostatin. It was identified as a hormone that inhibits juvenile hormone, which promotes growth, metamorphosis, and reproduction (*25*). In *Drosophila*, the diet has been shown to control sleep quality by peptidergic signaling from the gut to the brain (*26*). Endocrine cells in the gut detect energy scarcity and increase the release of AstC when nutrients are depleted. AstC acts through AstC receptor 2 (AstC-R2), which is orthologous to mammalian somatostatin receptors. AstC-R2 functions in neurosecretory tissues similar to pancreatic α cells to mobilize stored lipids and carbohydrates, promote food intake, and suppress sleep, potentially allowing more time for foraging (*27*). Thus, like vertebrate somatostatins, AstC inhibits anabolic functions by suppressing growth- and reproduction-promoting hormones. However, its mechanism in regulating sleep and survival is not yet understood.

In *Caenorhabditis elegans*, recent bioinformatic analyses and in vitro receptor deorphanization screening have identified the neuropeptide NLP-99 as an ortholog of somatostatin/AstC peptides and the neuropeptide receptor NPR-16 as an ortholog of vertebrate and insect somatostatin/AstC receptors (*28*–*30*). NLP-99 specifically activates NPR-16 at nanomolar concentrations, and NPR-16 is known to be activated solely by NLP-99 (*28*). However, the functions of this highly specific and conserved ligand-receptor pair remain unknown.

*C. elegans* sleep crucially requires a single sleep-active neuron called RIS. Depolarization of RIS induces sleep by inhibiting wakefulness circuits through the release of RFamide neuropeptides encoded by the *flp-11* gene (*31*, *32*). FLP-11 neuropeptides released from RIS induce sleep by activating the inhibitory GPCR DMSR-1 in cholinergic neurons (*33*). Sleep in *C. elegans* is increased upon stressful events that include molting (*34*), cellular stress (*35*, *36*), wounding (*9*), and starvation (*8*, *37*). If larvae hatch without food, they arrest development and alternate between sleep and wakefulness phases, a process regulated by a conserved aging gene network (*8*). Activation of the RIS neuron and subsequent FLP-11 release promote protective gene expression changes, suppress aging phenotypes, and aid survival during L1 arrest (*38*, *39*). RIS is thought to be regulated through upstream neuronal circuits that regulate its calcium activity. For example, homeostatic regulation of RIS activity involves rebound calcium activation following prior inhibition (*40*). Likewise, RIS activation leads to subsequent self-inhibition via released FLP-11 (*40*), which activates DMSR-1 in RIS (*33*). Mechanisms regulating RIS activation beyond the calcium concentration have not yet been identified.

Here, we studied NLP-99 and NPR-16 to explore how sleep, survival/longevity, anabolism, and memory are interconnected through somatostatin/AstC signaling. Our findings indicate that *C. elegans* somatostatin/AstC signaling coordinates essential physiological processes by controlling RIS activity, including its calcium dynamics and neurotransmission.

## RESULTS

### NLP-99 and NPR-16 inhibit sleep during starvation yet promote RIS calcium activation

**Fig. 1.**
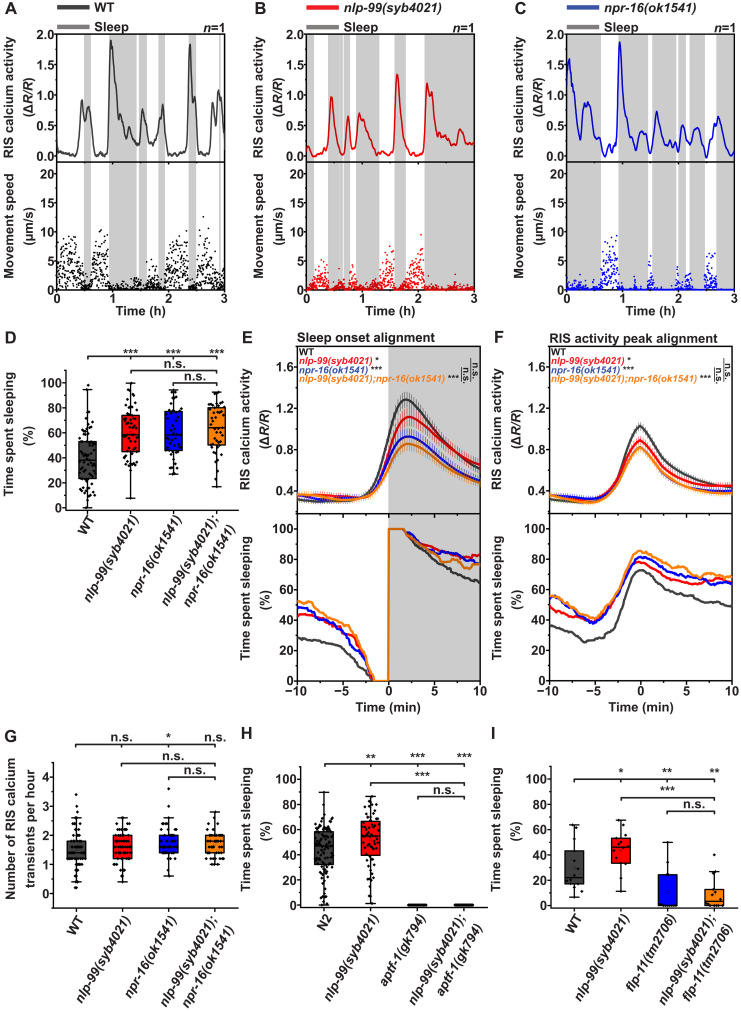
NLP-99 and NPR-16 inhibit sleep during L1 arrest. (**A** to **C**) Example data of RIS calcium activity, movement speed, and sleep detection. Sleep phases are labeled with a gray shade. WT, wild type; h, hours. (**D** to **G**) Wild type (*n* = 73, five replicates), *nlp-99(syb4021)* (*n* = 59, five replicates), *npr-16(ok1541)* (*n* = 47, three replicates), and *nlp-99(syb4021);npr-16(ok1541)* (*n* = 45, three replicates). (D) Time spent sleeping. Wild type (mean = 39.9%), *nlp-99(syb4021)* (mean = 59.9%), *npr-16(ok1541)* (mean = 60.2%), and *nlp-99(syb4021);npr-16(ok1541)* (mean = 63.6%). (E) Sleep bout onset alignment and RIS calcium activity peak size. Wild type [mean = 1.34 a.u. (arbitrary units)], *nlp-99(syb4021)* (mean = 1.16 a.u.), *npr-16(ok1541)* (mean = 0.98 a.u.), and *nlp-99(syb4021);npr-16(ok1541)* (mean = 0.90 a.u.). (F) RIS calcium activation peak alignment and RIS calcium activity peak size. Wild type (mean = 1.02 a.u.), *nlp-99(syb4021)* (mean = 0.88 a.u.), *npr-16(ok1541)* (mean = 0.82 a.u.), and *nlp-99(syb4021);npr-16(ok1541)* (mean = 0.82 a.u.). (G) RIS calcium transient frequency. Wild type (mean = 1.5 bouts per hour), *nlp-99(syb4021)* (mean = 1.6 bouts per hour), *npr-16(ok1541)* (mean = 1.8 bouts per hour), and *nlp-99(syb4021);npr-16(ok1541)* (mean = 17.0 bouts per hour). (**H**) Sleep analysis in *aptf-1(gk794);nlp-99(syb4021)*. Wild type (mean = 43.80%, *n* = 110, six replicates, same data as in fig. S1A), *nlp-99(syb4021)* (mean = 51.81%, *n* = 64, four replicates), *aptf-1(gk794)* (mean = 0%, *n* = 24, two replicates), and *nlp-99(syb4021);aptf-1(gk794)* (mean = 0%, *n* = 31, two replicates). (**I**) Sleep analysis in *nlp-99(syb4021)*;*flp-11(tm2706)*. Wild type (mean = 39.7%, *n* = 14, four replicates), *nlp-99(syb4021)* (mean = 43.7%, *n* = 14, four replicates), *flp-11(tm2706)* (mean = 10.4%, *n* = 13, four replicates), and *nlp-99(syb4021);flp-11(tm2706)* (mean = 9.5%, *n* = 14, four replicate). Mann-Whitney *U* test. n.s., not significant; *P < 0.05, **P < 0.01, and ***P < 0.001.

We aimed to understand the role of the predicted somatostatin/AstC peptide and somatostatin/AstC receptor orthologs, NLP-99 and NPR-16, in sleep regulation. To achieve this goal, we quantified sleep and RIS calcium activity during L1 arrest in *nlp-99(syb4021)* and *npr-16(ok1541)* deletion mutants. We cultured arrested L1 larvae in agarose hydrogel microfluidic chambers and measured sleep using immobility and RIS calcium activity using GCaMP fluorescence (Fig. 1, A to C) (*8*, *40*–*42*). Both *nlp-99(syb4021)* and *npr-16(ok1541)* mutants increased the fraction of time spent sleeping by ~50%, and double mutation did not further increase sleep, supporting the idea that both genes act within the same pathway to inhibit sleep (Fig. 1D). We also confirmed the sleep-inhibitory role of *nlp-99* using a second allele, *nlp-99(ibt13)* (fig. S1A). To determine whether the mutants affected general locomotion activity, we quantified movement speed during wakefulness. *nlp-99(syb4021)*, *npr-16(ok1541)*, and wild-type animals displayed a similar average motion speed during wakefulness, indicating that *nlp-99* does not broadly regulate movement (fig. S1B). Similar to starved larvae, deletion of *nlp-99* and *npr-16* also increased sleep in starved adults (fig. S1C) (*8*, *37*).

We next analyzed the relationship between RIS calcium activity and sleep in L1 arrest. To analyze RIS activation during sleep induction, we aligned sleep bouts to their onset and quantified the corresponding activity of the RIS cell body (*8*, *39*). In L1 arrest, the average calcium activation of RIS during sleep was reduced in both mutants, with a more pronounced reduction in the *npr-16* mutant (Fig. 1E). To examine the sleep response to RIS activation, we extracted and aligned all RIS calcium transients to their peaks and quantified the corresponding fraction of time spent sleeping (Fig. 1F). This analysis showed that the frequency of RIS calcium transients was not altered in either mutant (Fig. 1G). Despite being associated with increased sleep, the magnitude of RIS calcium transients was reduced in both mutants (Fig. 1F). In addition to the cell body analysis, we extracted the calcium signal from the nerve ring (*43*) but found no significant differences between the wild type and the single mutants. However, the double mutant exhibited a significant reduction in calcium activity, consistent with the cell body measurements (fig. S1D). These results suggest that NLP-99 and NPR-16 play a complex regulatory role in RIS: They are required for RIS calcium activation but limit its ability to induce sleep.

To test whether the increased sleep of *nlp-99* and *npr-16* mutants during L1 arrest depends on RIS, we impaired RIS function using *aptf-1* and *flp-11* mutations. Deletion of the *aptf-1* gene prevents RIS from expressing *flp-11*, the gene encoding key neuropeptides for sleep induction by RIS (*31*, *32*, *39*). *flp-11* deletion mutants exhibit a milder sleep loss phenotype compared to *aptf-1* deletion mutants (*32*, *39*), indicating that FLP-11 is the primary, although not the only, transmitter used by RIS to induce sleep. In the *nlp-99* mutant, sleep was entirely dependent on *aptf-1* (Fig. 1H) and mostly dependent on *flp-11* (Fig. 1I). Similarly, most of the increased sleep caused by *npr-16* deletion was dependent on *flp-11* (fig. S1E). Our results therefore suggest that *nlp-99* and *npr-16* regulate a sleep pathway that is entirely dependent on RIS and partially dependent on FLP-11.

### NLP-99 and NPR-16 are required for RIS calcium activation and sleep in fed adults

**Fig. 2.**
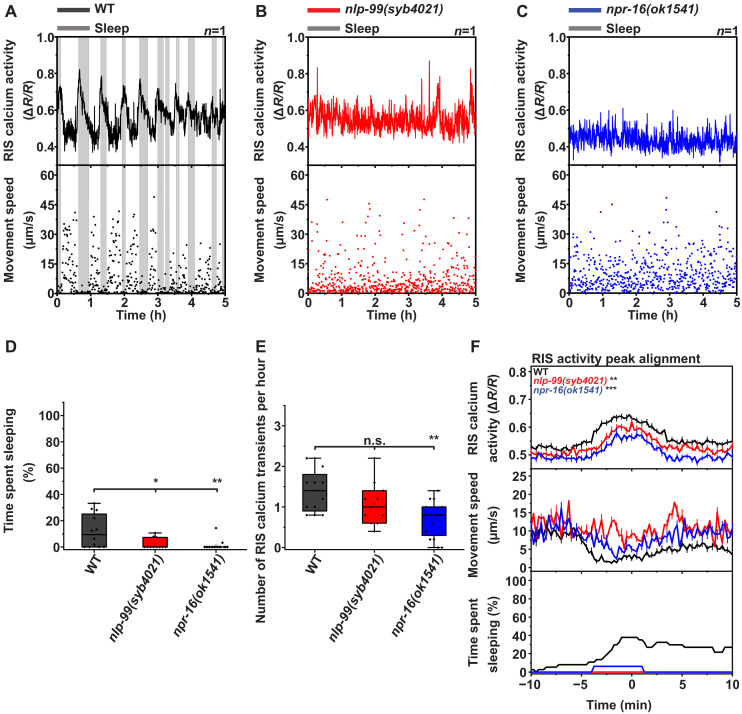
NLP-99 and NPR-16 are required for RIS calcium activity and sleep induction downstream of RIS calcium activation in well-fed adults. (**A** to **C**) Example data of RIS calcium activity, movement speed, and sleep detection. Sleep phases are labeled with a gray shade. (**D** to **F**) Wild type (*n* = 12, four replicates), *nlp-99(syb4021)* (*n* = 11, four replicates), and *npr-16(ok1541)* (*n* = 16, four replicates). (D) Time spent sleeping. Wild type (mean = 12.2%), *nlp-99(syb4021)* (mean = 2.3%), and *npr-16(ok1541)* (mean = 1.1%). (E) RIS calcium transient frequency. Wild type (mean = 1.4 bouts per hour), *nlp-99(syb4021)* (mean = 1.1 bouts per hour), and *npr-16(ok1541)* (mean = 0.7 bouts per hour). (F) RIS calcium activation peak alignment and RIS calcium activity peak size. Wild type (mean = 0.63 a.u.), *nlp-99(syb4021)* (mean = 0.60 a.u.), and *npr-16(ok1541)* (mean = 0.57 a.u.). Mann-Whitney *U* test. *P < 0.05, **P < 0.01, and ***P < 0.001.

To test the effects of *nlp-99* and *npr-16* deletion on fed adults, we measured sleep in deletion mutants in nematode growth media (NGM)–based microchambers seeded with bacteria (Fig. 2, A to C) (*8*). In well-fed adults, deletion of either *nlp-99* or *npr-16* reduced sleep, with *npr-16* deletion producing the stronger effect (Fig. 2D).

Because *nlp-99* and *npr-16* mutants showed almost no detectable sleep, sleep bout alignment was not possible. We therefore focused our analysis on RIS calcium activity. The frequency of RIS calcium transients was reduced in both mutants, but this reduction was stronger and only statistically significant for *npr-16* deletion (Fig. 2E). Peak alignment revealed that RIS calcium activity was generally reduced, with weaker peaks that were associated with reduced sleep (Fig. 2F). This suggests that in *nlp-99* and *npr-16* mutants, RIS activation is overall reduced and ineffective at inducing sleep.

### NLP-99 and NPR-16 inhibit FLP-11 release from RIS

**Fig. 3.**
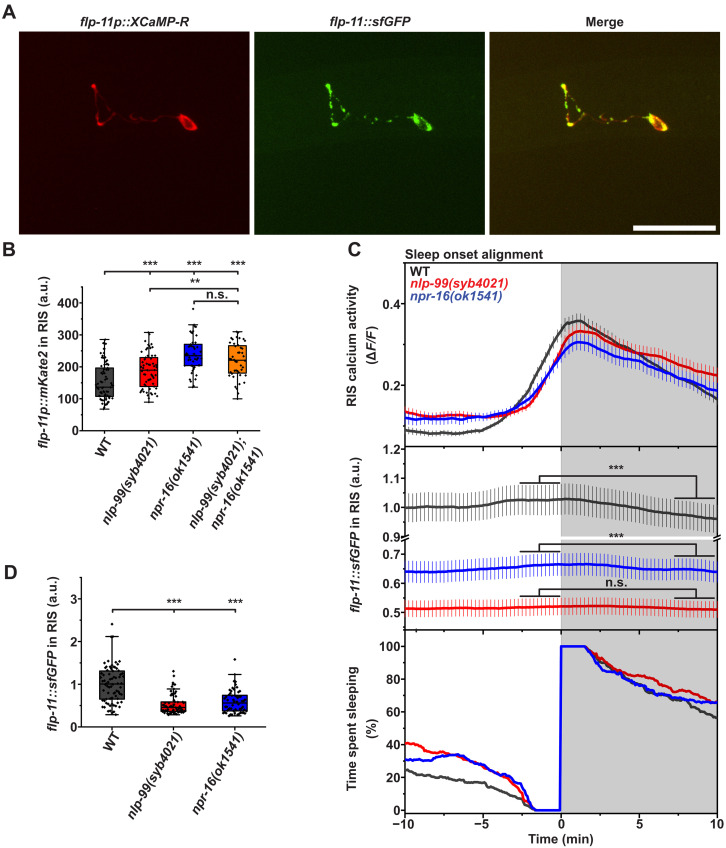
NLP-99 and NPR-16 inhibit FLP-11 release from RIS. (**A**) The translational (*flp-11::sfGFP*) reporter of *flp-11* along with XCaMP-R allows for quantification of FLP-11 secretion from RIS during its calcium activation. The scale bar represents 25 μm. (**B**) Deletion of *nlp-99* and *npr-16* increases the transcriptional activity of *flp-11* in RIS during L1 arrest. Wild type (mean = 153 a.u., *n* = 73, five replicates), *nlp-99(syb4021)* (mean = 187 a.u., *n* = 59, five replicates), *npr-16(ok1541)* (mean = 238 a.u., *n* = 47, three replicates), and *nlp-99(syb4021);npr-16(ok1541)* (mean = 218 a.u., *n* = 44, three replicates). The same animals from Fig. 1D were used for this analysis. (**C** and **D**) Sleep onset alignment demonstrates FLP-11 release from RIS during sleep and reveals FLP-11 depletion in *nlp-99* and *npr-16* deletion mutants. Wild type (*n* = 74, five replicates), *nlp-99(syb4021)* (*n* = 62, four replicates), and *npr-16(ok1541)* (*n* = 58, four replicates). (C) FLP-11 is released from RIS during sleep. RIS calcium activity was measured using XCaMP-R. For the statistical analysis, we compared the FLP-11 sfGFP reporter before (−3 to 0 min) and after (7 to 10 min) sleep onset. (D) FLP-11 is depleted from RIS in *nlp-99* and *npr-16* deletion mutants. Wild type (mean = 1.00), *nlp-99(syb4021)* (mean = 0.53), and *npr-16(ok1541)* (mean = 0.59). The data were normalized to the wild type. Statistical significance was assessed using the Mann-Whitney *U* test for (B) and (D) and the paired Wilcoxon signed-rank test for (C). **P < 0.01 and ***P < 0.001.

Why do *nlp-99* and *npr-16* mutants sleep more during L1 arrest despite having reduced calcium activity? We hypothesized that NLP-99 and NPR-16 act downstream of RIS calcium transients, specifically at the level of neurotransmitter release from RIS. To test this hypothesis, we quantified FLP-11 expression and secretion in RIS using both transcriptional and translational reporters for FLP-11 (Fig. 3A). Expression of a transcriptional *flp-11* reporter in RIS (*32*) was significantly increased in *nlp-99* and *npr-16* mutants (Fig. 3B). It is known that increased neuronal activity is coupled to the elevated transcription of neuropeptides such as *flp-11*, potentially replenishing neuropeptides following intense secretion (*44*). Therefore, the increased transcription from the *flp-11* promoter suggests elevated FLP-11 secretion in the mutants.

To quantify FLP-11 release from RIS, we created a translational fusion allele of *flp-11* (*flp-11::sfGFP*) by inserting a gene encoding superfolder green fluorescent protein (sfGFP) into the endogenous *flp-11* locus. The *flp-11::sfGFP* knockin allele resulted in a moderate reduction in the time spent sleeping. However, it completely preserved the increased sleep phenotypes observed in *nlp-99* and *npr-16* deletion mutants (fig. S2). This indicates that the *flp-11::sfGFP* allele serves as a functional reporter in these mutants. We could not reliably quantify the sfGFP signal outside of RIS and did not detect accumulation in coelomocytes. Therefore, we focused our analysis on the sfGFP signal within RIS. The *flp-11::sfGFP* reporter localized to puncta within the RIS neurite and cell body, suggesting that sfGFP is incorporated into vesicles and co-released with FLP-11 during vesicle exocytosis (Fig. 3A). For this analysis, we combined the FLP-11 reporter with the red calcium indicator XCaMP-R (*45*) expressed in RIS and measured both the FLP-11::sfGFP and calcium sensor signals across the sleep-wake cycle during L1 arrest. In wild-type animals, the FLP-11 signal intensity in RIS increased during wakefulness and decreased during RIS calcium activation and sleep, directly demonstrating that RIS releases FLP-11 during calcium activation (Fig. 3C). Compared to the wild type, both *nlp-99* and *npr-16* mutants exhibited a strong reduction in FLP-11 reporter signal in RIS during both wakefulness and sleep (Fig. 3D). The combination of increased expression from the *flp-11* gene and reduced FLP-11 translational reporter levels in RIS in *nlp-99* and *npr-16* deletion mutants provides indirect evidence that NLP-99 and NPR-16 inhibit FLP-11 release from RIS.

We next assessed FLP-11 secretion from RIS in well-fed adults and compared it to that in L1 arrest. To do this, we quantified both our translational *flp-11::sfGFP* reporter and the transcriptional *flp-11p::mKate2* reporter in the nerve ring of *nlp-99* and *npr-16* deletion mutants. In starved larvae, both NLP-99 and NPR-16 inhibited FLP-11 secretion from RIS (fig. S3A), thus confirming our above results. However, in well-fed adults, increased secretion of FLP-11 from RIS was detectable only in *npr-16* mutants and not in *nlp-99* mutants (fig. S3B). These findings suggest that NPR-16 inhibits FLP-11 release under both nutritional conditions, whereas NLP-99 does not show this effect on well-fed adults, possibly due to functional redundancy present in fed adults but absent in L1 arrest.

### AIY neurons release NLP-99 to inhibit sleep via NPR-16

**Fig. 4.**
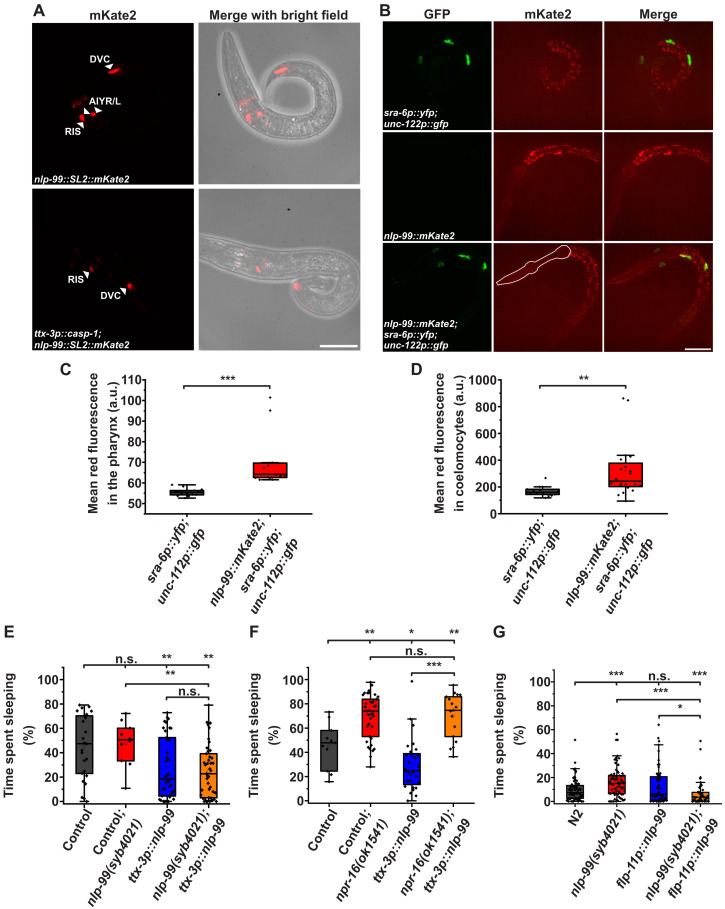
AIY neurons release NLP-99 to inhibit sleep via NPR-16. (**A**) Transcriptional reporter for *nlp-99* expression. Representative images of an arrested L1 larva. We ablated AIY by using *ttx-3p::casp-1*. The scale bar represents 25 μm. (**B**) Translational reporter for NLP-99. The pharynx is outlined with a white dashed line. We used *sra-6p::yfp,unc-122::gfp* to label coelomocytes. The scale bar represents 25 μm. (**C**) Quantification of secreted NLP-99 around the pharynx. *sra-6p::yfp,unc-122::gfp* (mean = 55.5 a.u., *n* = 15, one replicate) and *nlp-99::mKate2;sra-6p::yfp,unc-122::gfp* (mean = 69.7 a.u., *n* = 14, one replicate). (**D**) Quantification of NLP-99 in coelomocytes. *sra-6p::yfp,unc-122::gfp* (mean = 167.3 a.u., *n* = 14, one replicate) and *nlp-99::mKate2;sra-6p::yfp,unc-122::gfp* (mean = 319.4 a.u., *n* = 20, one replicate). (**E**) Quantification of sleep for *nlp-99* overexpression and rescue in AIY (*ttx-3p::nlp-99*). Control (no visible array expression) (mean = 44.7%, *n* = 25, three replicates), control;*nlp-99(syb4021)* (mean = 47.7%, *n* = 10, three replicates), *ttx-3p::nlp-99* (mean = 27.5%, *n* = 39, three replicates), and *nlp-99(syb4021);ttx-3p::nlp-99* (mean = 25.0%, *n* = 43, three replicates). (**F**) Quantification of sleep for *nlp-99* overexpression in *npr-16(ok1541)*. Control (mean = 45.3%, *n* = 11, three replicates), control *npr-16(ok1541)* (mean = 69.6%, *n* = 37, three replicates), *ttx-3p::nlp-99* (mean = 29.6%, *n* = 28, three replicates), and *npr-16(ok1541);ttx-3p::nlp-99* (mean = 70.0%, *n* = 17, three replicates). (**G**) Quantification of sleep for *nlp-99* overexpression and rescue in RIS (*flp-11p::nlp-99*). N2 (mean = 9.7%, *n* = 72, five replicates), *nlp-99(syb4021)* (mean = 16.3%, *n* = 68, five replicates), *flp-11p::nlp-99* (mean = 13.6%, *n* = 55, five replicates), and *nlp-99(syb4021);flp-11p::nlp-99* (mean = 6.4%, *n* = 52, five replicates). Mann-Whitney *U* test. *P < 0.05, **P < 0.01, and ***P < 0.001.

To investigate where NLP-99 is expressed and whether it is secreted, we generated transcriptional and translational fluorescent reporter alleles of *nlp-99* with mKate2 by editing the endogenous gene locus. We then imaged and quantified the reporter fluorescence in arrested L1 larvae. We did not detect any signal in nonneuronal cell types. The transcriptional reporter for *nlp-99* was most prominently expressed in the two AIY neurons in the head and was also clearly visible in the single DVC neuron in the tail. In addition, we observed weak expression in a single head neuron adjacent to AIYR, which is likely RIS (Fig. 4A for L1 larvae and fig. S4A for fed adults). This expression pattern is consistent with single-cell sequencing data showing that *nlp-99* is most strongly expressed in AIY neurons. *nlp-99* is the most strongly expressed gene in AIY neurons (*46*).

We observed an extracellular signal from the translational reporter at the periphery of the pharynx, as well as a signal within coelomocytes, confirmed by colocalization with a green fluorescent protein (GFP) reporter specific to coelomocytes (Fig. 4, B to D). To test the hypothesis that AIY neurons are the primary source of secreted NLP-99, we ablated these neurons (*47*). AIY ablation nearly completely abolished the extracellular signal (fig. S4, B and C). These results indicate that AIY neurons are the primary source of NLP-99 expression and secretion.

To determine whether NLP-99 acts in AIY neurons, we rescued the *nlp-99* deletion specifically in these neurons and characterized sleep during L1 arrest. We created transgenic arrays expressing *nlp-99* along with a fluorescent reporter under the control of the AIY-specific *ttx-3* promoter (*48*). We then compared the fraction of time spent sleeping and RIS calcium activity between individuals that expressed the array in AIY and those with no visible expression. Visible expression of *nlp-99* in AIY neurons significantly reduced sleep in both wild-type and *nlp-99* deletion mutant animals (Fig. 4E), suggesting that the transgenic array caused the overexpression of *nlp-99* in AIY neurons. The control *nlp-99* mutant worms that carry the array but do not show visible expression in AIY neurons did not recapitulate the increased sleep phenotype of the *nlp-99* deletion. A hypothetical explanation for this lack of phenotype is residual expression from the *nlp-99* rescue construct in these individuals. RIS activity peak alignment showed that visible *nlp-99* overexpression in AIY neurons increased the magnitude of RIS calcium activation transients (fig. S4D). Also, the frequency of RIS activation was moderately decreased (fig. S4E).

To investigate whether NLP-99 released from AIY neurons acts through NPR-16 to limit sleep and activate RIS, we tested whether the sleep inhibition caused by *nlp-99* overexpression in AIY depends on *npr-16*. Deletion of *npr-16* effectively suppressed both the sleep reduction and increased RIS activity induced by AIY-specific *nlp-99* overexpression, suggesting that NPR-16 functions downstream of AIY-released NLP-99 (Fig. 4F and fig. S4F). These results indicate that AIY neurons release NLP-99 to inhibit sleep while simultaneously promoting RIS calcium activity.

We also observed that expressing *nlp-99* in the RIS neuron using the *flp-11* promoter (*32*) suppressed the increased sleep seen in the *nlp-99* deletion mutant (Fig. 4G). This suggests that NLP-99 may contribute to RIS self-inhibition (*33*). However, given that *nlp-99* is most strongly expressed in the AIY neurons—and expression in these neurons produced the most notable phenotype—this indicates that the primary function of *nlp-99* is in AIY. Therefore, we focused our analysis on these neurons.

### The AIYs are wake-active neurons that inhibit sleep via NLP-99 and NPR-16

**Fig. 5.**
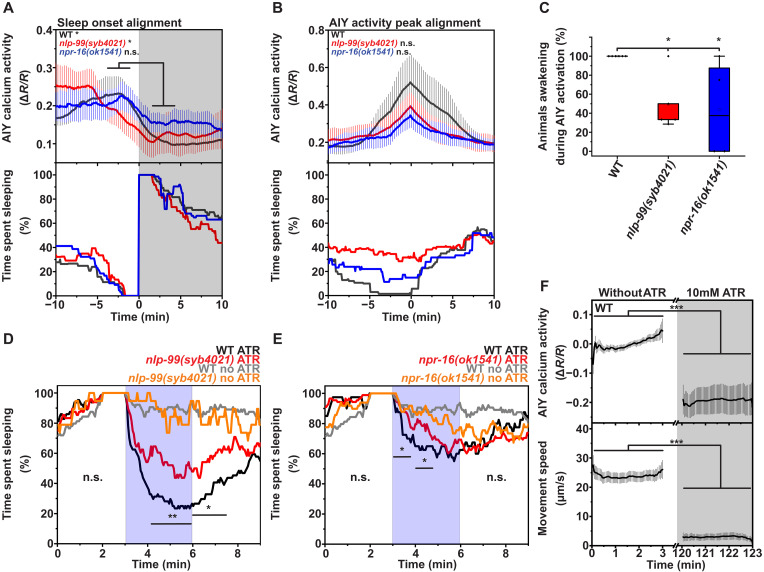
The AIYs are wake-active neurons that inhibit sleep via NLP-99 and NPR-16. (**A** to **C**) Quantification of sleep and AIY calcium activity during L1 arrest. We calculated the fraction of awake animals by identifying those that were asleep 10 min before the peak activation of AIY and assessing wakefulness at that peak. Wild type (mean = 0.52 a.u., *n* = 10, three replicates), *nlp-99(syb4021)* (mean = 0.39 a.u., *n* = 8, three replicates), and *npr-16(ok1541)* (mean = 0.34 a.u., *n* = 7, three replicates). (**D** and **E**) We assessed the waking response by selecting animals that had slept continuously for over 1 min before optogenetic AIY stimulation (shaded area) and analyzing their movement activity. (D) Assessment of *nlp-99*. Wild type with ATR (*n* = 64 cases in 36 animals, three replicates), *nlp-99(syb4021)* with ATR (*n* = 48 cases in 19 animals, three replicates), wild type without ATR (*n* = 64 cases in 29 animals, four replicates), and *nlp-99(syb4021)* without ATR (*n* = 19 cases in 13 animals, two replicates). (E) Assessment of *npr-16*. Wild type with ATR (*n* = 40 cases in 18 animals, three replicates), *npr-16(ok1541)* with ATR (*n* = 88 cases in 24 animals, three replicates), wild type without ATR (*n* = 64 cases in 29 animals, four replicates), and *npr-16(ok1541)* without ATR (*n* = 49 cases in 11 animals, two replicates). (**F**) Optogenetic activation of RIS inhibits AIY (*n* = 43, four replicates). Wilcoxon signed-rank test for (A), Mann-Whitney *U* test for (B) and (C), and Fisher’s exact test for (D) and (E) for the average of the indicated time periods for each genotype. We compared wild type with either *nlp-99(syb4021)* (D) or *npr-16(ok1541)* (E) in the presence of ATR. Paired Wilcoxon signed-rank test for (F). *P < 0.05, **P < 0.01, and ***P < 0.001.

During wakefulness, AIY neurons control locomotion by suppressing turns and reversals, which enhances smooth forward movement and dispersal (*49*, *50*). Sleep induction requires the inhibition of multiple wake-promoting neurons, and genetic analysis suggested that AIY neurons are among the wake-promoting neurons that are inhibited during sleep (*33*, *51*, *52*). Together, these findings suggest that AIY is likely more active during wakefulness and less active during sleep. To test this hypothesis, we measured AIY activity and assessed its correlation with sleep. We used a transgene that expresses GCaMP specifically in AIY (*53*) and imaged calcium activity during L1 arrest across sleep and wakefulness cycles in wild-type, *nlp-99(syb4021)*, and *npr-16(ok1541)* animals. We first aligned all sleep bouts to their onset and quantified the corresponding AIY activity. During sleep, AIY activity was reduced, indicating that AIY is a wake-active neuron (Fig. 5A). Overall, the pattern of AIY neuronal activity during the sleep-wake cycle did not appear to be significantly altered by these mutations (fig. S5, A to C).

To investigate how AIY activation correlates with sleep, we extracted and aligned all AIY activation transients to their maxima and calculated the corresponding fraction of animals that were asleep during AIY activation. Until ~10 min before the AIY calcium activity peak, 30% of wild-type larvae were asleep. All animals were awake at the peak, but 10 min afterward, the likelihood of sleep increased to around 40%. In contrast, in *nlp-99* mutant animals, the percentage of sleeping animals remained relatively constant at about 40% with no noticeable decrease at the AIY activity peak. *npr-16* mutant animals also showed an increased probability of being asleep at the AIY activity peak, although this effect was less pronounced compared to *nlp-99* mutant animals, perhaps due to additional neurons and receptors influencing RIS (Fig. 5, B and C). These results characterize AIY as a wake-active neuron and suggest that AIY activation inhibits sleep and promotes wakefulness through NLP-99 and NPR-16.

To directly test whether AIY activation inhibits sleep via NLP-99 and NPR-16, we optogenetically activated AIY during sleep in wild-type, *nlp-99*, and *npr-16* mutant animals. We used a transgene expressing channelrhodopsin in AIY neurons (*54*), activated these neurons with blue light during sleep, and measured the fraction of animals that awoke upon stimulation. In wild-type animals, AIY activation reliably woke the animals. In contrast, AIY activation induced awakening in fewer than half of the trials in *nlp-99* and *npr-16* mutants, with the phenotype being more pronounced in *nlp-99* mutants (Fig. 5, D and E).

To test whether RIS activation inhibits AIY neurons, we optogenetically activated RIS and measured calcium activity in AIY. For this experiment, we generated a transgene expressing ReaChR under the control of a minimal *flp-11* promoter fragment (fig. S6) and combined it with a transgene expressing GCaMP in AIY neurons (*53*). To prevent RIS activation during the baseline recording, we first measured calcium activity in the absence of retinal. We then added retinal to enable optogenetic activation of RIS. RIS activation significantly reduced AIY calcium activity (Fig. 5F), demonstrating that RIS inhibits AIY.

In summary, AIY activation induces wakefulness through NLP-99 and NPR-16. Conversely, RIS inhibits the AIY neurons. This suggests that RIS and AIY may mutually inhibit each other, functioning as a flip-flop switch.

### NPR-16 exerts cell-autonomous and nonautonomous control over RIS calcium activity and sleep

**Fig. 6.**
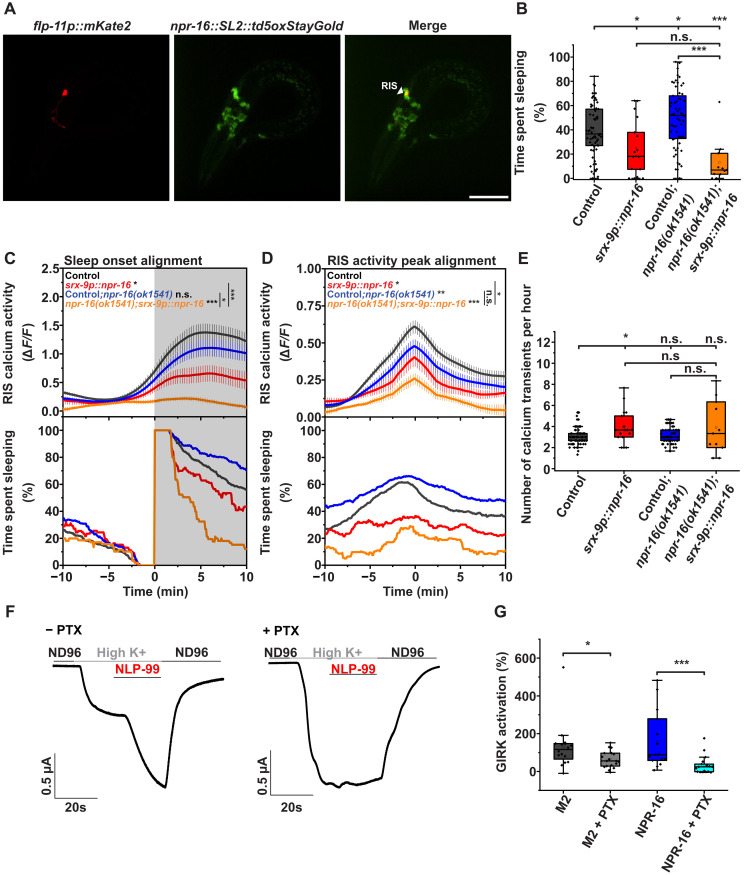
NPR-16 is a G_i/o_-coupled receptor that acts in RIS to limit sleep. (**A**) *npr-16* expression in neurons during L1 arrest. We combined *npr-16::SL2::td5oxStayGold* with *flp-11p::mKate2* to identify RIS. The scale bar represents 25 μm. (**B** to **E**) Overexpression of *npr-16* in RIS during L1 arrest. Control (no visible array expression) (*n* = 65, three replicates), *srx-9p::npr-16* (*n* = 17, three replicates), control;*npr-16(ok1541)* (*n* = 58, three replicates), and *npr-16(ok1541);srx-9p::npr-16* (*n* = 11, three replicates). (B) Time spent sleeping. Control (mean = 39.2%,), *srx-9p::npr-16* (mean = 24.9%), control;*npr-16(ok1541)* (mean = 49.8%), and *npr-16(ok1541);srx-9p::npr-16* (mean = 13.2%). (C) RIS calcium activity assessed by sleep bout alignment. Control (mean = 1.43 a.u.), *srx-9p::npr-16* (mean = 0.72 a.u.), control;*npr-16(ok1541)* (mean = 1.17 a.u.), and *npr-16(ok1541);srx-9p::npr-16* (mean = 0.24 a.u.). (D) RIS calcium activation peak alignment. Control (mean = 0.61 a.u.), *srx-9p::npr-16* (mean = 0.47 a.u.), control;*npr-16(ok1541)* (mean = 0.47 a.u.), and *npr-16(ok1541);srx-9p::npr-16* (mean = 0.25 a.u.). (E) Number of RIS calcium transient peaks for overexpression of *npr-16* in RIS. Control (mean = 3.1 peaks per hour), *srx-9p::npr-16* (mean = 4.0 peaks per hour), control*;npr-16(ok1541)* (mean = 3.2 peaks per hour), and *npr-16(ok1541);srx-9p::npr-16* (mean = 3.9 peaks per hour). (**F** and **G**) NPR-16 coupling to G_i/o_. (F) Representative traces of TEVC recordings from untreated (left) and PTX-injected (right) *X. laevis* oocytes, which express NPR-16, mGIRK1, and mGIRK2 and were treated with 1 μM NLP-99 peptide. (G) We measured GIRK activation in at least 15 oocytes per condition across two replicates. Statistical significance was assessed using the Mann-Whitney *U* test for (B) to (E) and (G). *P < 0.05, **P < 0.01, and ***P < 0.001.

Single-cell sequencing of developing larvae revealed that *npr-16* is expressed in at least 50 different neuronal cell types, including RIS (*46*). To determine whether *npr-16* is expressed in RIS during L1 arrest, we generated a transcriptional reporter by knocking a gene encoding Td5oxStayGold (*55*) into the endogenous *npr-16* locus, along with an SL2 trans-splicing sequence. We then crossed this reporter allele with a transgene that expresses mKate2 in RIS (*32*). In L1 arrest, *npr-16* was expressed in multiple neurons in the head and tail, with one of the strongest expressions observed in RIS (Fig. 6A).

To test whether NPR-16 acts in RIS to limit sleep, we generated a multicopy extrachromosomal array to express *npr-16* in RIS using the *srx-9* promoter (*33*, *46*). As before, we compared sleep and RIS calcium activity between individuals carrying the array in RIS and those without it. Expression of *npr-16* reduced sleep during L1 arrest in wild-type animals, indicating overexpression. In the *npr-16* mutant, the overexpression array suppressed sleep even more strongly than in the wild type (Fig. 6B). NLP-99 is likely bound by NPR-16 in many neurons, which may lead to a greater availability of free NLP-99 in the *npr-16* mutant. This may explain the stronger effect of NPR-16 overexpression in RIS in the *npr-16* mutant.

*npr-16* overexpression reduced the magnitude of RIS calcium activation transients. In *npr-16* mutant larvae, the reduced RIS calcium phenotype was not rescued by the RIS-specific expression of *npr-16* but instead was further reduced (Fig. 6, C to E). These results suggest that NPR-16 exerts both cell-nonautonomous and cell-autonomous effects on RIS calcium activity and sleep. The activating effect of *npr-16* on RIS calcium does not arise from its function within RIS itself but rather from its action in other neurons. In contrast, NPR-16 acts within RIS to limit RIS calcium activity and sleep.

In fed adults, *npr-16* was still expressed in RIS. However, its expression in RIS was no longer among the strongest, with multiple head neurons expressing *npr-16* at comparable or higher levels (fig. S7). RIS-specific expression of *npr-16* caused significantly reduced sleep and did not rescue the reduced sleep phenotype of *npr-16* deletion mutant adults (fig. S8). These results support the view that reduced sleep in *npr-16* deletion adults arises primarily from the loss of *npr-16* function in non-RIS neurons, while NPR-16 acts within RIS to inhibit sleep.

### NPR-16 is a G_i/o_-coupled receptor

All five mammalian somatostatin receptors are coupled to the G_i/o_ protein pathway, which inhibits adenyl cyclase, reducing cAMP production (*24*). Increased cAMP triggers two distinct processes that can each promote vesicle release: (i) It elevates intracellular calcium levels, and (ii) it independently enhances dense-core vesicle (DCV) release directly (*56*). Thus, a plausible hypothesis is that NLP-99 activates NPR-16 in RIS to promote G_i/o_ signaling, which inhibits cAMP and consequently reduces both intracellular calcium and the release of FLP-11–containing DCVs. To determine the cellular response of NPR-16 to NLP-99 activation, we expressed this GPCR in *Xenopus laevis* oocytes and measured its activation upon NLP-99 exposure using two-electrode voltage-clamp (TEVC) recordings (*28*). As expected, NPR-16 strongly responded to its ligand NLP-99, confirming their interaction as a peptide-GPCR pair in heterologous cells (Fig. 6F). Administration of pertussis toxin (PTX) diminished the activation of NPR-16, similar to the known G_i/o_-coupled muscarinic M2 receptor (Fig. 6, F and G) (*57*), indicating that NPR-16 is also G_i/o_ coupled. These results are consistent with, and provide a mechanistic basis for, the inhibitory effects of NLP-99 and NPR-16 on RIS calcium activation and FLP-11 release from RIS. Because NPR-16 is G_i/o_ coupled, its activating influence on RIS from outside of RIS may result from inhibition of upstream neurons that normally suppress RIS.

### NLP-99 and NPR-16 limit survival and lifespan through RIS inhibition

**Fig. 7.**
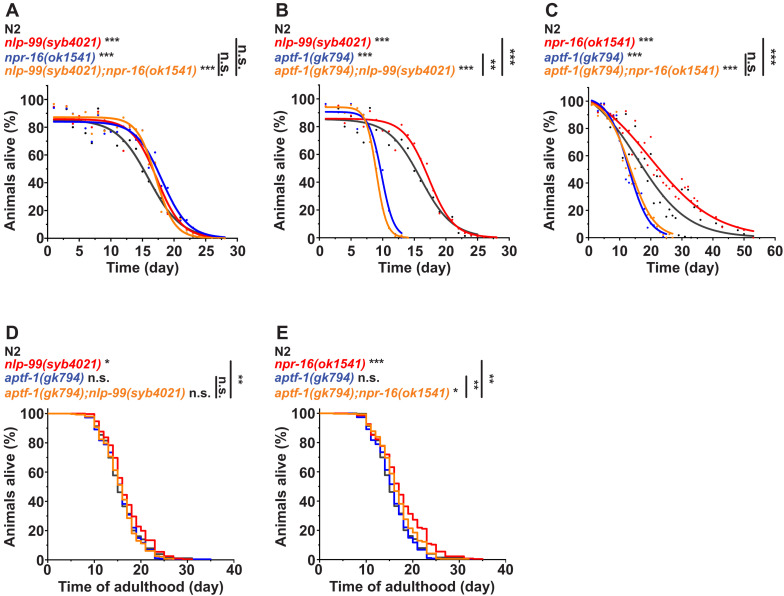
NLP-99 and NPR-16 limit L1 arrest survival and adult lifespan through RIS. (**A** to **C**) L1 arrest survival experiments for *nlp-99*, *npr-16*, and *aptf-1* deletion mutants [(A) and (B): three replicates; (C): five replicates]. (**D** and **E**) Adult lifespan. We performed six lifespan assays in parallel in adult worms on NGM plates. Deletion of *nlp-99* extended the mean lifespan by ~1 day [mean lifespans: N2: 15.81 days; *nlp-99(syb4021)*: 16.69 days], an effect that fully depended on *aptf-1* [mean lifespans: *aptf-1(gk794)*: 15.67 days; *aptf-1(gk794);nlp-99(syb4021)*: 15.56 days], indicating that the lifespan extension was fully caused by RIS (three replicates). Deletion of *npr-16* extended the mean lifespan by ~2 days [mean lifespan *npr-16(ok1541)*: 17.48 days], an effect that was half dependent on *aptf-1* [mean lifespans: *aptf-1(gk794)*: 15.67 days; *aptf-1(gk794);npr-16(ok1541)*: 16.62 days], indicating that 1 day of the lifespan extension was caused by RIS and the other day of extension is attributed to an RIS-independent effect (three replicates). Statistical significance was assessed using Fisher’s exact test for (A) to (C) and the log-rank test for (D) and (E). *P < 0.05, **P < 0.01, and ***P < 0.001.

Sleep is crucial for many essential physiological processes. In *C. elegans*, it counteracts aging phenotypes, promotes survival during L1 arrest, and is required for survival after wounding and stress in adults (*8*, *9*, *35*, *39*). On the basis of these findings, we hypothesized that *nlp-99* and *npr-16* mutants, with increased FLP-11 release from RIS and extended sleep, would display enhanced survival and lifespan. To test this hypothesis, we first examined whether *nlp-99* and *npr-16* deletions increase L1 arrest survival through RIS. We cultured populations of arrested L1 larvae and regularly measured the fraction of surviving animals (*8*, *39*, *58*). Both *nlp-99* and *npr-16* mutants showed a significant increase in L1 arrest survival of about 15%, and this effect was not further enhanced by combining the two mutants (Fig. 7A). Using *aptf-1* deletion showed that the survival extension was entirely dependent on functional RIS (Fig. 7, B and C). We next tested the lifespan in the adult in the presence of food. Deletion of *nlp-99* increased the mean lifespan by ~1 day, while deletion of *npr-16* extended the mean lifespan by ~2 days. The 1-day lifespan extension observed with *nlp-99* deletion was fully dependent on functional RIS. In contrast, the 2-day lifespan extension resulting from *npr-16* deletion was partially dependent on functional RIS, with 1 day (i.e., half of the extension) attributable to RIS (Fig. 7, D and E). In summary, NLP-99 and NPR-16 limit survival and lifespan via RIS.

### NPR-16 limits intestinal lipid stores during L1 arrest through RIS

Somatostatin plays a conserved role in limiting anabolic processes such as lipid storage (*21*, *22*), while sleep promotes anabolic activities (*59*, *60*). Our finding that NLP-99/NPR-16 inhibits neurotransmission from RIS suggests that it may restrict anabolic metabolism by suppressing RIS. To explore this hypothesis, we examined the impact of somatostatin signaling and RIS on intestinal lipid stores.

**Fig. 8.**
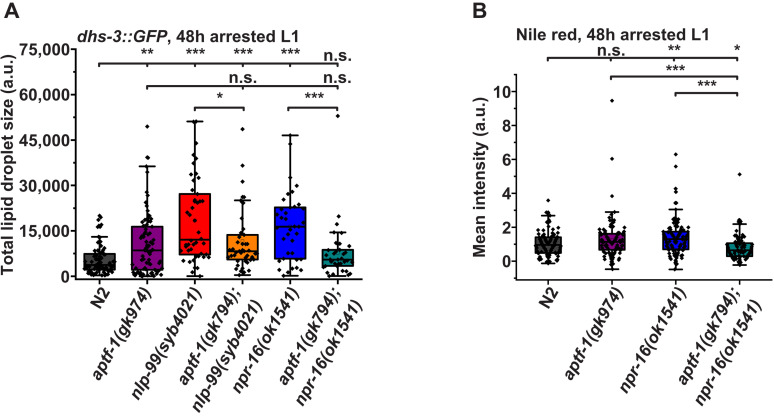
NLP-99 and NPR-16 limit intestinal lipid stores through RIS. (**A**) Analysis of the DHS-3::GFP reporter transgene reveals that NLP-99 and NPR-16 limit lipid storage during L1 arrest through RIS. N2 [total lipid droplet size (LS) = 5528 a.u., *n* = 83, six replicates], *aptf-1(gk794)* (LS = 10629 a.u., *n* = 85, six replicates), *nlp-99(syb4021)* (LS = 18379 a.u., *n* = 46, three replicates), *aptf-1(gk794);nlp-99(syb4021)* (LS = 11588 a.u., *n* = 45, three replicates), *npr-16(ok1541)* (LS = 16432 a.u., *n* = 37, three replicates), and *npr-16(ok1541);aptf-1(gk794)* (LS = 7731 a.u., *n* = 37, three replicates). (**B**) Nile Red staining reveals that *npr-16* limits lipid storage during L1 arrest through RIS. We normalized data to N2. N2 (mean = 1.00 a.u., *n* = 103, three replicates), *aptf-1(gk794)* (mean = 1.25 a.u., *n* = 109, three replicates), *npr-16(ok1541)* (mean = 1.36 a.u., *n* = 108, three replicates), and *aptf-1(gk794);npr-16(ok1541)* (mean = 0.80 a.u., *n* = 99, three replicates). Statistical significance was assessed using the Mann-Whitney *U* test. *P < 0.05, **P < 0.01, and ***P < 0.001.

We first visualized intestinal lipids using a genetically encoded reporter for lipid droplets (DHS-3::GFP), which has been established for studying lipid content in sleep mutants (*61*). We crossed this reporter into *nlp-99* and *npr-16* mutant backgrounds and quantified intestinal lipid droplets in arrested L1 larvae. *nlp-99* and *npr-16* mutants exhibited substantially increased lipid stores. To test whether this effect was mediated by RIS, we also introduced *aptf-1* deletion into these strains. Deletion of *aptf-1* alone caused a modest increase in intestinal lipids, and the elevated lipid stores in *nlp-99* and *npr-16* mutants depended on *aptf-1* (Fig. 8A and fig. S9A).

We then confirmed the results from the translational lipid reporter by using Nile Red staining (*62*) in *npr-16* mutant arrested L1 larvae and again tested whether this effect was mediated by RIS. Although arrested L1 larvae generally have low lipid stores, Nile Red staining enabled clear visualization and quantification of intestinal lipids (fig. S9B). We observed a trend toward increased lipid levels in *aptf-1* deletion mutant larvae, but this did not reach statistical significance. In contrast, *npr-16* deletion significantly increased intestinal lipid stores during larval starvation. This increase was dependent on functional RIS (Fig. 8B). These findings indicate that NPR-16 regulates intestinal lipid stores during L1 arrest through RIS. The enhanced lipid storage may contribute to the extended survival observed in *npr-16* mutants.

We also quantified lipid levels using Nile Red in well-fed wild-type and *npr-16* deletion mutant adult animals. Deletion of *npr-16* increased lipid stores, but this effect did not appear to depend on RIS, as indicated by *aptf-1* deletion. Moreover, lipid levels were even higher in the double mutant (fig. S9, C and D), supporting the conclusion that RIS and NPR-16 act through separate pathways to suppress lipid storage in well-fed adults.

### RIS/FLP-11 and NLP-99/NPR-16 are both necessary for adult memory consolidation

In mammals, somatostatin and somatostatin-expressing neurons are required for memory formation (*21*, *23*). A role for somatostatin signaling in memory has not yet been examined in *C. elegans*. In *C. elegans*, learning and memory studies are primarily conducted in adults and often focus on adult olfactory learning and memory—a well-characterized process known to involve the AIY interneurons (*63*–*65*). A recent study suggested that olfactory conditioning in adult *C. elegans* increases sleep, which in turn supports memory formation (*65*). However, the role of the core sleep neuron RIS in memory is still unknown. The major types of behavioral quiescence in adult *C. elegans*—including spontaneous sleep on food (*8*), starvation-induced sleep (*8*, *37*), satiety quiescence (*66*), stress-induced sleep (*36*, *61*), and locomotion pauses (*43*)—are all known to depend on RIS. This suggests that if sleep contributes to memory formation, RIS would likely be required.

**Fig. 9.**
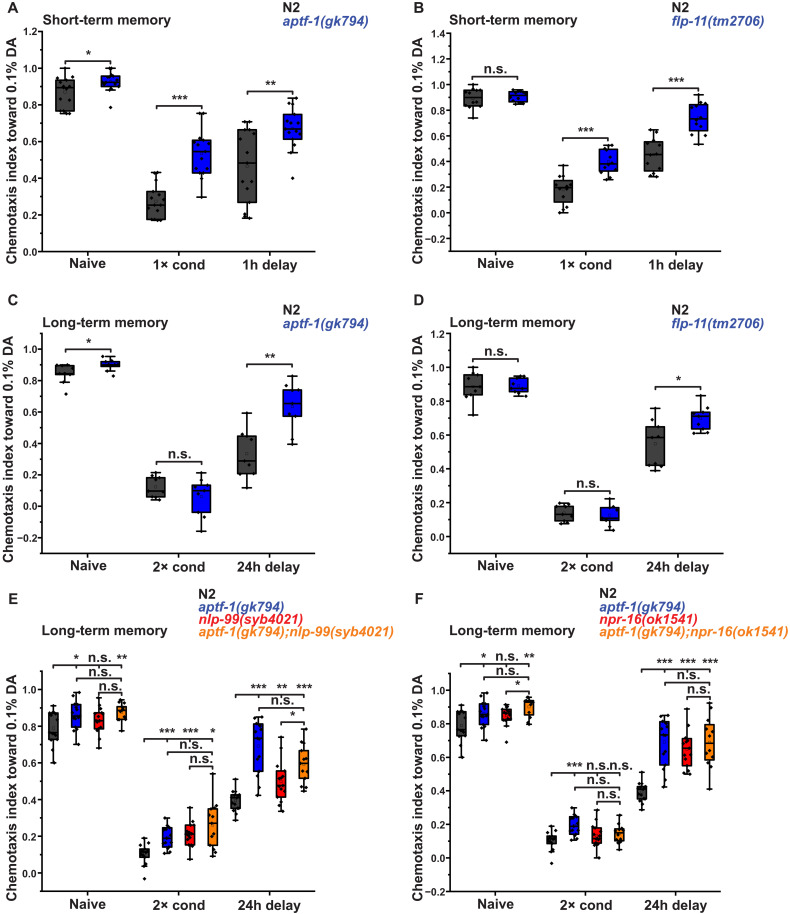
NLP-99/NPR-16 and RIS/FLP-11 work in concert to support memory consolidation at the adult stage. (**A** and **B**) STM assays in mutants with reduced sleep. *aptf-1* (15 replicates) and *flp-11* (12 replicates) are required for STM formation, suggesting that FLP-11 released by RIS during sleep is essential for learning. (**C** and **D**) LTM assays in mutants with reduced sleep. *aptf-1* (nine replicates) and *flp-11* (nine replicates) are required for LTM, suggesting that FLP-11 released by RIS during sleep is essential for memory retention. (**E** and **F**) LTM assays in *nlp-99* and *npr-16* mutants. *nlp-99* (15 replicates) and *npr-16* (15 replicates) are required for LTM and act in concert with RIS. Statistical significance was assessed using the Mann-Whitney test. *P < 0.05, **P < 0.01, and ***P < 0.001.

To determine whether RIS is involved in learning and memory, we first tested *aptf-1* and *flp-11* deletion mutants using a short-term memory (STM) assay (*67*). In this assay, negative conditioning to diacetyl (DA), a normally attractive chemical cue, is induced in well-fed adults by a brief starvation stimulus during DA exposure, and STM is tested in the presence of food 1 hour after conditioning (*67*). Both *aptf-1* and *flp-11* mutants exhibited significant learning deficits, suggesting that RIS and FLP-11 are required for adult learning (Fig. 9, A and B).

Because of the learning defect in the RIS mutants, the STM assay was not suitable for reliably measuring memory. Therefore, we used an LTM assay involving two rounds of conditioning and testing for LTM after 24 hours (*67*). After repeated conditioning, both *aptf-1* and *flp-11* mutants exhibited only a mild learning deficit, allowing for LTM testing. In the LTM assay, both mutants showed a strong impairment (Fig. 9, C and D).

Next, we evaluated LTM in *nlp-99* and *npr-16* mutants. Both *nlp-99* and *npr-16* mutants showed impaired memory consolidation, with *npr-16* deletion causing a more pronounced effect than *nlp-99* deletion. Combined deletion of *aptf-1* and *nlp-99* resulted in an intermediate phenotype, while combined deletion of *npr-16* and *aptf-1* produced a phenotype similar to that of the single mutants (Fig. 9, E and F, and fig. S10). These findings indicate that *aptf-1*/*flp-11* and *nlp-99*/*npr-16* are both necessary for memory formation and act within the same pathway, potentially through controlling RIS activity and sleep.

## DISCUSSION

While somatostatin generally has an inhibitory effect on mammalian sleep (*17*–*19*), neurons coexpressing somatostatin and GABA can also promote cortical down states during NREM sleep following their activation (*15*, *16*). We found that the *C. elegans* somatostatin-like neuropeptide NLP-99 and its receptor NPR-16 inhibit sleep during L1 arrest and in starved adults but increase sleep in well-fed adults, supporting the view that sleep regulation is a complex, context-dependent, and evolutionarily conserved function of somatostatin.

GCaMP imaging revealed that both NLP-99 and NPR-16 increase RIS calcium activity during L1 arrest and in well-fed adults, with a stronger effect on well-fed adults. In well-fed adults, NPR-16 also increased the frequency of RIS calcium transients. Rescue experiments expressing *npr-16*, specifically in RIS, did not restore the decreased RIS calcium activity seen in *npr-16* mutant arrested larvae. Instead, RIS-specific expression of *npr-16* further reduced RIS calcium levels and sleep, indicating that NPR-16 acts within RIS to inhibit calcium activation and sleep. These results indicate that the overall activating effect of NPR-16 on RIS calcium originates from its action in other neurons. Because NPR-16 is G_i/o_ coupled and thus inhibitory, a plausible hypothesis is that NPR-16 inhibits neurons that themself inhibit RIS. Future experiments will be needed to test this hypothesis and identify the relevant cell types.

**Fig. 10.**
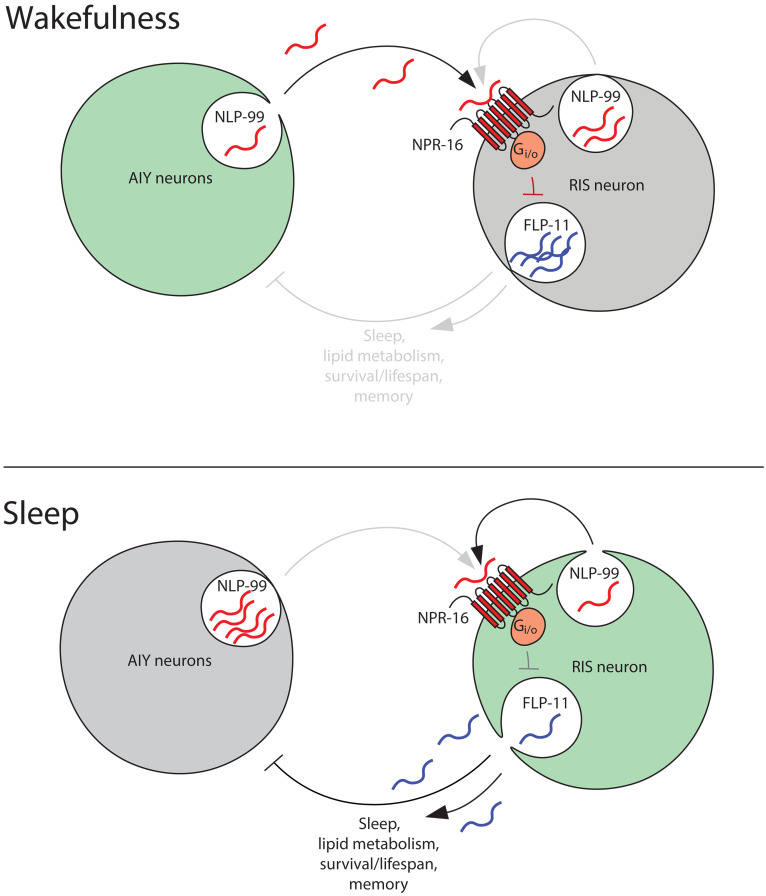
*C. elegans* somatostatin signaling controls sleep, lipid storage, survival, and longevity through regulation of the sleep-active RIS neuron. Our model, based primarily on L1 arrest data, proposes that during wakefulness, AIY releases NLP-99, which acts on RIS via two pathways. (i) NLP-99 activates NPR-16 in non-RIS neurons (top), which are inhibitory. Because NPR-16 is G_i/o_ coupled, it may inhibit neurons that themselves inhibit RIS, resulting in a net activation of RIS. (ii) NLP-99 directly activates NPR-16 in RIS, reducing calcium activity and FLP-11 release. When RIS becomes active, it releases FLP-11, which inhibits wakefulness-promoting neurons such as the AIYs and therefore induces sleep. Through mutual inhibition, RIS and the AIYs function as a flip-flop switch. In addition to FLP-11, RIS also releases NLP-99, which may act in a self-inhibitory feedback loop to limit sleep. Thus, NLP-99 and NPR-16 act both within and outside of RIS in a complex interplay to control RIS activity and sleep. The circuit appears similar in L1 arrest and fed adults, but the relative contributions of direct and indirect effects differ. During L1 arrest, the phenotype is dominated by inhibitory NPR-16 signaling within RIS, whereas in fed adults, it is dominated by activating NPR-16 signaling outside of RIS. One possible mechanism is differential NPR-16 expression: During L1 arrest, RIS shows strong NPR-16 expression, whereas in fed adults, other neurons express it more strongly. RIS activity and FLP-11 release promote survival during L1 arrest and extend lifespan in fed adults. RIS also regulates lipid metabolism and adult memory. Although NLP-99/NPR-16 signaling may regulate lipid metabolism partly independently of RIS, most physiological effects appear mediated through RIS regulation.

How can the different sleep phenotypes of *nlp-99* and *npr-16* deletion mutants during L1 arrest and in fed adults be explained? According to our model (Fig. 10), NPR-16 exerts an activating effect on RIS by acting outside of RIS and an inhibitory effect within RIS. During L1 arrest, *npr-16* expression in RIS is among the strongest, whereas in adults, its strongest expression occurs in non-RIS neurons. This suggests that the activating role of NPR-16 in non-RIS neurons predominates in fed adults but is less important during L1 arrest, during which the inhibitory effect of *npr-16* within RIS dominates. Future experiments will be required to test how developmental stages and food conditions control NPR-16 expression to modulate the sleep circuit.

To investigate why NLP-99 and NPR-16 inhibit sleep during L1 arrest despite modestly reduced RIS calcium activity, we next examined RIS neurotransmission. To do this, we generated a reporter for FLP-11 secretion. However, we were unable to directly quantify the release of individual FLP-11–containing DCVs or detect the FLP-11::sfGFP signal outside of RIS. Therefore, we focused our analysis on the FLP-11::sfGFP signal within RIS. In *nlp-99* and *npr-16* deletions during L1 arrest, we observed depletion of FLP-11::sfGFP in RIS along with increased *flp-11* promoter activity, suggesting that NLP-99 and NPR-16 inhibit the release of sleep-inducing FLP-11 neuropeptides. In well-fed adults, this increase was detectable only in *npr-16* mutants. This is consistent with generally weaker phenotypes for *nlp-99* compared with *npr-16*, particularly in well-fed adults, suggesting that a functional redundancy of *nlp-99* may exist.

Because FLP-11 release was increased despite reduced RIS calcium, these findings indicate that NPR-16 activation regulates FLP-11 release directly, downstream of RIS calcium activation. This cellular mechanism resembles the known action of somatostatin signaling (*56*) and contrasts with previously described pathways that regulate sleep by modulating RIS at the level of its calcium levels (*40*, *52*).

While NLP-99/NPR-16 inhibits FLP-11 release from RIS, FLP-11 in turn induces sleep and silences many wakefulness-promoting neurons through FLP-11 signaling (*31*, *32*, *39*, *40*, *51*, *68*). Consistent with this role of RIS, we observed that AIY neuron activity decreases during sleep and upon optogenetic activation of RIS. This mutual inhibition between AIY and RIS functions analogously to a flip-flop switch, ensuring distinct sleep and wakefulness states (*11*).

The AIY neurons are not presynaptic to RIS (*69*, *70*). However, RIS and AIY neurites are physically close in the nerve ring (*71*–*73*). This proximity suggests that AIY may signal to RIS through a nonsynaptic mechanism, possibly using NLP-99 as a paracrine neurohormone (*74*, *75*).

While FLP-11 is the primary neurotransmitter responsible for sleep induction by RIS, one or more additional transmitters likely contribute to this process (*32*, *39*). Consistent with this idea, RIS also expresses GABA [a transmitter involved in quiescence (*43*)] and several other neuropeptides (*36*, *46*). Although a sleep-inducing role for these transmitters has not yet been identified in RIS, the slight increase in sleep observed in the *npr-16* mutant lacking functional *flp-11* suggests the existence of at least one additional transmitter whose release from RIS is also regulated via NPR-16. Future studies will be needed to identify potential additional sleep-promoting transmitters in RIS and determine how their release is controlled by NLP-99/NPR-16.

During L1 arrest, NLP-99 acts primarily from the AIY interneurons, a cell type that does not express GABA (*46*, *76*). During wakefulness, AIY neurons are active and release NLP-99, which activates NPR-16 in the sleep-promoting neuron RIS, thereby suppressing neurotransmission from RIS. We found that NLP-99 also inhibits sleep when expressed in RIS, the only cell type that coexpresses GABA and NLP-99 (*46*, *76*). NLP-99 secretion from RIS during calcium activation may thus contribute to RIS self-inhibition (*33*, *40*). These findings indicate that NLP-99 functions as a general sleep inhibitor during L1 arrest, regardless of the cell type in which it is expressed.

It will be interesting to test whether mammalian somatostatin similarly controls sleep neurons that express somatostatin receptors. A self-inhibitory role for somatostatin signaling may also be conserved in mammals. In the cortex, GABAergic and somatostatin-positive neurons, which are required for slow oscillations (*15*), also express somatostatin receptors (*77*). Thus, activation of these neurons during cortical up states and the subsequent release of somatostatin could lead to self-inhibition during the down state, thereby contributing to slow oscillations.

Here, we show that NLP-99 and NPR-16 reduce both L1 arrest survival and adult lifespan through RIS. We previously demonstrated that the survival benefits typically associated with sleep arise from RIS activation and FLP-11 release and occur independently of behavioral quiescence (*39*). Although fed adults and starved larvae show opposing sleep phenotypes upon *nlp-99* and *npr-16* deletion, they exhibit similar effects on survival. This suggests that the key determinant of lifespan extension is not behavioral sleep but rather RIS function itself, possibly mediated through FLP-11 secretion. Investigating the role of FLP-11 release in aging therefore represents an interesting avenue for future research.

Somatostatin inhibits anabolic processes such as lipid storage, thereby preventing obesity in older age (*2*, *24*). Consistent with this finding, we show that NLP-99 and NPR-16 inhibit lipid storage. This supports the idea that the inhibition of lipid storage is an evolutionarily conserved function of somatostatin signaling. During L1 arrest, RIS modestly inhibits lipid stores, and NLP-99/NPR-16 strongly suppresses lipid metabolism in a manner that requires RIS. This suggests that under normal L1 arrest conditions, RIS acts to limit lipid accumulation, whereas under strong activation, it may instead promote lipid storage. In well-fed adults, NPR-16 also suppresses lipid storage, but this effect appears to be independent of RIS. Instead, NPR-16 may act primarily via non-RIS neurons to control lipid storage in fed adults. This idea is consistent with NPR-16 being expressed more strongly in non-RIS neurons than in RIS. Notably, RIS inhibits lipid storage only when *npr-16* is deleted, that is, when lipid storage is disinhibited. This interaction supports the idea that RIS and NPR-16 act synergistically to regulate lipid stores, with NPR-16 providing the dominant suppressive input in fed adults. Future experiments will be required to identify the neurons through which NPR-16 acts in fed adults and to determine how these neurons interact with RIS.

Consistent with the role of sleep in memory formation (*65*), we demonstrate that RIS is required for adult memory in *C. elegans*. RIS is active during various types of behavioral quiescence, including locomotion pauses (*43*), spontaneous sleep on food (*8*), starvation-induced sleep (*8*, *37*), satiety-induced quiescence (*66*), and stress-induced sleep (*36*, *61*). Olfactory conditioning has been suggested to increase sleep (*65*), and the conditioning protocol includes several conditions in which RIS is expected to be active. This makes it challenging to determine the specific timing at which RIS activation is required for learning and memory. Investigating whether RIS/FLP-11 acts before, during, or after conditioning represents an important direction for future studies.

Somatostatin promotes memory formation in mammals (*21*–*23*), and we found that NLP-99/NPR-16 is required for adult sleep. Consistent with these findings, we show that NLP-99 and NPR-16 are required for adult memory in *C. elegans*, indicating that memory promotion is an evolutionarily conserved function of somatostatin signaling. The precise timing of NLP-99/NPR-16 involvement relative to conditioning remains unknown. Nevertheless, our data show that both RIS/FLP-11 signaling and NLP-99/NPR-16 signaling are required for sleep and memory. This suggests that behavioral sleep, rather than FLP-11 release alone, may be critical for memory regulation. These findings provide a foundation for future mechanistic studies on the role of RIS and sleep in *C. elegans* memory.

Our data indicate that NLP-99 and NPR-16 exert their control over lifespan and memory by regulating RIS activity and sleep. Therefore, the physiological effects of somatostatin/AstC signaling arise primarily from the control of RIS activity. Given its high evolutionary conservation, somatostatin/AstC signaling likely regulates sleep, survival, metabolism, and memory via sleep neurons in other species as well.

## MATERIALS AND METHODS

### Experimental design

We carried out all in vivo experiments using *C. elegans*. We used arrested L1 or adult hermaphrodites for this study. We performed the in vitro experiments using *Xenopus* oocytes.

### Cultivation and preparation of *C. elegans* strains

We cultured *C. elegans* strains on NGM plates seeded with *Escherichia coli* OP50 and maintained them at 20°C. A list of the strains used for this study can be found in table S1.

### Crossing and genotyping

We combined different alleles and transgenes using standard crossing methods. We genotyped the animals using visible phenotypic markers and polymerase chain reaction according to standard protocols. A list of the primers we used can be found in table S2.

### DNA constructs and extrachromosomal array generation

To drive the expression of *nlp-99* in AIY neurons, we used two fragments of the *ttx-3* gene, which have been shown previously to be required for strong and specific expression in AIY neurons (*48*). We fused 636 base pairs (bp) upstream of the main ATG of *ttx-3* with the second intron (243 bp) of *ttx-3* to generate a promoter sequence. We codon optimized the coding sequence of *nlp-99* to a Codon Adaptation Index (CAI) of 1.0 (*78*). Behind the *nlp-99* genes, we inserted the SL2 trans splice sequence of *gpd-2*, followed by codon-optimized (CAI1.0) mKate2 carrying two introns (*78*) and then the 3′ untranslated region (3′UTR) of *unc-54* (data S1). The construct was synthesized and cloned into pUC57-Mini by GenScript to create pUC57-Mini-ttx-3p::nlp-99::SL2::mKate2::unc-54-3′UTR. We injected this plasmid into N2 at a concentration of 40 ng/μl along with pCG150 at a concentration of 100 ng/μl to create *goeEx744[ttx-3p::nlp-99-SL2-mKate2::unc-54 3′UTR]*. The strongest expression in *goeEx744* was visible in AIY neurons. As controls, individual animals of this strain were used that did not express the array in the AIY neurons.

To drive the expression of *npr-16* in the RIS neuron, we used the upstream promoter sequence of the *srx-9* gene. *srx-9* has been shown by single-cell sequencing and site-specific recombination experiments to be expressed mostly in RIS, while it may be expressed at lower levels in a few other neurons as well as in nonneuronal tissue (*33*, *46*). We had 2000 bp that is upstream of the *srx-9* ATG cloned by SunyBiotech from genomic N2 DNA, together with the *npr-16a* cDNA, SL2 trans splice sequence of *gpd-2*, codon-optimized (CAI1.0) mKate2 carrying two introns (*78*), and the 3′UTR of *let-858* into pPD95.77 to generate pHB86 (*srx-9p::npr-16a::SL2::mKate2::let-858 3′UTR*) (data S1). The plasmid was injected at 30 ng/μl together with pCFJ350 (25 ng/μl) into *unc-119(ed9)* to create *goeEx765*. We crossed this reporter array into the RIS transgenic reporter *goeIs345[flp-11p::SL1-GCaMP3.35-SL2::flp-11 3′UTR, unc-119(+)]* and detected expression from the *srx-9* promoter in RIS but also in other cell types. For experiments, individual animals were selected that expressed the array in RIS. As controls, animals from the same strain were used that did not express the array in RIS.

### Genome editing using CRISPR

To generate the *nlp-99* deletion allele *syb4021*, 85 bp of the first exon of the coding sequence of T05A8.3.1 was deleted, and two stop codons were introduced by SunyBiotech (for details on the sequences used for the editing, see table S3). This deletion removed the signal sequence and all in-frame start codons so that the NLP-99 peptide, which is encoded in the second exon, should not be formed. The deletion hence should present a molecular null allele (data S1).

The *nlp-99(ibt13)* deletion allele is a 2083-bp deletion that spans the second exon, which includes the *nlp-99* peptide region, and is hence predicted to result in a molecular null allele. It was generated via a *dpy-10*–based co-CRISPR strategy (table S3). We injected young adults with a mix consisting of 0.25 μl of Cas9 enzyme (15 mg/μl, Thermo Fisher Scientific), 0.26 μl of trans-activating CRISPR RNA [0.17 M, Integrated DNA Technologies (IDT)], 0.24 μl of *dpy-10* CRISPR RNA (crRNA; 0.6 nmol/μl, IDT), 0.20 μl of *nlp-99* crRNA1 (0.6 nmol/μl, IDT), 0.20 μl of *nlp-99* crRNA2 (0.6 nmol/μl, IDT), 2.2 μl of *dpy-10* repair template (0.5 mg/ml, Merck), and 1.1 μl of *nlp-99* repair template (1 mg/ml, IDT). Afterward, we screened the progeny for the desired gene edit via polymerase chain reaction. We verified the *ibt13* allele via sequencing (data S1).

To create a transcriptional fusion of NLP-99, we created a design in which the SL2 sequence of *gpd-2* and mKate2 sequence (codon optimized to CAI 1.0 and carrying two artificial introns) (*78*) were inserted after the stop codon of *nlp-99*. The allele *nlp-99(syb4792[nlp-99::SL2::mKate2])* carrying this design was generated by SunyBiotech (table S3 and data S1).

To create a translational fusion of NLP-99, we created a design in which a codon-optimized sequence encoding a linker (GSGSG) and an mKate2 sequence (codon optimized to CAI1.0 and carrying two artificial introns) (*78*) were inserted in the open reading frame before the stop codon of *nlp-99*. The allele *nlp-99(syb4879[nlp-99::linker::mKate2])* carrying this design was generated by SunyBiotech (table S3 and data S1).

To monitor FLP-11 secretion, we created a design with a codon-optimized (CAI1.0) (*78*) DNA sequence of a linker (GSGSGSGSG) and sfGFP before the stop codon of *flp-11*. The allele *flp-11(syb6321[flp-11::linker::sfGFP])* carrying this design was generated by SunyBiotech (table S3 and data S1).

For the expression of XCaMP-R (*45*) in RIS, we codon optimized XCaMP-R (CAI1.0) and inserted three introns (*78*) and had this sequence synthesized by Geneart/Thermo Fisher Scientific. We inserted this optimized version of XCaMP-R into a ski-lodge site *lgc-38(syb2346[flp-11p::dpy-10 site::flp-11 3′UTR] III:7007600)* that already contained the *flp-11* promoter and 3′UTR regions (*39*) to generate *lgc-38(syb2346goe13[flp-11p::XCaMP-R::flp-11 3′UTR]) III:7007600* (table S3 and data S1).

To express ReaChR in RIS, we used a previously generated transgenic allele in which ReaChR is driven by a 2834-bp *flp-11* promoter: *lgc-38(syb2346syb2493[flp-11-5′UTR::ReaChR-linker-mKate2::flp-11b-3′UTR]) III:7007600* (PHX2493) (*39*). The 2214 bp of this promoter was deleted by SunyBiotech according to our design, retaining the 620-bp region immediately upstream of the *flp-11* ATG, which includes the APTF-1 binding site (*32*). This generated the transgene *lgc-38(syb2346syb2493syb8234[flp-11-5′UTR(620bp)::ReaChR-linker-mKate2::flp-11b-3′UTR]) III:7007600* (PHX8234). The expression pattern of *lgc-38(syb2346syb2493syb8234)* closely resembled that of the original allele, with strong expression in RIS and weaker expression in additional cells. Optogenetic stimulation using this construct effectively activated RIS and induced behavioral quiescence, indicating that the 620-bp promoter fragment is sufficient and well suited for transgenic expression in RIS. The sequences can be found in table S3 and data S1.

For the expression of NLP-99 in RIS, we used the codon-optimized version of the *nlp-99* gene with a CAI of 1.0 (*78*) together with *SL2* from *gpd-2* from pUC57-Mini-ttx-3p::nlp-99::SL2::mKate2::unc-54-3′UTR. *nlp-99(CAI1.0)::SL2(gpd-2)* was inserted by SunyBiotech according to our design into PHX8234 *lgc-38(syb2346syb2493syb8234[flp-11-5′utr::ReaChR::linker::mKate2::flp-11b-3′utr] III:7007600)* after deletion of the sequences encoding the “ReaChR::linker.” This generated PHX10545 *lgc-38(syb2346syb2496syb8234syb10545[flp-11-5′utr::nlp-99(CAI1.0)::SL2(gpd-2)::mKate2::flp-11b-3′utr] III:7007600).* The sequences can be found in table S3 and data S1.

### Agarose hydrogel microchamber generation

We used agarose hydrogel microfluidic chambers to culture and simultaneously image sleep behavior and RIS activity (*8*, *38*) using either the GCaMP or XCaMP-R calcium indicator specifically expressed in RIS (*41*, *42*). For creating starvation conditions, we prepared the microchamber gel medium from 5% agarose dissolved in M9 buffer. For creating well-fed conditions, we used a 1:1 mix consisting of one part 10% agarose dissolved in M9 buffer and the other part of NGM.

To create the agarose chips, we first treated polydimethylsiloxane molds with air-plasma, melted the agarose at 95°C, and then cast the microchambers. We used chips containing many arrayed chambers sized 110 by 110 by 10 μm for L1 arrest, 370 by 370 by 10 μm for experiments presented in Fig. 1I, and 700 by 700 by 45 μm for adults.

We filled each individual microchamber with using a platinum wire pick. To induce L1 larval arrest, we placed pretzel-stage eggs into the chambers and kept them for 48 hours of starvation before imaging.

For starved adults, we placed young adult animals without bacterial food into the chambers and kept them for 12 hours before imaging. For well-fed adults, we applied floxuridine (FUdR) at a concentration of 50 μM. We placed 8 to 10 young L4 hermaphrodites onto the FUdR plates and, the next day, used them to prepare the fed adult chambers. We placed young adult worms together with OP50 as food into the chambers. We maintained the experimental plates at 20°C and imaged the chambers after 24 hours.

For imaging starvation conditions, we arranged animals of different genotypes (experimental mutants and their controls) on the same agarose chip and imaged them in parallel. Typically, for one experiment, we imaged two to four chips, with one agarose chip representing one biological replicate. We closed the microchambers with a coverslip, gluing it into an opening cut into a 3.5-cm petri dish. We filled the uncovered bottom area of the dish with agarose. If air bubbles appeared inside the chambers, we added 5 to 10 μl of M9 buffer on top of the agarose chip. We covered the dishes with their lids, wrapped them in Parafilm, and stored them upside down in an incubator at 20°C for 48 hours before imaging L1 arrest. To maintain the temperature and prevent condensation on the lid during imaging on inverted microscopes, we covered the dish with a heating lid set to 25.5°C, while the room temperature during imaging was ~19°C, resulting in an agarose chip temperature of around 20°C. For fed adults, we set the heating lid temperature to 25°C.

### DIC imaging and sleep detection via frame subtraction

To image the *npr-16* and *nlp-99* deletion mutants in differential interference contrast (DIC), we used a Nikon Eclipse Ti2 microscope equipped with an Andor Sona scientific complementary metal-oxide semiconductor (sCMOS; 4.2B-11) camera, with a 10× objective (CFI Plan Apo 10×, Nikon) and an additional 1.5× magnification lens. We filtered the diascopic light-emitting diode (LED) light with an infrared filter (785/62 Brightline HC, Semrock). For imaging L1 arrest, we acquired images at a rate of 0.2 Hz, setting exposure times between 40 and 50 ms for a period of 3 to 5 hours. We applied 2 × 2 binning to reduce the file size.

To image the rescue strain expressing *nlp-99* in RIS, we used a Nikon TiE inverted microscope equipped with an automated XY stage (Nikon), a 100-W halogen lamp (Osram), and a digital DS-Qi2 camera (SLR, FX-format CMOS sensor, Nikon) for long-term DIC imaging. We equipped the TiE microscope with an infrared filter (Semrock Brightline HC 785/62) and used a 10× objective lens to maximize the number of worms per field of view. After a 48-hour starvation period, we imaged the worms at a frame rate of 0.2 frames per second using 2 × 2 binning. We maintained constant illumination from the halogen lamp and set the exposure time to 40 ms.

We quantified the fraction of time spent sleeping by using frame subtraction to detect changes in movement across consecutive images. We distinguished worm pixels from background pixels using an intensity threshold between 180 and 200, which allowed us to accurately differentiate the worm body from the surrounding environment. To quantify movement, we calculated the percentage of the overlapping region between consecutive frames, providing a measure of movement by detecting shifts in pixel positions. We smoothed the percentage of overlap using a 20-frame moving average, which enhanced the accuracy of sleep state detection by excluding twitching movements during sleep. We defined sleep as periods when the worm maintained an overlap of more than 70% between consecutive images, indicating minimal to no movement, for at least 1 min.

### Calcium indicator imaging

For calcium indicator imaging during L1 arrest, we used a Nikon Eclipse Ti2 microscope equipped with either an Andor Sona back-illuminated (SCMOS 4.2B-11) camera or a Photometrics back-illuminated sCMOS (Prime 95B) camera. We used a 20× objective (Plan Apo 20×/0.75 DIC N2 WD1.0, Nikon) for arrested L1 animals and a 10× objective (CFI Plan Apo 10×, Nikon) for adult animals, typically using an additional 1.5× magnification lens. We supplied fluorescent excitation light with either a CoolLED pE-300-ultra (CoolLED) delivering 460- and 550-nm wavelengths, filtered through a standard LED-DA/FI/TX-3X-B-OMF filter set (Semrock), or a SOLA-SE-II (Lumencor) providing white light with a customized filter set (ET402/15 x T495lpxr ET525/50m, Chroma). We triggered the excitation light during exposure using transistor-transistor logic signals from the camera. We conducted all long-term imaging experiments with NIS Elements 5 software (Nikon), acquiring images at a frame rate of 0.2 Hz and setting exposure times to 40 to 60 ms. We applied 2 × 2 software binning to reduce file size.

For imaging fed adults, we used a Nikon Eclipse Ti2 microscope equipped with an Andor Sona sCMOS camera. We recorded movies at 10× magnification for 5 hours with a 15-s time interval and set the exposure time to 100 ms. Using the CoolLED pE-300-ultra, we illuminated the chamber with 460-nm light at 0.0250 mW/mm^2^ and 550-nm light at 0.0156 mW/mm^2^. We used a triple-band filter (LF405/488/594-3X-B-ZHE, Semrock) and applied no pixel binning.

We extracted calcium indicator intensities (GCaMP or XCaMP-R) to monitor the neuronal activity of RIS or AIY neurons (*8*, *40*). We identified the position of neurons by tracking DsRed, mKate2, or XCaMP-R. We calculated calcium indicator intensity by averaging the 10 highest pixel values in a 10 × 10 pixel square surrounding the target neuron. Then, we smoothed the averaged intensities over 30 frames. We also extracted and smoothed the intensities of mKate2 and DsRed over 30 frames to normalize the GCaMP intensity, obtaining ∆*R*/*R*. To calculate ∆*F*/*F*, we obtained baseline signals from individual calcium transients, identifying the lowest intensity within 10 min before sleep onset for sleep onset alignment or the highest RIS peak for RIS activity peak alignment. We defined sleep on the basis of the movement speed of either RIS or AIY neurons, determined by tracking the positions of DsRed, mKate2, or XCaMP-R. We defined sleep as periods when the worm maintained a movement speed of less than 1 μm per second for at least 1 min. We then aligned neuronal calcium activity with the corresponding fraction of time spent sleeping.

To extract GCaMP intensity specifically in the process of RIS, we used NIS artificial intelligence (AI) software (NIS AR 5.42.06). We trained the AI on fluorescence imaging movies of 59 worms expressing GCaMP and mKate2 in RIS. For each worm, we manually defined the RIS process in the mKate2 channel for the first three frames using the NIS Binary toolbar. We then used these manually defined regions as templates to train the program over 500 iterations using the Train Segment.ai function, generating the final training file for segmentation. Using these training data, we segmented all frames of all movies with the Segment.ai command. After identifying the positions of the processes across each movie, we extracted the mean intensities for each worm and frame using square regions of interest (ROIs) in NIS. Last, we exported the results to an Excel sheet for further analysis.

### *flp-11::sfGFP* reporter imaging

We performed *flp-11::sfGFP* reporter imaging on two developmental stages of *C. elegans* (L1 arrest and fed adult) using spinning disk confocal microscopy. To generate L1 arrest animals, we synchronized populations by bleaching as previously described (*36*) and resuspended the embryos in 1 ml of M9 buffer. We incubated the tubes on a rotator at 20 rpm and 20°C for 48 hours.

To obtain fed adults, we transferred a synchronized population of L4-stage worms onto fresh NGM plates seeded with OP50 bacteria 48 hours before imaging, ensuring developmental homogeneity. For imaging, we immobilized worms on 5% agarose pads (Thermo Fisher Scientific, BP164-500) prepared in M9 buffer. We poured 650 μl of liquid agarose onto a microscope slide, gently pressed it with a second slide for 10 to 30 s, and cut the solidified pad into a square patch. To immobilize the worms, we pipetted 5 μl of 50 mM chilled (~5°C) levamisole onto the pad and added 0.5 μl of L1 arrest worms or 10 to 12 adult worms, respectively.

We imaged the samples using a spinning disk confocal system mounted on a Nikon Ti2 inverted microscope equipped with an automated XY stage, a digital CMOS camera (2304 by 2304 pixels; ORCA-Fusion BT C15440, Hamamatsu), and a CSU-W1 confocal scanner unit (Yokogawa). We used a 60× oil immersion objective and illuminated the samples with a 488-nm laser (0.16 mW/mm^2^, 100-ms exposure) and standard GFP filters to detect sfGFP signals. For mKate2 signals, we used a 561-nm laser (50-ms exposure) and standard Texas Red filters.

We acquired *Z*-stacks at 0.5-μm intervals for L1 arrest worms and 1-μm intervals for adult worms. To analyze *flp-11::sfGFP* reporter expression, we generated maximum-intensity projections of the *Z*-stacks. Using MATLAB, we defined a rectangular ROI over the nerve ring region of the RIS neuron and extracted mean fluorescence intensity values for each worm. We imaged *flp-11::sfGFP* and *flp-11p::mKate2* reporter signals in two separate strains and normalized the sfGFP reporter expression signal of each individual to the averaged mKate2 reporter signal.

### Optogenetic experiments

To optogenetically activate AIY neurons, we expressed Chop-2(H134R), fused with tagRFP for visualization, from a *ttx-3* promoter (*sraEx281*) (*54*). Two hours before image acquisition, we supplemented the agarose chips containing the microchambers with either 10 μl of ethanol as a control or 10 μl of 10 mM all-trans-retinal (ATR; Sigma-Aldrich) dissolved in ethanol. We imaged two different strains (HBR3113 and HBR3114 or HBR3278) on each agarose chip. We tested two biological replicates for the control group (ethanol) and three biological replicates for the experimental group (ATR).

We used a Nikon Eclipse Ti2 microscope equipped with an Andor Sona back-illuminated sCMOS camera, featuring a 20× objective lens and an additional 1.5× magnification lens. Our imaging protocol comprised three phases, each lasting 3 min. First, we recorded baseline conditions before applying the light stimulus. Next, we applied the stimulus and then recorded the recovery phase. We imaged the animals throughout this entire protocol using infrared DIC (785/62 Brightline HC, Semrock) imaging at a frame rate of 0.33 Hz and an exposure time of 60 ms. We applied 4 × 4 binning to reduce file size. We achieved optogenetic stimulation by applying 460-nm light pulses filtered through a standard GFP filter set (Semrock), with an intensity of 0.57 mW/mm^2^. The computer software triggered the pulses. The agarose chips contained multiple sets of animals that were probed consecutively. After one set of animals had been assayed, 1 min of resting time was followed without imaging, and then the next set of individuals of the chip was assayed. The imaging protocol was repeated three times for each individual to produce three technical replicates. These three technical replicates were combined to form one *n*.

For the RIS optogenetic activation experiment combined with AIY calcium imaging, we cultured L1 larvae without food inside agarose microchambers as previously described (110 by 110 by 15 μm) and imaged them after 24 hours of starvation. We used a Nikon Ti2 microscope and NIS software (Nikon) for imaging.

We recorded GCaMP images for 3 min at a frame rate of 0.33 Hz using white light from a Sola Lumencor (0.13 mW/mm^2^) and a standard GFP filter set. Between GCaMP frames, we triggered a 1-s pulse of light (0.42 mW/mm^2^) through a Texas Red filter set. After the initial recording, we supplemented each microchamber with 10 μl of 10 mM ATR (Sigma-Aldrich) dissolved in ethanol to enable optogenetic activation. We repeated the same imaging protocol after 2 hours. For data analysis, we used a previously published MATLAB script (*39*). We applied a 30-frame smoothing filter to both the speed and fluorescence data.

To characterize the calcium response in RIS upon optogenetic activation using *lgc-38(syb2346syb2493syb8234[flp-11-5′UTR(620bp)::ReaChR-linker-mKate2::flp-11b-3′UTR])*, we cultured worms inside agarose microchambers and imaged them after 48 hours of starvation. We added 10 μl of ethanol to the control chamber without retinal and pipetted 10 μl of 10 mM ATR (Sigma-Aldrich) dissolved in ethanol onto the chambers for optogenetic activation. We imaged on a Ti2 microscope using a 20× objective and a triple-band pass filter (LF405/488/594-3X-B-ZHE, Semrock). We acquired images at a frame rate of 0.33 Hz using a 460-nm LED (0.14 mW/mm^2^) with a 50-ms exposure time for GCaMP imaging. First, we recorded 3 min of GCaMP baseline activity. To optogenetically stimulate RIS, we exposed worms to 550-nm LED light (0.12 mW/mm^2^) for 800 ms after each GCaMP frame during a 3-min stimulation phase. Last, we recorded a 3-min recovery phase with only GCaMP imaging. We coordinated the camera’s exposure and LED pulses using transistor-transistor logic triggering. For data analysis, we used a previously published MATLAB script (*39*). We applied a 30-frame smoothing filter to both the speed and fluorescence data.

### Fluorescence imaging and analysis of the *nlp-99* translational reporter

To prepare samples for the *nlp-99* translational reporter, we placed ~3 μl of worm suspension onto a drop of 25 mM levamisole on a 5% agarose pad for imaging. We performed fluorescence imaging using a Nikon TiE microscope equipped with an Andor Revolution spinning disk system (Andor Technology Ltd.), a CSU-X1 spinning disk head (Yokogawa), and an iXon Ultra camera (Andor Technology Ltd.). We used 488- and 561-nm lasers along with standard GFP and Texas Red filter sets. *Z*-stacks were acquired to span the entire worm.

To extract the signal from coelomocytes, we applied a lower intensity threshold in the GFP channel to generate a mask of pixels above this threshold, which defined coelomocyte locations. We then applied this mask to the red channel containing the *nlp-99* translational reporter (mKate2 signal). For quantifying NLP-99::mKate2 expression around the pharynx, we captured a single image in the red channel. We manually defined a ROI around the pharynx and selected the top 1000 pixels above a defined lower intensity threshold as the signal. We then applied background correction and calculated the mean intensity.

### Nile Red staining

For lipid staining using Nile Red (*62*), we synchronized animals by bleaching, as previously described (*36*). For L1 arrest measurements, we starved the larvae for 48 hours at 20°C with 20-rpm rotation. For adult measurements, we placed larvae synchronized by 48 hours of starvation onto seeded NGM plates and collected them for staining 6 days after release from arrest. We washed the animals off the plate with 1 ml of phosphate-buffered saline containing 0.01% Triton X-100, transferred them into a 1.5-ml tube, and pelleted the L1 worms by centrifugation at 4800 rpm for 1 min and the adults by letting them settle for ~3 min and carefully removed the supernatant. We fixed the worms in 100 μl of 40% (v/v) isopropanol at room temperature for 3 min. We centrifuged the worms at 4800 rpm for 1 min and carefully removed the supernatant. We then incubated the fixed animals in 500 μl of Nile Red working solution in the dark at 20°C with 20-rpm rotation for 1 hour. After incubation, we washed the animals with 600 μl of phosphate-buffered saline containing 0.01% Triton X-100 for 30 min, recollected them via centrifugation at 4800 rpm for 1 min, and then mounted them on a coverslip for fluorescence intensity measurement. We imaged the adult worms using a Nikon Eclipse Ti2 microscope equipped with an Andor Sona sCMOS (4.2B-11) camera and a 10× objective (CFI Plan Apo 10×, Nikon). We used a 460-nm LED at 20% intensity (0.04 mW/mm^2^) and a 550-nm LED at 20% intensity (0.07 mW/mm^2^) for excitation. We operated the camera with an electron-multiplying gain of 200 and a readout speed of 100 MHz and set the exposure time to 60 ms. We also captured DIC images.

### DHS-3::GFP lipid reporter imaging

For imaging the DHS-3::GFP lipid reporter, we synchronized the animals by bleaching them, following previously described methods (*36*). After bleaching, we resuspended the eggs in 1 ml of M9 buffer. We placed the sample tubes on a rotator set to 20 rpm and incubated them at 20°C. After 48 hours, we imaged the animals using spinning disk confocal microscopy. To prepare the worms for imaging, we immobilized them on a microscope slide using a thin layer of 5% agarose (Thermo Fisher Scientific, BP164-500) dissolved in M9 buffer. We created the agarose pad by applying 500 μl of liquid agarose (95°C) and pressing it with a second microscope slide for 10 to 30 s. To immobilize the worms, we pipetted 4 to 5 μl of 50 mM levamisole onto the agarose pad and spread the drop by tilting the slide. We then pipetted 0.5 μl of M9 solution containing the L1 arrest larvae into the levamisole. We imaged the worms using a spinning disk system mounted on a Nikon Ti2 inverted microscope, equipped with an automated XY stage, a digital CMOS camera (2304 by 2304 pixels, ORCA-Fusion BT C15440, Hamamatsu), and a CSU-W1 Confocal Scanner Unit (Yokogawa). We used a 100× oil immersion objective and illuminated the samples with a 488-nm laser (0.16 mW/mm^2^) using a 525/50-nm single-band pass emission filter (Semrock). We set the exposure time to 100 ms and acquired *Z*-stacks at 0.05-μm intervals. To analyze DHS-3 marker expression, we generated maximum intensity projections of the acquired *Z*-stacks. Using R, we applied background correction by subtracting the median pixel intensity from each pixel in the projection. Because each image contained a single worm, we used the median intensity as a representative background fluorescence. We then applied a median filter and a white top-hat transformation using a disc-shaped structuring element. Afterward, we applied a binary threshold to segment ROIs. We selected the radii for the median filter and top-hat structuring element, as well as the threshold value, to effectively isolate DHS-3–marked lipid droplets from diffuse marker expression. From each processed image, we extracted the total area of segmented features as a measure of lipid droplet abundance.

### Survival and lifespan assays

We synchronized animals by bleaching, as previously described (*36*). After bleaching, we resuspended the eggs in 1 ml of M9 buffer containing nystatin (10 units/ml; Sigma-Aldrich, N1638). We placed the sample tubes on a rotator set to 20 rpm and incubated them at 20°C. We designated the day of bleaching as day 0. For the survival assays during L1 arrest, we removed 10 to 15 μl of the suspended animal population from the sample and scored every animal as either alive or dead. We then calculated the percentage of live worms at that time point. For the adult lifespan assay, we cultured synchronized animals on NGM plates seeded with OP50 at 20°C, allowing them to develop into the adult stage. We set the first day of adulthood as day 0. We transferred a group of 10 to 15 young adult animals to NGM plates containing nystatin (100 units/ml) and 50 μM FUdR using OP50 as the food source. We scored lifespan every 2 or 3 days until all animals had died, noting any animals that died because of drying on the plate walls or were lost as censored. Data were fitted as described previously (*39*).

### Olfactory learning and memory assays

We carried out olfactory learning and memory assays using the chemotaxis of adult animals to DA, as described previously (*67*). We tested the attraction of well-fed, synchronized adult worms to the olfactory cue DA (2,3-butanedione). To measure the chemotaxis of the different strains, we washed the animals three times for 5 min with CTX (chemotaxis) buffer (5 mM KH_2_PO_4_/K_2_HPO_4_, pH 6.0, 1 mM CaCl_2_, and 1 mM MgSO_4_) to eliminate bacteria and placed 50 to 200 worms in the center of a 10-cm CTX testing plate containing 5 mM KH_2_PO_4_/K_2_HPO_4_ (pH 6.0), 1 mM CaCl_2_, 1 mM MgSO_4_, and 2% agar. We gave the worms a choice for 1 hour between a spot of 0.1% DA diluted in ethanol with 1 μl of 20 mM sodium azide and a reference spot containing ethanol and sodium azide. After 1 hour, we counted the worms within a 1-cm radius of either the attractant or reference spot, as well as the total number of worms on the plate. We visualized worm distribution using the chemotaxis index [Chemotaxis index = (Worms at compound spot − Worms at reference spot)/Total number of worms on plate]. We computed the memory index as Memory index = (CI_naive − CI_xh delay)/CI_naive.

To assess STM (*79*), we washed the well-fed young adult worms three times for 5 min with CTX buffer (5 mM KH_2_PO_4_/K_2_HPO_4_, pH 6.0, 1 mM CaCl_2_, and 1 mM MgSO_4_) to eliminate bacteria and either tested their attraction to DA before the exposure (naive) or exposed the animals to 1 hour of starvation in the presence of 2 μl of undiluted DA on 10-cm CTX plates (5 mM KH_2_PO_4_/K_2_HPO_4_, pH 6.0, 1 mM CaCl_2_, 1 mM MgSO_4_, and 2% agar). Following training, we tested attraction to DA immediately after the exposure (conditioned) and again following a 1-hour rest without DA with abundant food (1-hour delay).

For olfactory long-term associative memory conditioning (*80*), we exposed well-fed young adult worms to two 1-hour starvation periods in the presence of 2 μl of undiluted DA, with a 30-min rest interval keeping the animals on food, between the two training rounds. After the spaced training, we transferred the worms to NGM plates with abundant food for a 24-hour recovery period and then tested their chemotaxis. For each experiment, we tested a subset of the population before training (naive), immediately after training (conditioned), and 24 hours after training (24-hour delay). For all experimental conditions, we used three independent test plates and repeated the assay at least three times on separate days.

### In vitro electrophysiology recordings

#### Plasmids and RNA preparation

To express NPR-16a in *X. laevis* oocytes, we inserted the receptor’s cDNA sequence into a KSM plasmid vector backbone containing *Xenopus* β-globin UTR regions and a T3 promoter using HiFi assembly (NEB). We also prepared mGIRK1 (NM_001355118.1) and mGIRK2 (NM_001025584.2), two mouse G protein inward rectifying potassium channels, along with the mouse muscarinic GPCR M2 (NM_001411688.1) from mouse cDNA. After linearizing the plasmids with Not I, we synthesized 5′ capped cRNA in vitro using the T3 mMessage mMachine transcription kit (Thermo Fisher Scientific, Waltham, MA). We then purified the resulting cRNA using the GeneJET RNA purification kit (Thermo Fisher Scientific).

#### Synthetic peptide synthesis

NLP-99 peptide stocks (GDGYGWNDCEFSPLSCLL, C9-C16 disulfide bridge) were synthesized by GL Biochem Ltd. at >95% purity, reconstituted in 80% acetonitrile, and stored at −20°C.

#### TEVC recording

Two days before the TEVC recording, we placed defolliculated *Xenopus* oocytes (EcoCyte Bioscience) individually into V-bottom 96-well plates and injected them with 50 nl of an RNA mix containing NPR-16a (200 ng/μl), mGIRK1 (150 ng/μl), and mGIRK2 (150 ng/μl) using the automated Roboinject system (Multi Channel Systems GmbH). We kept the injected oocytes at 16°C in ND96.

We performed recordings using the Robocyte2 system equipped with electrodes characterized by a resistance of 0.7 to 2 MΩ (Multi Channel Systems). We filled the system’s pipettes with 1.5 M KCl and 1 M acetic acid, and we clamped the oocytes at −80 mV. We conducted continuous recordings at 500 Hz, collecting data with the manufacturer’s Robocyte2 control software. Each recording lasted for 80 s and was subdivided into four phases. First, we perfused the oocytes for 10 s with ND96 to determine each oocyte’s resting potential, followed by 20 s of high-K^+^ solution (96 mM KCl, 1 mM MgCl_2_, 5 mM Hepes, 1.8 mM CaCl_2_, and 2 mM NaCl) to open the GIRK channels and estimate their baseline current. Next, we applied NLP-99 peptide (1 μM in high-K^+^ solution) to the oocytes for 20 s, followed by a 30-s perfusion with ND96 to regain the resting potential. To define the G protein coupling of NPR-16, we injected half of the oocytes with 50 nl of PTX (100 ng/μl; Gibco) 6 to 7 hours before recording. We used a known G_i/o_-coupled GPCR, M2, as a positive control (*28*). To extract the data, we used Robocyte2^+^ analysis software. We calculated the “% GIRK activation” as the ratio between the baseline high-K^+^ current and the NLP-99/high-K^+^ current.

### Statistical analysis and replicates

To account for variability in the fraction of time spent sleeping across experiments, internal controls were included in all cases, and statistical comparisons were made only between groups within the same internal controls. Statistical analyses are described in the corresponding Materials and Methods sections and figure legends. “*n*” refers to the number of animals used, unless otherwise specified. All replicates are biological.

### Use of AI in writing the manuscript text

During the preparation of this work, the authors used ChatGPT (GPT-3.5, GPT-4, and GPT-4 Turbo) and you.com (GPT-4, Claude Instant, and Gemini Pro) for parts of the text written by the authors to assess its clarity and grammatical correctness. The tools were asked to evaluate whether specific sections were clear and grammatically correct. They then provided comments and suggestions for improvement. In some cases, these suggestions were adopted to enhance the text. Any changes made were carefully reviewed to ensure the original meaning was preserved. The authors take full responsibility for the content of the text.

## References

[1] P. Brazeau, W. Vale, R. Burgus, N. Ling, M. Butcher, J. Rivier, R. Guillemin, Hypothalamic polypeptide that inhibits the secretion of immunoreactive pituitary growth hormone. Science 179, 77–79 (1973). 4682131 10.1126/science.179.4068.77

[2] J. M. Adams, V. Otero-Corchon, G. L. Hammond, J. D. Veldhuis, N. Qi, M. J. Low, Somatostatin is essential for the sexual dimorphism of GH secretion, corticosteroid-binding globulin production, and corticosterone levels in mice. Endocrinology 156, 1052–1065 (2015). 25551181 10.1210/en.2014-1429 PMC4330306

[3] H. M. Brown-Borg, Hormonal control of aging in rodents: The somatotropic axis. Mol. Cell. Endocrinol. 299, 64–71 (2009). 18674587 10.1016/j.mce.2008.07.001 PMC4390024

[4] E. Van Cauter, G. Copinschi, Interrelationships between growth hormone and sleep. Growth Horm. IGF Res. 10 (Suppl. B), S57–S62 (2000). 10984255 10.1016/s1096-6374(00)80011-8

[5] M. H. Schmidt, The energy allocation function of sleep: A unifying theory of sleep, torpor, and continuous wakefulness. Neurosci. Biobehav. Rev. 47, 122–153 (2014). 25117535 10.1016/j.neubiorev.2014.08.001

[6] M. S. Kayser, D. Biron, Sleep and development in genetically tractable model organisms. Genetics 203, 21–33 (2016). 27183564 10.1534/genetics.116.189589 PMC4858775

[7] S. Diekelmann, J. Born, The memory function of sleep. Nat. Rev. Neurosci. 11, 114–126 (2010). 20046194 10.1038/nrn2762

[8] Y. Wu, F. Masurat, J. Preis, H. Bringmann, Sleep counteracts aging phenotypes to survive starvation-induced developmental arrest in *C. elegans*. Curr. Biol. 28, 3610–3624.e8 (2018). 30416057 10.1016/j.cub.2018.10.009 PMC6264389

[9] M. P. Sinner, F. Masurat, J. J. Ewbank, N. Pujol, H. Bringmann, Innate immunity promotes sleep through epidermal antimicrobial peptides. Curr. Biol. 31, 564–577.e12 (2021). 33259791 10.1016/j.cub.2020.10.076

[10] H. Bringmann, Sleep-active neurons: Conserved motors of sleep. Genetics 208, 1279–1289 (2018). 29618588 10.1534/genetics.117.300521 PMC5887131

[11] C. B. Saper, P. M. Fuller, N. P. Pedersen, J. Lu, T. E. Scammell, Sleep state switching. Neuron 68, 1023–1042 (2010). 21172606 10.1016/j.neuron.2010.11.032 PMC3026325

[12] X. Yu, G. Zhao, D. Wang, S. Wang, R. Li, A. Li, H. Wang, M. Nollet, Y. Y. Chun, T. Zhao, R. Yustos, H. Li, J. Zhao, J. Li, M. Cai, A. L. Vyssotski, Y. Li, H. Dong, N. P. Franks, W. Wisden, A specific circuit in the midbrain detects stress and induces restorative sleep. Science 377, 63–72 (2022). 35771921 10.1126/science.abn0853 PMC7612951

[13] M. Xu, S. Chung, S. Zhang, P. Zhong, C. Ma, W. C. Chang, B. Weissbourd, N. Sakai, L. Luo, S. Nishino, Y. Dan, Basal forebrain circuit for sleep-wake control. Nat. Neurosci. 18, 1641–1647 (2015). 26457552 10.1038/nn.4143 PMC5776144

[14] N. Niethard, H. V. Ngo, I. Ehrlich, J. Born, Cortical circuit activity underlying sleep slow oscillations and spindles. Proc. Natl. Acad. Sci. U.S.A. 115, E9220–E9229 (2018). 30209214 10.1073/pnas.1805517115 PMC6166829

[15] C. M. Funk, K. Peelman, M. Bellesi, W. Marshall, C. Cirelli, G. Tononi, Role of somatostatin-positive cortical interneurons in the generation of sleep slow waves. J. Neurosci. 37, 9132–9148 (2017). 28821651 10.1523/JNEUROSCI.1303-17.2017 PMC5607463

[16] K. Tossell, X. Yu, P. Giannos, B. Anuncibay Soto, M. Nollet, R. Yustos, G. Miracca, M. Vicente, A. Miao, B. Hsieh, Y. Ma, A. L. Vyssotski, T. Constandinou, N. P. Franks, W. Wisden, Somatostatin neurons in prefrontal cortex initiate sleep-preparatory behavior and sleep via the preoptic and lateral hypothalamus. Nat. Neurosci. 26, 1805–1819 (2023). 37735497 10.1038/s41593-023-01430-4 PMC10545541

[17] I. Hajdu, E. Szentirmai, F. Obal Jr., J. M. Krueger, Different brain structures mediate drinking and sleep suppression elicited by the somatostatin analog, octreotide, in rats. Brain Res. 994, 115–123 (2003). 14642455 10.1016/j.brainres.2003.09.029

[18] M. Ziegenbein, K. Held, H. E. Kuenzel, H. Murck, I. A. Antonijevic, A. Steiger, The somatostatin analogue octreotide impairs sleep and decreases EEG sigma power in young male subjects. Neuropsychopharmacology 29, 146–151 (2004). 12955096 10.1038/sj.npp.1300298

[19] C. Anaclet, R. De Luca, A. Venner, O. Malyshevskaya, M. Lazarus, E. Arrigoni, P. M. Fuller, Genetic activation, inactivation, and deletion reveal a limited and nuanced role for somatostatin-containing basal forebrain neurons in behavioral state control. J. Neurosci. 38, 5168–5181 (2018). 29735555 10.1523/JNEUROSCI.2955-17.2018 PMC5977448

[20] J. Delorme, L. Wang, F. R. Kuhn, V. Kodoth, J. Ma, J. D. Martinez, F. Raven, B. A. Toth, V. Balendran, A. Vega Medina, S. Jiang, S. J. Aton, Sleep loss drives acetylcholine- and somatostatin interneuron-mediated gating of hippocampal activity to inhibit memory consolidation. Proc. Natl. Acad. Sci. U.S.A. 118, e2019318118 (2021). 34344824 10.1073/pnas.2019318118 PMC8364159

[21] K. A. Cummings, R. L. Clem, Prefrontal somatostatin interneurons encode fear memory. Nat. Neurosci. 23, 61–74 (2020). 31844314 10.1038/s41593-019-0552-7 PMC6930333

[22] V. Sharma, R. Sood, A. Khlaifia, M. J. Eslamizade, T. Y. Hung, D. Lou, A. Asgarihafshejani, M. Lalzar, S. J. Kiniry, M. P. Stokes, N. Cohen, A. J. Nelson, K. Abell, A. P. Possemato, S. Gal-Ben-Ari, V. T. Truong, P. Wang, A. Yiannakas, F. Saffarzadeh, A. C. Cuello, K. Nader, R. J. Kaufman, M. Costa-Mattioli, P. V. Baranov, A. Quintana, E. Sanz, A. Khoutorsky, J. C. Lacaille, K. Rosenblum, N. Sonenberg, eIF2alpha controls memory consolidation via excitatory and somatostatin neurons. Nature 586, 412–416 (2020). 33029011 10.1038/s41586-020-2805-8 PMC7874887

[23] C. Kluge, C. Stoppel, C. Szinyei, O. Stork, H. C. Pape, Role of the somatostatin system in contextual fear memory and hippocampal synaptic plasticity. Learn. Mem. 15, 252–260 (2008). 18391186 10.1101/lm.793008 PMC2327267

[24] P. Barnett, Somatostatin and somatostatin receptor physiology. Endocrine 20, 255–264 (2003). 12721505 10.1385/ENDO:20:3:255

[25] J. A. Veenstra, Allatostatin C and its paralog allatostatin double C: The arthropod somatostatins. Insect Biochem. Mol. Biol. 39, 161–170 (2009). 19063967 10.1016/j.ibmb.2008.10.014

[26] I. Titos, A. Juginovic, A. Vaccaro, K. Nambara, P. Gorelik, O. Mazor, D. Rogulja, A gut-secreted peptide suppresses arousability from sleep. Cell 186, 2273–2274 (2023). 37172567 10.1016/j.cell.2023.04.005 PMC10479874

[27] O. Kubrak, T. Koyama, N. Ahrentlov, L. Jensen, A. Malita, M. T. Naseem, M. Lassen, S. Nagy, M. J. Texada, K. V. Halberg, K. Rewitz, The gut hormone Allatostatin C/Somatostatin regulates food intake and metabolic homeostasis under nutrient stress. Nat. Commun. 13, 692 (2022). 35121731 10.1038/s41467-022-28268-x PMC8816919

[28] I. Beets, S. Zels, E. Vandewyer, J. Demeulemeester, J. Caers, E. Baytemur, A. Courtney, L. Golinelli, I. Hasakiogullari, W. R. Schafer, P. E. Vertes, O. Mirabeau, L. Schoofs, System-wide mapping of peptide-GPCR interactions in *C. elegans*. Cell Rep. 42, 113058 (2023). 37656621 10.1016/j.celrep.2023.113058 PMC7615250

[29] L. Golinelli, E. Geens, A. Irvine, C. J. McCoy, E. Vandewyer, L. E. Atkinson, A. Mousley, L. Temmerman, I. Beets, Global analysis of neuropeptide receptor conservation across phylum Nematoda. BMC Biol. 22, 223 (2024). 39379997 10.1186/s12915-024-02017-6 PMC11462694

[30] B. Cockx, S. Van Bael, R. Boelen, E. Vandewyer, H. Yang, T. A. Le, J. J. Dalzell, I. Beets, C. Ludwig, J. Lee, L. Temmerman, Mass spectrometry-driven discovery of neuropeptides mediating nictation behavior of nematodes. Mol. Cell. Proteomics 22, 100479 (2023). 36481452 10.1016/j.mcpro.2022.100479 PMC9881375

[31] M. Turek, I. Lewandrowski, H. Bringmann, An AP2 transcription factor is required for a sleep-active neuron to induce sleep-like quiescence in *C. elegans*. Curr. Biol. 23, 2215–2223 (2013). 24184105 10.1016/j.cub.2013.09.028

[32] M. Turek, J. Besseling, J. P. Spies, S. Konig, H. Bringmann, Sleep-active neuron specification and sleep induction require FLP-11 neuropeptides to systemically induce sleep. eLife 5, e12499 (2016). 26949257 10.7554/eLife.12499 PMC4805538

[33] L. Rossi, K. Amoako, I. Busack, L. Golinelli, A. Courtney, J. Besseling, W. Schafer, I. Beets, H. Bringmann, The neuropeptide FLP-11 induces and self-inhibits sleep through the receptor DMSR-1 in *Caenorhabditis elegans*. Curr. Biol. 35, 2183–2194.e10 (2025). 40273913 10.1016/j.cub.2025.03.039 PMC7617803

[34] D. M. Raizen, J. E. Zimmerman, M. H. Maycock, U. D. Ta, Y. J. You, M. V. Sundaram, A. I. Pack, Lethargus is a *Caenorhabditis elegans* sleep-like state. Nature 451, 569–572 (2008). 18185515 10.1038/nature06535

[35] A. J. Hill, R. Mansfield, J. M. Lopez, D. M. Raizen, C. Van Buskirk, Cellular stress induces a protective sleep-like state in *C. elegans*. Curr. Biol. 24, 2399–2405 (2014). 25264259 10.1016/j.cub.2014.08.040 PMC4254280

[36] J. Konietzka, M. Fritz, S. Spiri, R. McWhirter, A. Leha, S. Palumbos, W. S. Costa, A. Oranth, A. Gottschalk, D. M. Miller III, A. Hajnal, H. Bringmann, Epidermal growth factor signaling promotes sleep through a combined series and parallel neural circuit. Curr. Biol. 30, 1–16.e13 (2020). 31839447 10.1016/j.cub.2019.10.048

[37] S. Skora, F. Mende, M. Zimmer, Energy scarcity promotes a brain-wide sleep state modulated by insulin signaling in *C. elegans*. Cell Rep. 22, 953–966 (2018). 29386137 10.1016/j.celrep.2017.12.091 PMC5846868

[38] A. Koutsoumparis, L. M. Welp, A. Wulf, H. Urlaub, D. Meierhofer, S. Börno, B. Timmermann, I. Busack, H. Bringmann, Sleep neuron depolarization promotes protective gene expression changes and FOXO activation. Curr. Biol. 32, 2248–2262.e9 (2022). 35504281 10.1016/j.cub.2022.04.012

[39] I. Busack, H. Bringmann, A sleep-active neuron can promote survival while sleep behavior is disturbed. PLOS Genet. 19, e1010665 (2023). 36917595 10.1371/journal.pgen.1010665 PMC10038310

[40] E. Maluck, I. Busack, J. Besseling, F. Masurat, M. Turek, K. E. Busch, H. Bringmann, A wake-active locomotion circuit depolarizes a sleep-active neuron to switch on sleep. PLoS Biol. 18, e3000361 (2020). 32078631 10.1371/journal.pbio.3000361 PMC7053779

[41] H. Bringmann, Agarose hydrogel microcompartments for imaging sleep- and wake-like behavior and nervous system development in *Caenorhabditis elegans* larvae. J. Neurosci. Methods 201, 78–88 (2011). 21801751 10.1016/j.jneumeth.2011.07.013

[42] M. Turek, J. Besseling, H. Bringmann, Agarose microchambers for long-term calcium imaging of *Caenorhabditis elegans*. J. Vis. Exp., e52742 (2015). 26132740 10.3791/52742 PMC4544933

[43] W. Steuer Costa, P. Van der Auwera, C. Glock, J. F. Liewald, M. Bach, C. Schuler, S. Wabnig, A. Oranth, F. Masurat, H. Bringmann, L. Schoofs, E. H. K. Stelzer, S. C. Fischer, A. Gottschalk, A GABAergic and peptidergic sleep neuron as a locomotion stop neuron with compartmentalized Ca^2+^ dynamics. Nat. Commun. 10, 4095 (2019). 31506439 10.1038/s41467-019-12098-5 PMC6736843

[44] P. Laurent, Z. Soltesz, G. M. Nelson, C. Chen, F. Arellano-Carbajal, E. Levy, M. de Bono, Decoding a neural circuit controlling global animal state in *C. elegans*. eLife 4, e04241 (2015). 25760081 10.7554/eLife.04241 PMC4440410

[45] M. Inoue, A. Takeuchi, S. Manita, S. I. Horigane, M. Sakamoto, R. Kawakami, K. Yamaguchi, K. Otomo, H. Yokoyama, R. Kim, T. Yokoyama, S. Takemoto-Kimura, M. Abe, M. Okamura, Y. Kondo, S. Quirin, C. Ramakrishnan, T. Imamura, K. Sakimura, T. Nemoto, M. Kano, H. Fujii, K. Deisseroth, K. Kitamura, H. Bito, Rational engineering of XCaMPs, a multicolor GECI suite for in vivo imaging of complex brain circuit dynamics. Cell 177, 1346–1360.e24 (2019). 31080068 10.1016/j.cell.2019.04.007

[46] S. R. Taylor, G. Santpere, A. Weinreb, A. Barrett, M. B. Reilly, C. Xu, E. Varol, P. Oikonomou, L. Glenwinkel, R. McWhirter, A. Poff, M. Basavaraju, I. Rafi, E. Yemini, S. J. Cook, A. Abrams, B. Vidal, C. Cros, S. Tavazoie, N. Sestan, M. Hammarlund, O. Hobert, D. M. Miller III, Molecular topography of an entire nervous system. Cell 184, 4329–4347.e23 (2021). 34237253 10.1016/j.cell.2021.06.023 PMC8710130

[47] H. Kunitomo, H. Sato, R. Iwata, Y. Satoh, H. Ohno, K. Yamada, Y. Iino, Concentration memory-dependent synaptic plasticity of a taste circuit regulates salt concentration chemotaxis in *Caenorhabditis elegans*. Nat. Commun. 4, 2210 (2013). 23887678 10.1038/ncomms3210

[48] A. S. Wenick, O. Hobert, Genomic cis-regulatory architecture and trans-acting regulators of a single interneuron-specific gene battery in *C. elegans*. Dev. Cell 6, 757–770 (2004). 15177025 10.1016/j.devcel.2004.05.004

[49] J. M. Gray, J. J. Hill, C. I. Bargmann, A circuit for navigation in *Caenorhabditis elegans*. Proc. Natl. Acad. Sci. U.S.A. 102, 3184–3191 (2005). 15689400 10.1073/pnas.0409009101 PMC546636

[50] M. de Bono, A. V. Maricq, Neuronal substrates of complex behaviors in *C. elegans*. Annu. Rev. Neurosci. 28, 451–501 (2005). 16022603 10.1146/annurev.neuro.27.070203.144259

[51] M. J. Iannacone, I. Beets, L. E. Lopes, M. A. Churgin, C. Fang-Yen, M. D. Nelson, L. Schoofs, D. M. Raizen, The RFamide receptor DMSR-1 regulates stress-induced sleep in *C. elegans*. eLife 6, e19837 (2017). 28094002 10.7554/eLife.19837 PMC5241116

[52] A. Koutsoumparis, I. Busack, C. K. Chen, Y. Hayashi, B. P. Braeckman, D. Meierhofer, H. Bringmann, Reverse genetic screening during L1 arrest reveals a role of the diacylglycerol kinase 1 gene dgk-1 and sphingolipid metabolism genes in sleep regulation. Genetics 225, iyad124 (2023). 37682641 10.1093/genetics/iyad124

[53] Z. Li, J. Liu, M. Zheng, X. Z. Xu, Encoding of both analog- and digital-like behavioral outputs by one *C. elegans* interneuron. Cell 159, 751–765 (2014). 25417153 10.1016/j.cell.2014.09.056 PMC4243084

[54] A. Kocabas, C. H. Shen, Z. V. Guo, S. Ramanathan, Controlling interneuron activity in *Caenorhabditis elegans* to evoke chemotactic behaviour. Nature 490, 273–277 (2012). 23000898 10.1038/nature11431 PMC4229948

[55] M. Hirano, R. Ando, S. Shimozono, M. Sugiyama, N. Takeda, H. Kurokawa, R. Deguchi, K. Endo, K. Haga, R. Takai-Todaka, S. Inaura, Y. Matsumura, H. Hama, Y. Okada, T. Fujiwara, T. Morimoto, K. Katayama, A. Miyawaki, A highly photostable and bright green fluorescent protein. Nat. Biotechnol. 40, 1132–1142 (2022). 35468954 10.1038/s41587-022-01278-2 PMC9287174

[56] W. Steuer Costa, S. C. Yu, J. F. Liewald, A. Gottschalk, Fast cAMP modulation of neurotransmission via neuropeptide signals and vesicle loading. Curr. Biol. 27, 495–507 (2017). 28162892 10.1016/j.cub.2016.12.055

[57] C. Hatcher-Solis, M. Fribourg, K. Spyridaki, J. Younkin, A. Ellaithy, G. Xiang, G. Liapakis, J. Gonzalez-Maeso, H. Zhang, M. Cui, D. E. Logothetis, G protein-coupled receptor signaling to Kir channels in *Xenopus* oocytes. Curr. Pharm. Biotechnol. 15, 987–995 (2014). 25374032 10.2174/1389201015666141031111916 PMC4426293

[58] L. R. Baugh, P. W. Sternberg, DAF-16/FOXO regulates transcription of cki-1/Cip/Kip and repression of lin-4 during *C. elegans* L1 arrest. Curr. Biol. 16, 780–785 (2006). 16631585 10.1016/j.cub.2006.03.021

[59] E. Van Cauter, K. Spiegel, E. Tasali, R. Leproult, Metabolic consequences of sleep and sleep loss. Sleep Med. 9 (Suppl. 1), S23–S28 (2008). 18929315 10.1016/S1389-9457(08)70013-3 PMC4444051

[60] M. Mackiewicz, K. R. Shockley, M. A. Romer, R. J. Galante, J. E. Zimmerman, N. Naidoo, D. A. Baldwin, S. T. Jensen, G. A. Churchill, A. I. Pack, Macromolecule biosynthesis: A key function of sleep. Physiol. Genomics 31, 441–457 (2007). 17698924 10.1152/physiolgenomics.00275.2006

[61] J. J. Grubbs, L. E. Lopes, A. M. van der Linden, D. M. Raizen, A salt-induced kinase is required for the metabolic regulation of sleep. PLoS Biol. 18, e3000220 (2020). 32315298 10.1371/journal.pbio.3000220 PMC7173979

[62] N. L. Stuhr, J. D. Nhan, A. M. Hammerquist, B. Van Camp, D. Reoyo, S. P. Curran, Rapid lipid quantification in *Caenorhabditis elegans* by Oil Red O and Nile Red staining. Bio Protoc. 12, e4340 (2022). 10.21769/BioProtoc.4340 PMC8918222 35592599

[63] H. I. Ha, M. Hendricks, Y. Shen, C. V. Gabel, C. Fang-Yen, Y. Qin, D. Colon-Ramos, K. Shen, A. D. Samuel, Y. Zhang, Functional organization of a neural network for aversive olfactory learning in *Caenorhabditis elegans*. Neuron 68, 1173–1186 (2010). 21172617 10.1016/j.neuron.2010.11.025 PMC3038580

[64] X. Jin, N. Pokala, C. I. Bargmann, Distinct circuits for the formation and retrieval of an imprinted olfactory memory. Cell 164, 632–643 (2016). 26871629 10.1016/j.cell.2016.01.007 PMC5065712

[65] R. Chandra, F. Farah, F. Munoz-Lobato, A. Bokka, K. L. Benedetti, C. Brueggemann, M. F. A. Saifuddin, J. M. Miller, J. Li, E. Chang, A. Varshney, V. Jimenez, A. Baradwaj, C. Nassif, S. Alladin, K. Andersen, A. J. Garcia, V. Bi, S. K. Nordquist, R. L. Dunn, V. Garcia, K. Tokalenko, E. Soohoo, F. Briseno, S. Kaur, M. Harris, H. Guillen, D. Byrd, B. Fung, A. E. Bykov, E. Odisho, B. Tsujimoto, A. Tran, A. Duong, K. C. Daigle, R. Paisner, C. E. Zuazo, C. Lin, A. Asundi, M. A. Churgin, C. Fang-Yen, M. Bremer, S. Kato, M. K. VanHoven, N. D. L’Etoile, Sleep is required to consolidate odor memory and remodel olfactory synapses. Cell 186, 2911–2928.e20 (2023). 37269832 10.1016/j.cell.2023.05.006 PMC10354834

[66] M. Makino, E. Ulzii, R. Shirasaki, J. Kim, Y.-J. You, Regulation of satiety quiescence by neuropeptide signaling in *Caenorhabditis elegans*. Front. Neurosci. 15, 678590 (2021). 34335159 10.3389/fnins.2021.678590 PMC8319666

[67] B. G. Fenyves, A. Arnold, V. G. Gharat, C. Haab, K. Tishinov, F. Peter, D. de Quervain, A. Papassotiropoulos, A. Stetak, Dual role of an mps-2/KCNE-dependent pathway in long-term memory and age-dependent memory decline. Curr. Biol. 31, 527–539.e7 (2021). 33259792 10.1016/j.cub.2020.10.069

[68] A. L. A. Nichols, T. Eichler, R. Latham, M. Zimmer, A global brain state underlies *C. elegans* sleep behavior. Science 356, eaam6851 (2017). 28642382 10.1126/science.aam6851

[69] J. G. White, E. Southgate, J. N. Thomson, S. Brenner, The structure of the nervous system of the nematode *Caenorhabditis elegans*. Philos. Trans. R. Soc. Lond. B Biol. Sci. 314, 1–340 (1986). 22462104 10.1098/rstb.1986.0056

[70] S. J. Cook, T. A. Jarrell, C. A. Brittin, Y. Wang, A. E. Bloniarz, M. A. Yakovlev, K. C. Q. Nguyen, L. T. Tang, E. A. Bayer, J. S. Duerr, H. E. Bulow, O. Hobert, D. H. Hall, S. W. Emmons, Whole-animal connectomes of both *Caenorhabditis elegans* sexes. Nature 571, 63–71 (2019). 31270481 10.1038/s41586-019-1352-7 PMC6889226

[71] J. E. Sulston, E. Schierenberg, J. G. White, J. N. Thomson, The embryonic cell lineage of the nematode *Caenorhabditis elegans*. Dev. Biol. 100, 64–119 (1983). 6684600 10.1016/0012-1606(83)90201-4

[72] M. W. Moyle, K. M. Barnes, M. Kuchroo, A. Gonopolskiy, L. H. Duncan, T. Sengupta, L. Shao, M. Guo, A. Santella, R. Christensen, A. Kumar, Y. Wu, K. R. Moon, G. Wolf, S. Krishnaswamy, Z. Bao, H. Shroff, W. A. Mohler, D. A. Colon-Ramos, Structural and developmental principles of neuropil assembly in *C. elegans*. Nature 591, 99–104 (2021). 33627875 10.1038/s41586-020-03169-5 PMC8385650

[73] C. A. Brittin, S. J. Cook, D. H. Hall, S. W. Emmons, N. Cohen, A multi-scale brain map derived from whole-brain volumetric reconstructions. Nature 591, 105–110 (2021). 33627874 10.1038/s41586-021-03284-x PMC11648602

[74] L. Ripoll-Sanchez, J. Watteyne, H. Sun, R. Fernandez, S. R. Taylor, A. Weinreb, B. L. Bentley, M. Hammarlund, D. M. Miller III, O. Hobert, I. Beets, P. E. Vertes, W. R. Schafer, The neuropeptidergic connectome of *C. elegans*. Neuron 111, 3570–3589.e5 (2023). 37935195 10.1016/j.neuron.2023.09.043 PMC7615469

[75] F. Randi, A. K. Sharma, S. Dvali, A. M. Leifer, Neural signal propagation atlas of *Caenorhabditis elegans*. Nature 623, 406–414 (2023). 37914938 10.1038/s41586-023-06683-4 PMC10632145

[76] M. Gendrel, E. G. Atlas, O. Hobert, A cellular and regulatory map of the GABAergic nervous system of *C. elegans*. eLife 5, e17686 (2016). 27740909 10.7554/eLife.17686 PMC5065314

[77] A. Lukomska, G. Dobrzanski, M. Liguz-Lecznar, M. Kossut, Somatostatin receptors (SSTR1-5) on inhibitory interneurons in the barrel cortex. Brain Struct. Funct. 225, 387–401 (2020). 31873798 10.1007/s00429-019-02011-7 PMC6957562

[78] S. Redemann, S. Schloissnig, S. Ernst, A. Pozniakowsky, S. Ayloo, A. A. Hyman, H. Bringmann, Codon adaptation-based control of protein expression in *C. elegans*. Nat. Methods 8, 250–252 (2011). 21278743 10.1038/nmeth.1565

[79] W. M. Nuttley, K. P. Atkinson-Leadbeater, D. Van Der Kooy, Serotonin mediates food-odor associative learning in the nematode *Caenorhabditis elegans*. Proc. Natl. Acad. Sci. U.S.A. 99, 12449–12454 (2002). 12202746 10.1073/pnas.192101699 PMC129465

[80] V. Vukojevic, L. Gschwind, C. Vogler, P. Demougin, D. J. de Quervain, A. Papassotiropoulos, A. Stetak, A role for alpha-adducin (ADD-1) in nematode and human memory. EMBO J. 31, 1453–1466 (2012). 22307086 10.1038/emboj.2012.14 PMC3321180

