# Supplementary material for: C. elegans somatostatin/allatostatin C signaling regulates sleep, metabolism, survival, and memory via a sleep-active neuron
Source: Sci Adv. 2026 Apr 15;12(16):eadv8387. doi: 10.1126/sciadv.adv8387 (PMC13082328; doi:10.1126/sciadv.adv8387)
Supplement: Supplementary file 1 — Figs. S1 to S10 Legend for data S1 Legends for tables S1 to S3 [file sciadv.adv8387_sm.pdf]

Science Advances
AAAS

Supplementary Materials for

C. elegans somatostatin/allatostatin C signaling regulates sleep, metabolism, survival, and memory via a sleep-active neuron

Byoungjun Park *et al.*

Corresponding author: Henrik Bringmann, henrik.bringmann@tu-dresden.de

Sci. Adv. **12**, eadv8387 (2026)
DOI: 10.1126/sciadv.adv8387

The PDF file includes:

Figs. S1 to S10
Legend for data S1
Legends for tables S1 to S3

Other Supplementary Material for this manuscript includes the following:

Data S1
Tables S1 to S3

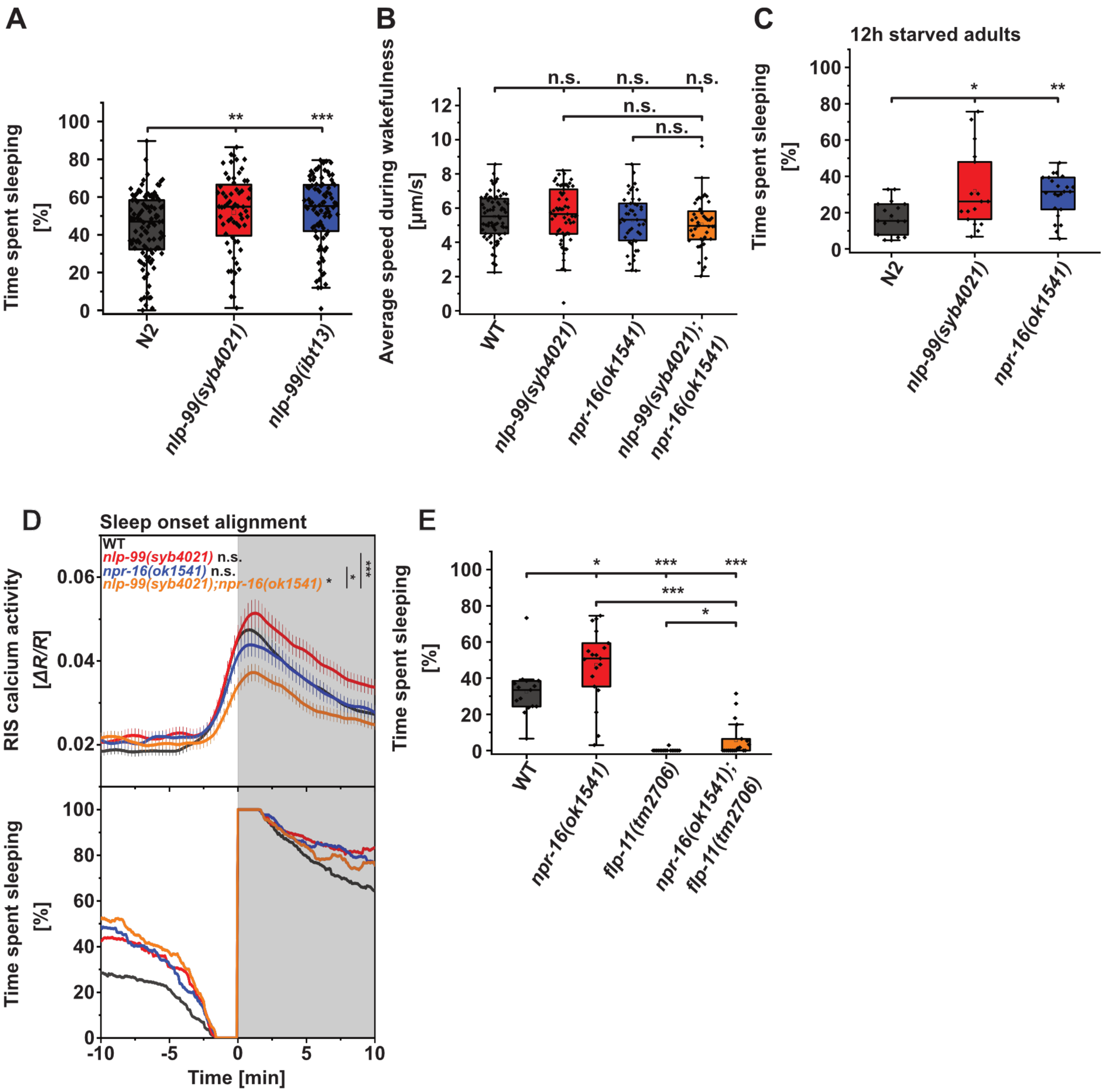

Fig. S1. NLP-99 and NPR-16 inhibit sleep during L1 arrest and in starved adults

(**A**) Two independent alleles of *nlp-99* display an increased fraction of time spent sleeping in arrested L1 larvae. Wild type (mean = 43.80%, n = 110 animals, 6 replicates, the same animals from Fig. 1H were used for this analysis), *nlp-99(syb4021)* (mean = 51.81%, n = 64 animals, 4 replicates), and *nlp-99(ibt13)* (mean = 52.33%, n = 90 animals, 3 replicates).

(**B**) There is no observable difference in movement speed during wakefulness in arrested L1 larvae of *nlp-99* and *npr-16* mutants. Wild type (mean = 5.50 µm/s), *nlp-99(syb4021)* (mean = 5.60

µm/s), *npr-16(ok1541)* (mean = 5.28 µm/s), and *nlp-99(syb4021); npr-16(ok1541)* (mean = 5.05 µm/s). The same animals from Fig. 1D were used for this analysis.

(**C**) *nlp-99* and *npr-16* also limit the time spent sleeping in 12-hour starved adult worms. Wild type (mean = 17.3%, n = 17, 5 replicates), *nlp-99(syb4021)* (mean = 31.9%, n = 17, 5 replicates), and *npr-16(ok1541)* (mean = 29.5%, n = 23, 5 replicates).

(**D**) Sleep bout alignment of the RIS calcium signal in the nerve ring. We extracted the data from the same animals used for RIS cell body calcium measurements shown in Fig. 1 A-G. Specifically, we reanalyzed the three replicates from Fig. 1A-G that included all strain combinations.

(**E**) *flp-11(tm2706)* suppresses most of the increased sleep observed in *npr-16(ok1541)*. Wild type (mean = 32.5%, n = 15, 5 replicates), *npr-16(ok1541)* (mean = 47.3%, n = 19, 5 replicates), *flp-11(tm2706)* (mean = 2.9%, n = 16, 5 replicates), and *npr-16(ok1541); flp-11(tm2706)* (mean = 5.7%, n = 19, 5 replicates).

Statistical significance was assessed using the Mann-Whitney U test. n.s. = not significant, * = $p < 0.05$, ** = $p < 0.01$, and *** = $p < 0.001$.

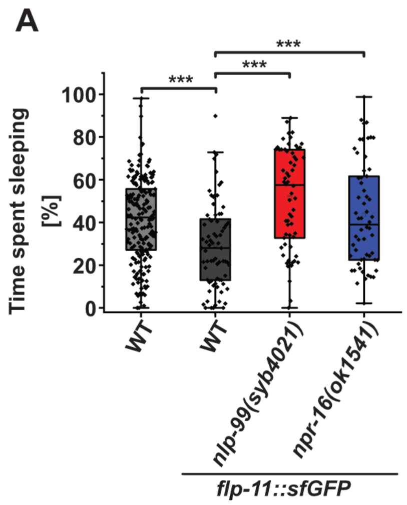

Fig. S2. Characterization of sleep in *flp-11::sfGFP*

The translational fusion allele of *flp-11* (*flp-11::sfGFP*) reduces sleep. However, the increased sleep phenotype of both the *nlp-99* and the *npr-16* deletion mutant are preserved in this transgenic background. Wild type (mean = 41.4%, n = 197 animals, 15 biological replicates, we used the same animals from Figure 1A, 1H, 1I and S1A for this analysis). Wild type containing *flp-11::sfGFP* (mean = 29.2%, n = 74 animals, 5 biological replicates, we used the same animals from Figure 2C and 2D for this analysis), *nlp-99(syb4021)* containing *flp-11::sfGFP* (mean = 51.6%, n = 62 animals, 4 biological replicates, we used the same animals from Figure 2C and 2D for this analysis), and *npr-16(ok1541)* containing *flp-11::sfGFP* (mean = 43.6%, n = 58 animals, 4 biological replicates, we used the same animals from Figure 2C and 2D for this analysis). We assessed statistical significance using the Mann-Whitney U test. *** = $p < 0.001$.

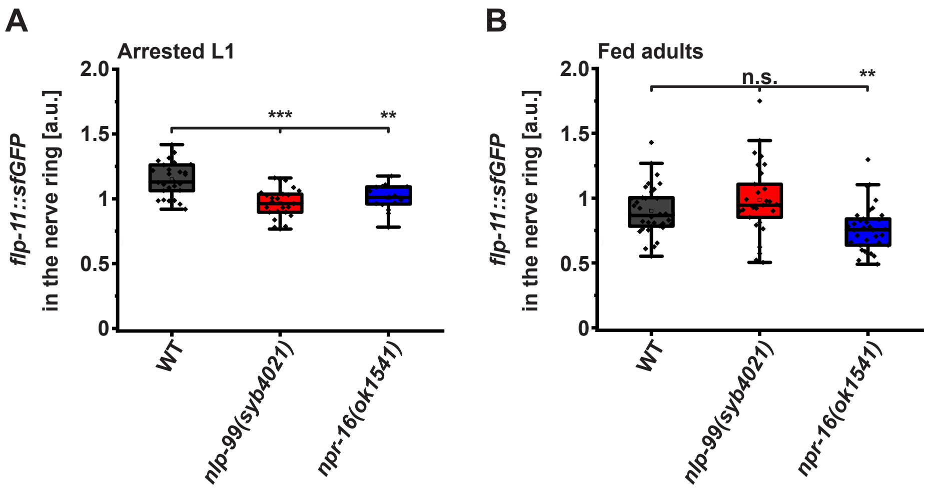

Fig. S3. NLP-99 and NPR-16 inhibit FLP-11 release from RIS during L1 arrest, whereas only NPR-16 clearly has this function in well-fed adults.

(A-B) We quantified the translational (*flp-11::sfGFP*) and transcriptional (*flp-11p::mKate2*) reporter of *flp-11* in the nerve ring of RIS and calculated *flp-11::sfGFP*/*flp-11p::mKate2* as a normalized measure of secretion. (A) During L1 arrest, deletion of *nlp-99* and *npr-16* decrease *flp-11::sfGFP*/*flp-11p::mKate2*. Wild type (mean = 1.15 a.u., n = 31, 3 replicates), *nlp-99(syb4021)* (mean = 0.97 a.u., n = 25, 3 replicates), and *npr-16(ok1541)* (mean = 1.01 a.u., n = 16, 2 replicates). (B) In well-fed adults, deletion of *npr-16* decrease *flp-11::sfGFP*/*flp-11p::mKate2*. Wild type (mean = 0.90 a.u., n = 34, 3 replicates), *nlp-99(syb4021)* (mean = 0.99 a.u., n = 29, 3 replicates), and *npr-16(ok1541)* (mean = 0.77 a.u., n = 34, 3 replicates). Statistical significance was assessed using the Mann-Whitney U test. n.s. = not significant, ** = $p < 0.01$, and *** = $p < 0.001$.

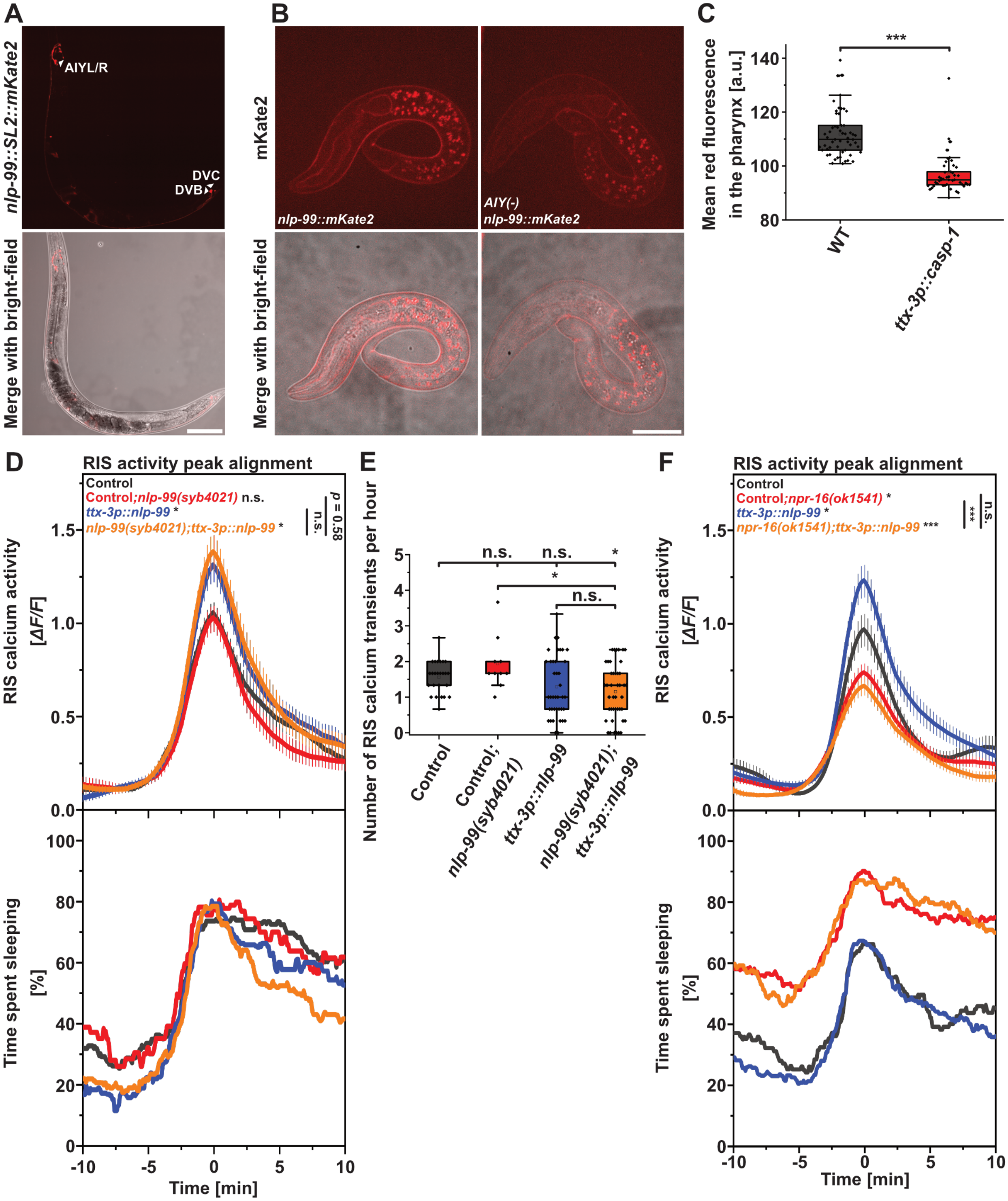

Fig. S4. AIY neurons release NLP-99 to inhibit sleep via NPR-16

(**A**) Transcriptional reporter for *nlp-99* expression. Representative images of a fed adult. AIY, DVB, DVC are clearly visible and RIS becomes visible only when increasing the brightness of the image. Scale bar represents 100 µm.

(**B**) Ablation of the AIY neurons during L1 arrest leads to a strong reduction of NLP-99::mKate2 around the pharyngeal. Representative images. Scale bar represents 25 µm.

(**C**) Quantification of secreted NLP-99::mKate2 around the pharynx in wild-type and AIY-ablated. Wild type (mean = 111.8 a.u., n = 55, 1 replicate) and *ttx-3p::casp-1* (mean = 96.8 a.u., n = 45, 1 replicate).

(**D**) Overexpression of *nlp-99* in AIY neurons increases RIS calcium activity. Control (no visible array expression) (mean = 1.05 a.u.), control (array lost) *nlp-99(syb4021)* (mean = 1.02 a.u.), *ttx-3p::nlp-99* (mean = 1.31 a.u.), and *nlp-99(syb4021);ttx-3p::nlp-99* (mean = 1.38 a.u.). The same animals from Fig. 3E were used for this analysis.

(**E**) Overexpression of *nlp-99* in AIY neurons moderately increases RIS calcium activity transient frequency. Control (no visible array expression) (mean = 1.5 bouts per hour), control (array lost) *nlp-99(syb4021)* (mean = 1.9 bouts per hour), *ttx-3p::nlp-99* (mean = 1.3 bouts per hour), and *nlp-99(syb4021);ttx-3p::nlp-99* (mean = 1.2 bouts per hour). The same animals from Fig. 3E were used for this analysis.

(**F**) Quantification of RIS activity for *nlp-99* overexpression in *npr-16(ok1541)*. Control (no visible array expression) (mean = 0.96 a.u.), control;*npr-16(ok1541)* (mean = 0.74 a.u.), *ttx-3p::nlp-99* (mean = 1.23 a.u.), and *npr-16(ok1541);ttx-3p::nlp-99* (mean = 0.66 a.u.). The same animals from Fig. 3F were used.

Statistical significance was assessed using the Mann-Whitney U test. n.s. = not significant, * = $p < 0.05$, ** = $p < 0.01$, and *** = $p < 0.001$.

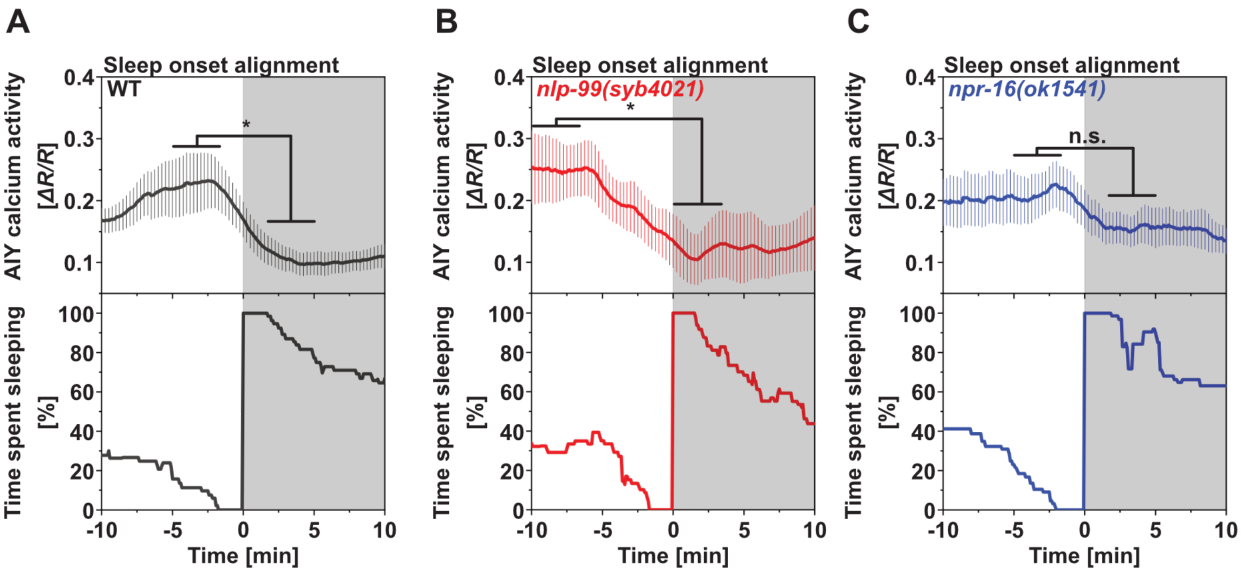

Fig. S5. The AIYs are wake-active neurons

(**A**-**C**) The calcium activity of the AIY neurons is reduced during sleep. The results from Fig. 4A were split into individual groups to show the points for statistical testing. To compare calcium activity in AIY before and after sleep onset, the following time points were used (we used different times for the genotypes to account for the differences in their calcium dynamics): wild type: before sleep (-5 to -2 min), after sleep (2 to 5 min); *nlp-99(syb4021)*: before sleep (-10 to -5 min), after sleep (0 to 3 min); *npr-16(ok1541)*: before sleep (-5 to -2 min), after sleep (2 to 5 min). Statistical significance was assessed using the paired Wilcoxon Signed-Rank Test. n.s. = not significant, * = $p < 0.05$.

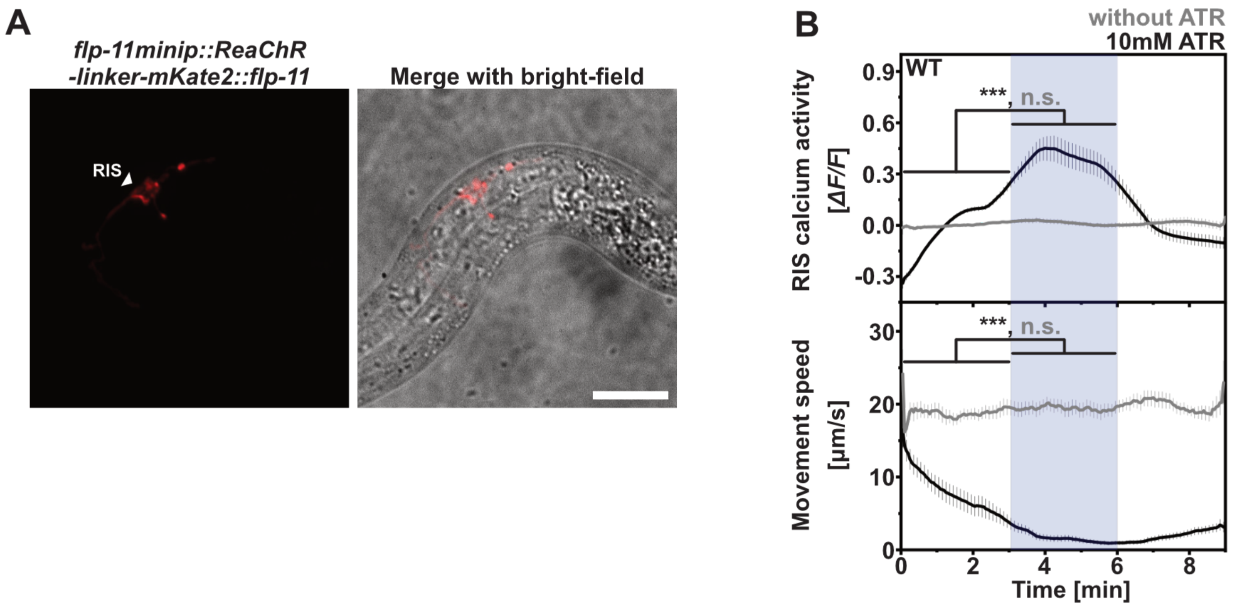

Fig. S6. Characterization of a transgene for optogenetic activation of RIS

(**A**) Representative fluorescence image of *lgc-38(syb2346syb2493syb8234[flp-11-5'utr(620bp)::ReaChR-linker-mKate2::flp-11b-3'UTR]) III:7007600* (*flp-11minip::ReaChR-linker-mKate2::flp-11)* expression in RIS. The ReaChR-linker-mKate2 is visible at the RIS cell membrane. The scale bar represents 30 µm.

(B) Optogenetic activation of RIS increases calcium activity in the presence of ATR. The blue shading indicates the stimulation period (3 to 6 min) during which orange light was applied. Wild type without ATR (n = 16, 1 replicate) and wild type with ATR (n = 30, 3 replicates). Average calcium activity during the baseline (0 to 3 min) and stimulation (3 to 6 min) periods was compared between conditions with and without ATR. Baseline conditions in the presence of ATR appeared to increase RIS activation even before the intended stimulation period, likely due to the blue fluorescence imaging light. Therefore, it is preferable to perform baseline imaging in the absence of retinal and add ATR just before optogenetic stimulation.

Statistical significance was assessed using the paired Wilcoxon Signed-Rank Test. n.s. = not significant, *** = $p < 0.001$.

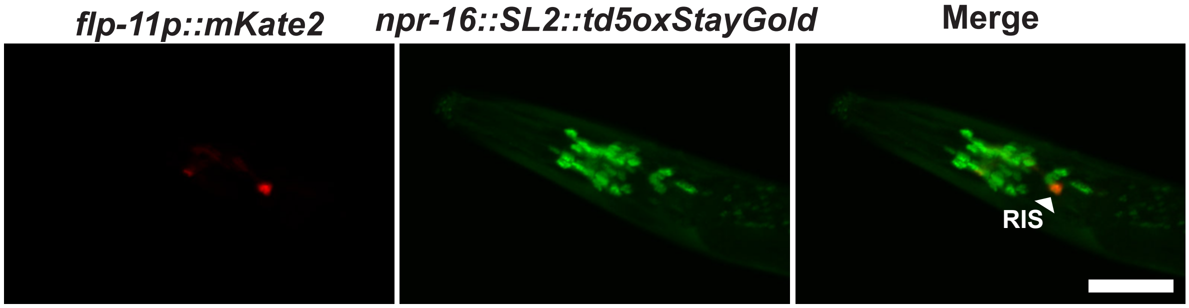

Fig. S7. In fed adults, *npr-16* is still expressed in RIS, but expression in RIS is no longer among the strongest, with multiple head neurons expressing *npr-16* at comparable or higher levels

Representative images of *npr-16* expression in well-fed adults. We combined *npr-16::SL2::td5oxStayGold* with *flp-11p::mKate2* to identify RIS. Scale bar represents 50 µm.

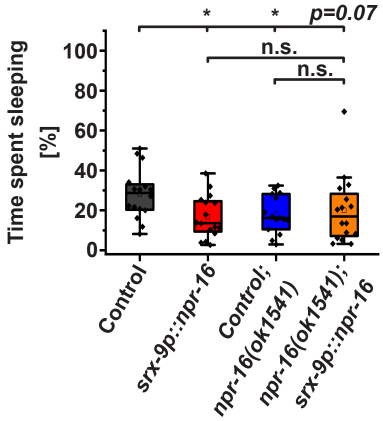

Fig. S8. In fed adults and similar to arrested larvae, NPR-16 acts outside of RIS to support sleep, whereas RIS-expressed NPR-16 inhibits sleep.

Overexpression of *npr-16* in RIS in fed adults. Control (no visible array expression) (mean = 28.2%, n = 16, 3 replicates), *srx-9p::npr-16* (mean= 16.7%, n = 16, 3 replicates), control;*npr-16(ok1541)* (mean = 18.0%, n = 15, 3 replicates), and *npr-16(ok1541); srx-9p::npr-16* (mean = 20.0%, n = 16, 3 replicates). Statistical significance was assessed using the Mann-Whitney U test. n.s. = not significant, * = p < 0.05.

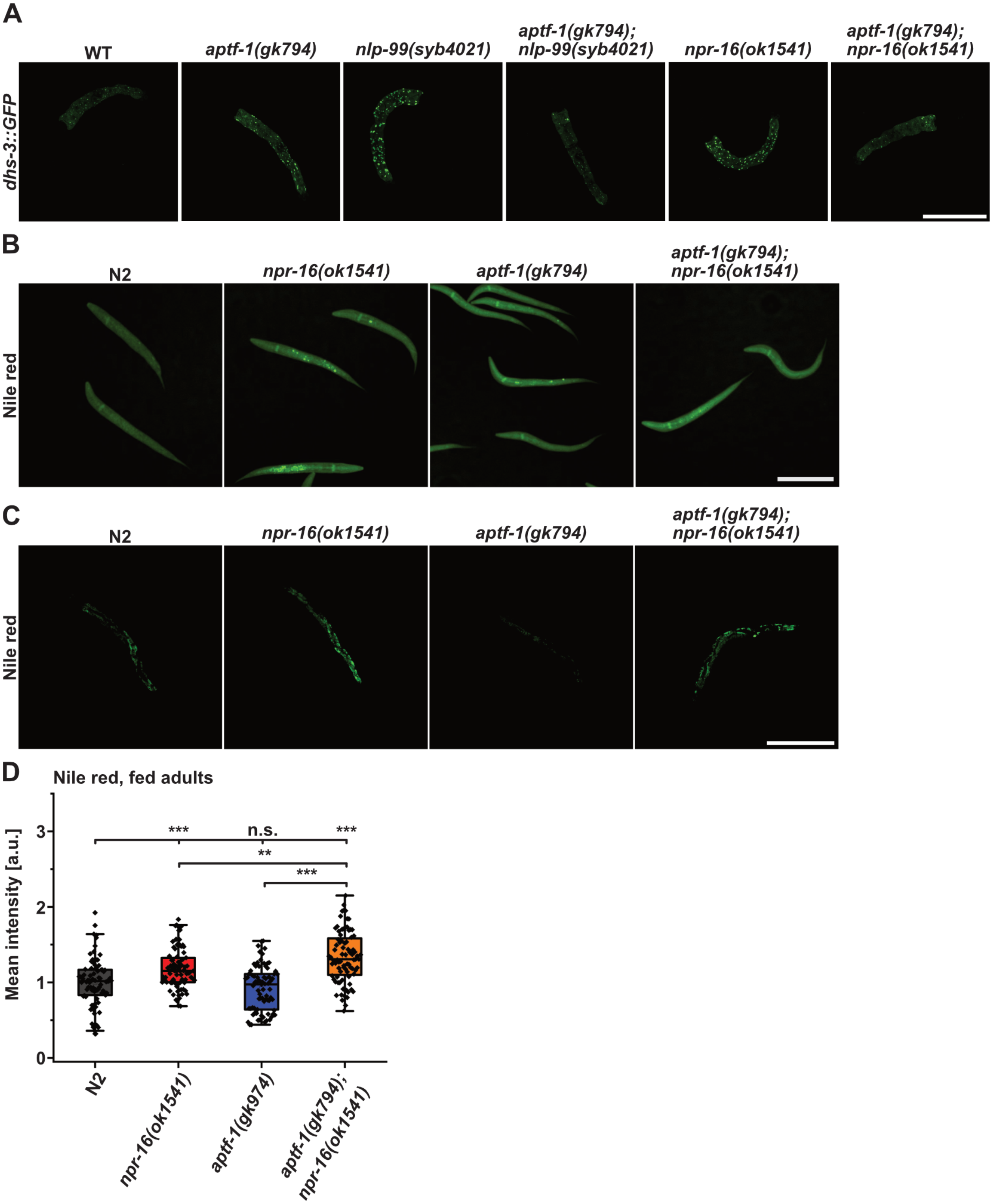

Fig. S9. Lipid staining in the *npr-16* deletion mutant

(**A**) Representative images of DHS-3::GFP transgenic reporter animals in L1 arrest, shown for the different mutant backgrounds analyzed. The scale bar represents 50 µm.

(**B**) Sample images of Nile Red lipid staining in L1 arrested larvae. L1 larvae typically have low fat levels, which makes fat quantification challenging. However, in many *npr-16* deletion mutant animals, fat levels were substantially higher, allowing for clear differences in signal compared to wild-type animals. Gut granules are generally smaller and round, whereas fat droplets are larger and irregularly shaped - features that are particularly prominent in *npr-16* mutants. Since L1 larvae are largely depleted of gut granules, the Nile Red signal primarily reflects fat droplets. Applying a signal threshold allows reliable isolation of fat-specific signal. Scale bar: 100 µm.

(**C**) Sample images of Nile Red lipid staining in well-fed adult. Scale bar: 500 µm.

(**D**) Quantification of Nile Red staining in well-fed adults. The scenario in the presence of food is more complex, as sleep affects the amount of time animals spend feeding. Our results in well-fed adults show that NPR-16 inhibits lipid storage. However, the results from *aptf-1(-)* animals are less conclusive, as the increased lipid stores observed in *npr-16* mutants do not depend on *aptf-1*. One possible explanation is that *aptf-1* mutant animals, which do not sleep, spend more time feeding. This increased feeding could mask a potential lipid storage-promoting role of RIS. Therefore, the experiments are more straightforward to interpret in the absence of food.

Statistical significance was assessed using the Mann-Whitney U test. n.s. = not significant, * = $p < 0.05$, ** = $p < 0.01$, and *** = $p < 0.001$.

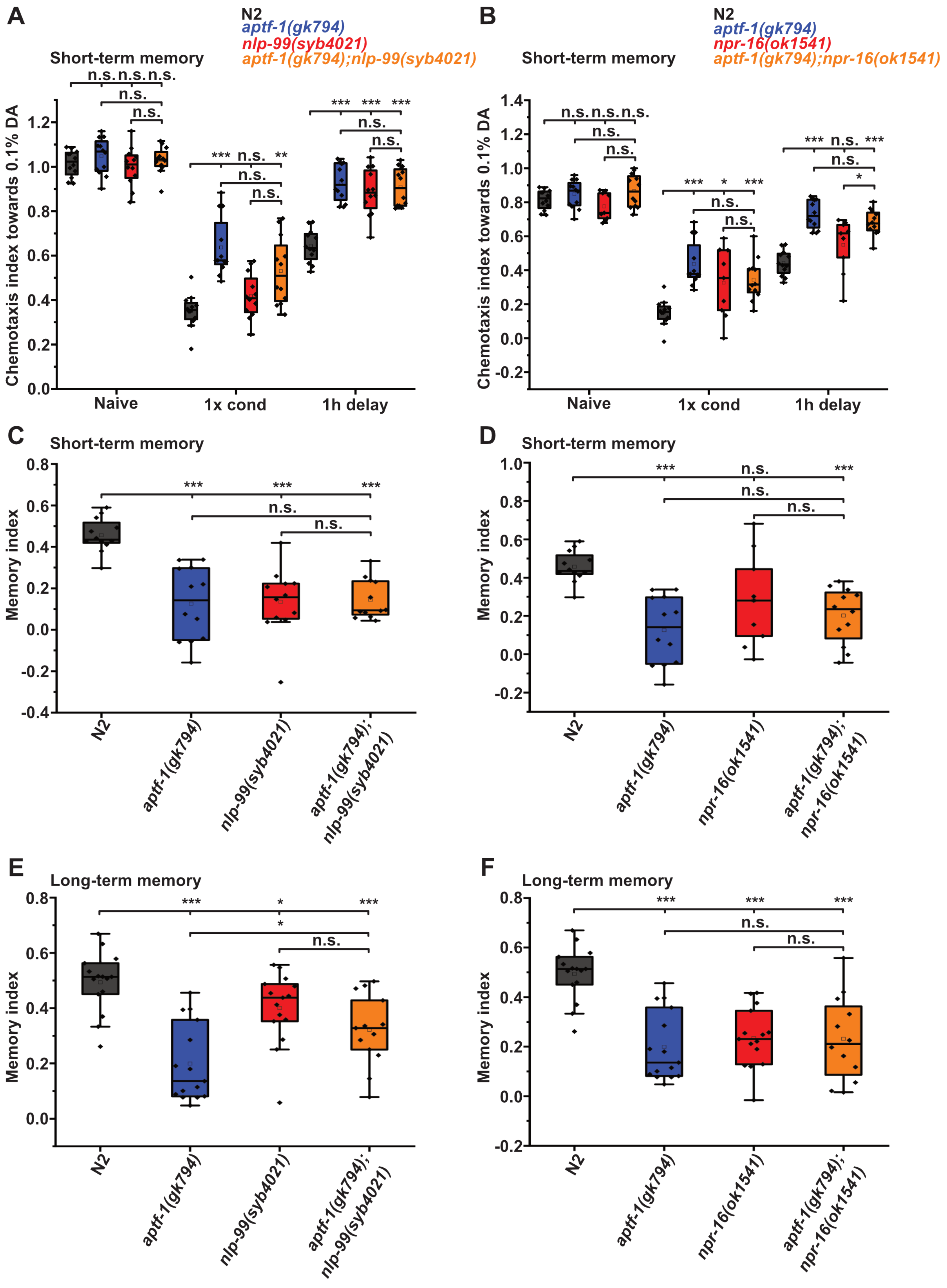

Fig. S10. NLP-99/NPR-16 and APTF-1/FLP-11 are required for adult learning

(**A**-**B**) Short-term memory assay reveals a role of *npr-16 in learning* (15 replicates).

(**C**-**F**) Memory index calculation supports the roles of *aptf-1*, *flp-11, nlp-99* and *npr-16* in memory formation. Same animals as were used for Fig. 7.

Statistical significance was assessed using the Mann-Whitney test. n.s. = not significant, * = $p < 0.05$, ** = $p < 0.01$, and *** = $p < 0.001$.

Data S1. (separate file)
Sequences of alleles created for this study.

Table S1. (separate file)
Description of *C. elegans* strains used for this study.

Table S2. (separate file)
Sequences of primers used for this study.

Table S3. (separate file)
Sequences of crispr guide and repair sequences used for this study.
