# Supplementary material for: C. elegans somatostatin/allatostatin C signaling regulates sleep, metabolism, survival, and memory via a sleep-active neuron
Source: Sci Adv. 2026 Apr 15;12(16):eadv8387. doi: 10.1126/sciadv.adv8387 (PMC13082328; doi:10.1126/sciadv.adv8387)
Supplement: Supplementary file 3 — Data S1 [file sciadv.adv8387_data_s1.zip › adv8387_data_s1.docx]

**Data S1**

>pUC57-Mini-*ttx-3p::T05A8.3::SL2::mKate2::unc-54-3’UTR*; ttx-3p(636 bp upstream of main ATG + 2^nd^ intron 243 bp) - T05A8.3 CAI-1.0 - SL2(*gpd-2*) - *mKate2* - *unc-54* *3’UTR*

agatgcacgcacaactgcacatatattgccatatagaattatttctataatacatataaaaataaaagtaaaagtctgtaacgtttatttttaacttttaaataagttcatatagtgcaaatgaattttcggcatttgtattttggagaactactaaaacatttgtattatgtttcctttttctgtataacatttttcttaaaaattgttcaaacattttttatgtttcggcttgcttttatataatttttggttttttcacaacttatcgtcagcatttatgatttctaatcacctcattatgttctggtcagctgcagtttatattcaaatcccaaatgacacgaaaaaaagttaatgatgataactaacaatcaattgacgtaaacaagtcagtagtctcttttgccataactgagcatacgcttcgtcggactaaattccaatcccgctcattgatctgtaacgtgaaaacttttacacttccaaccccgcgacatgtccgtatattcaactgacattatcacaagtttgacaaagtcttcaagcttgagtattcttcctgcacctctatctctccctctcgcgcaacatgactttccatatgcttggatatcaatgcttcctacttttcaagtgagcatctattacagcgattttctctaatttaacccgaaattataacctgtttttttttcgaaaatgactcatttttctcgaatacagagttgagaataaaaatagcaaggtcgtgtattttatcatgtcgctatttttcttaacgaagccaataacaatgccacttatttcaagtcttgagaaacaaattatttgacgaaaatgtttttttttcaaagatttactgctttaactttacagaaaaATGGCCTCCTCCACCTCCACCACCCTCTTCTTCCTCCTCCTCGTCGCCACCTCCTCCATGATGATGGGACACGTCTCCGCCGGACGTATCATCCCACAATCCTACACCGGAGTCGACCCAGGAATCCAATTCGAGCTCCTCACCGCCAAGCGTGGAGACGGATACGGATGGAACGACTGCGAGTTCTCCCCACTCTCCTGCCTCCTCAAGCGTCGTCGTCGTTCCGCCTAAgctgtctcatcctactttcacctagttaactgcttgtcttaaaatctatgcttctctttagtatctaaaattttcctagaagcttacaagtatataaatggtctcttctcaataaaggttgtatatttattcatcttattgaatctgccatttcctcgtttttgcgagtttatataccttccaattttctttctattgtattttcaacttctaattttaattcagggaaactgcttcaacgcatcATGTCCGAGCTCATCAAGGAGAACATGCACATGAAGCTCTACATGGAGGGAACCGTCAACAACCACCACTTCAAGTGCACCTCCGAGGGAGAGGGAAAGCCATACGAGGGAACCCAAACCATGCGTATCAAGgtaagtttaaacatatatatactaactaaccctgattatttaaattttcagGCCGTCGAGGGAGGACCACTCCCATTCGCCTTCGACATCCTCGCCACCTCCTTCATGTACGGATCCAAGACCTTCATCAACCACACCCAAGGAATCCCAGACTTCTTCAAGCAATCCTTCCCAGAGGGATTCACCTGGGAGCGTGTCACCACCTACGAGGACGGAGGAGTCCTCACCGCCACCCAAGACACCTCCCTCCAAGACGGATGCCTCATCTACAACGTCAAGATCCGTGGAGTCAACTTCCCATCCAACGGACCAGTCATGCAAAAGAAGACCCTCGGATGGGAGGCCTCCACCGAGACCCTCTACCCAGCCGACGGAGGACTCGAGGGACGTGCCGACATGGCCCTCAAGCTCGTCGGAGGAGGACACCTCATCTGCAACCTCAAGgtaagtttaaacatgattttactaactaactaatctgatttaaattttcagACCACCTACCGTTCCAAGAAGCCAGCCAAGAACCTCAAGATGCCAGGAGTCTACTACGTCGACCGTCGTCTCGAGCGTATCAAGGAGGCCGACAAGGAGACCTACGTCGAGCAACACGAGGTCGCCGTCGCCCGTTACTGCGACCTCCCATCCAAGCTCGGACACCGTTAAGTCCAATTACTCTTCAACATCCCTACATGCTCTTTCTCCCTGTGCTCCCACCCCCTATTTTTGTTATTATCAAAAAACTTCTCTTAATTTCTTTGTTTTTTAGCTTCTTTTAAGTCACCTCTAACAATGAAATTGTGTAGATTCAAAAATAGAATTAATTCGTAATAAAAAGTCGAAAAAAATTGTGCTCCCTCCCCCCATTAATAATAATTCTATCCCAAAATCTACacaatgttctgtgtacacttcttatgttttttacttctgataaatttttttgaaacatcatagaaaaaaccgcacacaaaataccttatcatatgttacgtttcagtttatgaccgcaatttttatttcttcgcacgtctgggcctctcatgacgtcaaatcatgctcatcgtgaaaaagttttggagtatttttggaatttttcaatcaagtgaaagtttatgaaattaattttcctgcttttgctttttggggtttcccctattgtttgtcaagatttcgaggacggcgtttttcttgctaaaatcacaagtattgatgagcacgatgcaagaaagatcggaagaaggtttgggtttgaggctcagtggaaggtgagtagaagttgataatttgaaagtggagtagtgtctatggggtttttgccttaAATGACAGAATACATTCCCAATATACCAAACATAACTGTTT

>pHB86(*srx-9p::npr-16a**::SL2::mKate2::**let-858 3’UTR)*

aagcttcattttggattggcgagatctactgagacctaaaagagagagcgccactccaaaatgaacctgttactactgggaactccggagtaaatatcctttacaactaacccatgttctatcagttgcaattaaaagagccagaatggatctagtagatcctgttatatatcttgcccagaatatgaaaaacattgaaatttctacaatatgggagagctcttggaatttggccatagtaatcaaaattgaaccaagaaaaaatataactgaaagagtttcgatgattttttaataaaagctttgcacatacttatagtactgaatactatgtccaacaaaaatcgaaaatagaaaagggtcaaatcatgtgatttcttgagttttttaaacctgaaaatagatttttaaatgatggaaaaatgttgatatgggcagtggctaccaaaatatgtctagcagtaattttgtgcaaaagaatattgttataaatgcgcgaccaattgctgtaactgtaatgaatacaccaaaaaaatacatgtcttctttatatcccactttgttttcaagttaagtcagcacgttttccaaaaaaattaacacactggttcataatcaaaagtcaaaacttttattaaaatttggagcaaacgtttattcactgaaaggttatgcattttagttgtggttgttcagtacataaaaacttttaaaaaaatcgacacgaaaaaaacaaaaaaaaatgaagaaaaaggaaatggacaacgtttattttgaaactgtattgttttatggggttattcaagtagtgtcgtgaaattaaaaagtgtatttcagtacactttttgccttattcaagtaacatgaaaaacatgtatttcaatacagtttgtgaagtaatttcgcgacatttctcgacattatgcgacaaaagcggacaactgaatgaaaaacacagagaaaggtgtaccgcagggtgaaaatctacacaatttgccgtcgcaggagaacaattctgataaaaccactgtggaaaatgtgtcgagaaatgtcgagaaatgtcacgaaattacgtcacaaactgtattgaaatgtttttgaagttacttgaataagacaaaaagtgtattgaaatacactttttaatttcacgacactacttgaataaccccattagatagatttcgaaacaaaacttcagattctgaaactccgtgcctaacaatgatgtacttgaattatatatgtatgtcaaaattgttaatattaaaaaaccgcgcaaactcacagtataccgcttataaatgtgttcgttgttttgctgttcacagaatgcttatatgtaatatatcgttataaatctataaattaatatagcattggctgaatgactaaacctttgttcacggaaccaaactagcatctcaaatcaaatcagaaatatcacaggaaaatttatttttcctattcttcgaaaataagttttgaacggaaaaacgaatctcaggtttagaatgaaaaacagaagttcgcacagatattgtaatctcaatttaattagtattctttttttaggaaacaacctttccaaatggaatatatgatacaattcaatttaatgattgcccgatgtggtaaactcgatatgacctcaccagaattcataattattcttaaacaaaataaaaaattgtatgaaaagtttttaaggtcgcaaatattttgtaaatctaatcgtgggtaaaaaattcaaatgtgaaagttcatgacaagtgggtcattttcttgaaatccccatttggattggctaatggcaagaatttacagtatttatctaatattgccatatgtttcccggaaaaatatgtttaatgtacatataggtaattattgcgtaactattaacgactagacatagaggaaatcagtaaattggattctatatccgcataaacaatgtgagattcaaattgagatacattcagtgccataatcattcacaaataaaaATGAGCGATTCCTCGCAACACCTACGGCTTGCGCTGACGGTCACACATCTGCTGCTCGTCGCTTTGGGATCCGTCAACCTCATCATTATTCTGTTAATTATCACAAGACCATACCTCCGATCTATAACAAATGTGTACATGATAGGATTATGTCTTGCTGATTTCATATATCTAGCAGATTTAATACTCGTTGCCGCCACATCACTAAATGGCAAAAGCTGGCCGTTTGGTCCAACAATTTGTCATCTTTTCCATGGCACGGAGGCGACGGGGAAATACGCATCTGTACTTTTCGTCGTACTTCTAGCCGCTGATCGATACATCGCGATGTGTAAAAGTGATTTGTGCGGCAGATATAGAACTTATCGCACAGCCATCTTCCTGAGCGGTCTTGCGTGGATCGCCGCGTTTATCTGCAGTCTTCCTCTCTACGTGTACGCCGATGAGATAAAAGTACGTATGAGACCGAAGAATGGTACAGATTCGATAGAGAACAATCATACATTGTGTATTGCTCATTGGCCAAGTCCACCACACGCACAGTGGTATATCTCTGTGTGCTCGGTGATGATCTTCATACTTCCGGGACTTGTGATTTTCTACTGCTATTATCATGTATTTTGTAAATTACGAGAAGCTGCAAAGGGTTCTAGGAGATTGCATCGTAATAAGAGATCAAGGTCTTCATATCAAAGAGTCACTCGAAGTGTACAACGGGTTGTATTGTTTCATTTACTTTGTTGGTCCCCATTCTGGTTGTTCAATTTGTTTTCTGCCATCTTCCGGGTTCGAATAACAACACAATTAATGAGAATCATAGTCAACATCATTCACCTCTTCCCTTATGTGAACTGTGCACTTAATCCAGTATTATATGCATACCGAGCAGAAAACTTCCGGACCGCATTCAAATCACTGCTCTTCTGGAGCCGACGGTCAGTTTCTAGCAAATTAAACCGACCACTTCCCTTACCGGAAGATGTGCGGAGCACGTCGGCTAGTACGTACTATAGGAACAGCAGCAACTTGGACTGTTTGAAATCCTCGTTGCCCATAAAGTCAACAATGGAGCCAGCATCCGTGTACCAACCAGAAGAAGAAAGTCCAGTGGACTCTACTGAAAACTTGGAAATGGAGAGAAAAAAAGCGGAACTTCAAGCCTGGCGGCCTTATGATGGAACTAACTGTGAAGTATTAGAAGTTCAATACGATCATCATAAATGCTCCGCCAGAAGTATCCTTCTCCCAGCTGTCACAGTGGACGAAGGAACAAAATTATGAgctgtctcatcctactttcacctagttaactgcttgtcttaaaatctatgcttctctttagtatctaaaattttcctagaagcttacaagtatataaatggtctcttctcaataaaggttgtatatttattcatcttattgaatctgccatttcctcgtttttgcgagtttatataccttccaattttctttctattgtattttcaacttctaattttaattcagggaaactgcttcaacgcatcATGTCCGAGCTCATCAAGGAGAACATGCACATGAAGCTCTACATGGAGGGAACCGTCAACAACCACCACTTCAAGTGCACCTCCGAGGGAGAGGGAAAGCCATACGAGGGAACCCAAACCATGCGTATCAAGgtaagtttaaacatatatatactaactaaccctgattatttaaattttcagGCCGTCGAGGGAGGACCACTCCCATTCGCCTTCGACATCCTCGCCACCTCCTTCATGTACGGATCCAAGACCTTCATCAACCACACCCAAGGAATCCCAGACTTCTTCAAGCAATCCTTCCCAGAGGGATTCACCTGGGAGCGTGTCACCACCTACGAGGACGGAGGAGTCCTCACCGCCACCCAAGACACCTCCCTCCAAGACGGATGCCTCATCTACAACGTCAAGATCCGTGGAGTCAACTTCCCATCCAACGGACCAGTCATGCAAAAGAAGACCCTCGGATGGGAGGCCTCCACCGAGACCCTCTACCCAGCCGACGGAGGACTCGAGGGACGTGCCGACATGGCCCTCAAGCTCGTCGGAGGAGGACACCTCATCTGCAACCTCAAGgtaagtttaaacatgattttactaactaactaatctgatttaaattttcagACCACCTACCGTTCCAAGAAGCCAGCCAAGAACCTCAAGATGCCAGGAGTCTACTACGTCGACCGTCGTCTCGAGCGTATCAAGGAGGCCGACAAGGAGACCTACGTCGAGCAACACGAGGTCGCCGTCGCCCGTTACTGCGACCTCCCATCCAAGCTCGGACACCGTTAAattttcaaattttaaatactgaatatttgttttttttcctattatttatttattctctttgtgttttttttcttgctttctaaaaaattaattcaatccaaatctaaacatttttttttctctttccgtctcccaattcgtattccgctcctctcatctgaacacaatgtgcaagtttatttatcttctcgctttcatttcattaggacgtggggggaattggtggaagggggaaacacacaaaaggatgatggaaatgaaataaggacacacaatatgcaacaacattcaattcagaaatatggaggaaggtttaaaagaaaacataaaaatatatagaggaggaaggaaaactagtaaaaaataagcaaagaaattaggcgaacgatgagaattgtcctcgcttggactagtcggccgtacgggccctttcgtctcgcgcgtttcggtgatgacggtgaaaacctctgacacatgcagctcccggagacggtcacagcttgtctgtaagcggatgccgggagcagacaagcccgtcagggcgcgtcagcgggtgttggcgggtgtcggggctggcttaactatgcggcatcagagcagattgtactgagagtgcaccatatgcggtgtgaaataccgcacagatgcgtaaggagaaaataccgcatcaggcggccttaagggcctcgtgatacgcctatttttataggttaatgtcatgataataatggtttcttagacgtcaggtggcacttttcggggaaatgtgcgcggaacccctatttgtttatttttctaaatacattcaaatatgtatccgctcatgagacaataaccctgataaatgcttcaataatattgaaaaaggaagagtatgagtattcaacatttccgtgtcgcccttattcccttttttgcggcattttgccttcctgtttttgctcacccagaaacgctggtgaaagtaaaagatgctgaagatcagttgggtgcacgagtgggttacatcgaactggatctcaacagcggtaagatccttgagagttttcgccccgaagaacgttttccaatgatgagcacttttaaagttctgctatgtggcgcggtattatcccgtattgacgccgggcaagagcaactcggtcgccgcatacactattctcagaatgacttggttgagtactcaccagtcacagaaaagcatcttacggatggcatgacagtaagagaattatgcagtgctgccataaccatgagtgataacactgcggccaacttacttctgacaacgatcggaggaccgaaggagctaaccgcttttttgcacaacatgggggatcatgtaactcgccttgatcgttgggaaccggagctgaatgaagccataccaaacgacgagcgtgacaccacgatgcctgtagcaatggcaacaacgttgcgcaaactattaactggcgaactacttactctagcttcccggcaacaattaatagactggatggaggcggataaagttgcaggaccacttctgcgctcggcccttccggctggctggtttattgctgataaatctggagccggtgagcgtgggtctcgcggtatcattgcagcactggggccagatggtaagccctcccgtatcgtagttatctacacgacggggagtcaggcaactatggatgaacgaaatagacagatcgctgagataggtgcctcactgattaagcattggtaactgtcagaccaagtttactcatatatactttagattgatttaaaacttcatttttaatttaaaaggatctaggtgaagatcctttttgataatctcatgaccaaaatcccttaacgtgagttttcgttccactgagcgtcagaccccgtagaaaagatcaaaggatcttcttgagatcctttttttctgcgcgtaatctgctgcttgcaaacaaaaaaaccaccgctaccagcggtggtttgtttgccggatcaagagctaccaactctttttccgaaggtaactggcttcagcagagcgcagataccaaatactgtccttctagtgtagccgtagttaggccaccacttcaagaactctgtagcaccgcctacatacctcgctctgctaatcctgttaccagtggctgctgccagtggcgataagtcgtgtcttaccgggttggactcaagacgatagttaccggataaggcgcagcggtcgggctgaacggggggttcgtgcacacagcccagcttggagcgaacgacctacaccgaactgagatacctacagcgtgagcattgagaaagcgccacgcttcccgaagggagaaaggcggacaggtatccggtaagcggcagggtcggaacaggagagcgcacgagggagcttccagggggaaacgcctggtatctttatagtcctgtcgggtttcgccacctctgacttgagcgtcgatttttgtgatgctcgtcaggggggcggagcctatggaaaaacgccagcaacgcggcctttttacggttcctggccttttgctggccttttgctcacatgttctttcctgcgttatcccctgattctgtggataaccgtattaccgcctttgagtgagctgataccgctcgccgcagccgaacgaccgagcgcagcgagtcagtgagcgaggaagcggaagagcgcccaatacgcaaaccgcctctccccgcgcgttggccgattcattaatgcagctggcacgacaggtttcccgactggaaagcgggcagtgagcgcaacgcaattaatgtgagttagctcactcattaggcaccccaggctttacactttatgcttccggctcgtatgttgtgtggaattgtgagcggataacaatttcacacaggaaacagctatgaccatgattacgccaagctgtaagtttaaacatgatcttactaactaactattctcatttaaattttcagagcttaaaaatggctgaaatcactcacaacgatggatacgctaacaacttggaaatgaaat

>wild type locus of *nlp-99*, T05A8.3.1, encodes the predicted NLP-99 peptide

gaatgtcattctttgcgcgcttcaaccttctaagccgcgtgcacaacttttcctgtgtgcctgtgtgctgctccactagaataATGGCATCCTCCACCTCCACAACACTATTCTTCCTTCTGCTCGTTGCCACGTCATCAATGATGATGGGACACGTCTCCGCGGGCCGAATTATACCACAGgtgagaatctacaaaaaattgagcttaaattccctaaaattgagttttgagccgtgggcttggggtcttaggcttaggcttaggcttagggcaacaggtttaggtccttaggctttgggccgtaggcttagggtctcagggttagggccaggcttaagaccttagaattagggtcttaggttcaaggtcttaagcttagggtcttaggttcagggtcttaagtttaggcttaggactttagacttagggctttaggcttaaggcattaggcttaggcttaggcttaggcttaggcttaggcttaggcttaggcttaggcttaggcttaggcttaggcttaggcttaggcttaggcttaaagcctcaggcttcaggttacaatgtgactgcaaaagtctggagtttcacacaattttccgctgaaagttctaatatcccaaaattcccaaaataaacttactcccaaaatatccttgttcttcccacacacttccatggtctcatcttgaacttttttttcgccaaattttttttgccatttttatcccatacccagcttaacctcccatctattctctcattttcttgcctaaatatattttttgccccattcccccatattttttcttacccaaatccctgtcaaaagccgaaagctgtgcacacacaaactaataaagacgttgaaaaaattgaaaaaatgtgaacctaaattgggcaataaaacatcacagaggttttatgacgtaatttcgataggcttttggtggtgctgtcatttgttcgaaaattggcaaaatcgtagagcaaagggtaggggaaattagaaatatacgacatgagcagggggggggggggggtgggggtaaaagattaattgctccaaagagcaagatgtatgaaacttttgacgttaatttgtaacagcatgagacatccgtctttatgactttttgattagggagaggtgtgagggaaggtcaccagaaacctttttagctttgaatttaaatttgtttggtgtaaaatcttatgctgatcagtttgtcaattaaaattgtttctctaattttactcgttgagaagatacaccgggttcaagtagaaagtgggaaatattgtgggaggaggggagacgcagcgactgtctgcgtctccactcctcttctactctttcagtcactgttgtggccacttatctttgaaatcctgtaacttcgcagccgttagagataatgaaaaactttaaactagcaaaatacttaaaatttctagctgaacaatttgttgattgagaattttttgctagcgtcatttcttgaagagatatctaagttacaattcaaaatgataaaactaccgtttcgaaaatcatcagaatcaaattttctgacggttttttattggttggcacattttccaaaccgatagagaaattttaaactttaagaatgctagacgagacagcctgtgtgagggagagacgcagattgctagactgtctgaatctctccccctctctcactgctgtctgcgtgacttagatttaaacgtagatatctcgaaaaccggcagagatatcaaaaaagtttcaattgacaaaatatttgaaattttgagcttaacaatttgtctgttaaaaactttttgatatttctagtaagcccagagttataatcgattgaaaaaaaaaaacttatcatcttcactagactatatctaggttgttttaaaaaatggtgaaaaattgccaattgacataacttttgttataagtttattccacaatttcagtatattcaatttgttggtttctgttagaatcaacgaagcttcgagctccaaatcggagagattccgggtcgatgcaccatgagccaaatcagttcctaactgggttaccattagttttatcgaatttttttaaaattaacataacatttgttacaaggttttctataattctgtagttgaaacttatttaatatctataaaattaatcgagatataagcttaaaaaaataatataaaaattgataatagccatttaaagccaaattttcccgccaaaaatcttcaagcttcatataattttaataaaatttctagTCCTACACCGGCGTCGACCCCGGAATCCAATTCGAGCTGCTCACCGCAAAACGTGGCGACGGATACGGATGGAACGACTGCGAGTTCTCACCACTCAGCTGCCTGCTCAAACGACGACGACGATCAGCTTAAtcgacacaagcaccacatattggatcagccagttaacttttttcggaaaaaatgaaatttattttctgaaaattagagtcccctttaacatcaattgtttatttttccattcaaaaagttttttccaccaacaaaaatttggttcgtcaccactcaagcaagcaatcactattttttctatccttcccagtttccagacaccactcccctccccttcttttcgtaattattatcatcatcatcgccggcgagcgtttacttgtcattttcttctacacaagatctttttttttctttattacattatcgatatctatgaacggaacaaaagagaaaaagttgatgattttgaaataaatcatgt

*>nlp-99(syb4021[-85bp, two stop codons])*

gaatgtcattctttgcgcgcttcaaccttctaagccgcgtgcacaacttttcctgtgtgcctgtgtgctgctccactagaataATGTAATAGCACAGgtgagaatctacaaaaaattgagcttaaattccctaaaattgagttttgagccgtgggcttggggtcttaggcttaggcttaggcttagggcaacaggtttaggtccttaggctttgggccgtaggcttagggtctcagggttagggccaggcttaagaccttagaattagggtcttaggttcaaggtcttaagcttagggtcttaggttcagggtcttaagtttaggcttaggactttagacttagggctttaggcttaaggcattaggcttaggcttaggcttaggcttaggcttaggcttaggcttaggcttaggcttaggcttaggcttaggcttaggcttaggcttaggcttaaagcctcaggcttcaggttacaatgtgactgcaaaagtctggagtttcacacaattttccgctgaaagttctaatatcccaaaattcccaaaataaacttactcccaaaatatccttgttcttcccacacacttccatggtctcatcttgaacttttttttcgccaaattttttttgccatttttatcccatacccagcttaacctcccatctattctctcattttcttgcctaaatatattttttgccccattcccccatattttttcttacccaaatccctgtcaaaagccgaaagctgtgcacacacaaactaataaagacgttgaaaaaattgaaaaaatgtgaacctaaattgggcaataaaacatcacagaggttttatgacgtaatttcgataggcttttggtggtgctgtcatttgttcgaaaattggcaaaatcgtagagcaaagggtaggggaaattagaaatatacgacatgagcagggggggggggggggtgggggtaaaagattaattgctccaaagagcaagatgtatgaaacttttgacgttaatttgtaacagcatgagacatccgtctttatgactttttgattagggagaggtgtgagggaaggtcaccagaaacctttttagctttgaatttaaatttgtttggtgtaaaatcttatgctgatcagtttgtcaattaaaattgtttctctaattttactcgttgagaagatacaccgggttcaagtagaaagtgggaaatattgtgggaggaggggagacgcagcgactgtctgcgtctccactcctcttctactctttcagtcactgttgtggccacttatctttgaaatcctgtaacttcgcagccgttagagataatgaaaaactttaaactagcaaaatacttaaaatttctagctgaacaatttgttgattgagaattttttgctagcgtcatttcttgaagagatatctaagttacaattcaaaatgataaaactaccgtttcgaaaatcatcagaatcaaattttctgacggttttttattggttggcacattttccaaaccgatagagaaattttaaactttaagaatgctagacgagacagcctgtgtgagggagagacgcagattgctagactgtctgaatctctccccctctctcactgctgtctgcgtgacttagatttaaacgtagatatctcgaaaaccggcagagatatcaaaaaagtttcaattgacaaaatatttgaaattttgagcttaacaatttgtctgttaaaaactttttgatatttctagtaagcccagagttataatcgattgaaaaaaaaaaacttatcatcttcactagactatatctaggttgttttaaaaaatggtgaaaaattgccaattgacataacttttgttataagtttattccacaatttcagtatattcaatttgttggtttctgttagaatcaacgaagcttcgagctccaaatcggagagattccgggtcgatgcaccatgagccaaatcagttcctaactgggttaccattagttttatcgaatttttttaaaattaacataacatttgttacaaggttttctataattctgtagttgaaacttatttaatatctataaaattaatcgagatataagcttaaaaaaataatataaaaattgataatagccatttaaagccaaattttcccgccaaaaatcttcaagcttcatataattttaataaaatttctagTCCTACACCGGCGTCGACCCCGGAATCCAATTCGAGCTGCTCACCGCAAAACGTGGCGACGGATACGGATGGAACGACTGCGAGTTCTCACCACTCAGCTGCCTGCTCAAACGACGACGACGATCAGCTTAAtcgacacaagcaccacatattggatcagccagttaacttttttcggaaaaaatgaaatttattttctgaaaattagagtcccctttaacatcaattgtttatttttccattcaaaaagttttttccaccaacaaaaatttggttcgtcaccactcaagcaagcaatcactattttttctatccttcccagtttccagacaccactcccctccccttcttttcgtaattattatcatcatcatcgccggcgagcgtttacttgtcattttcttctacacaagatctttttttttctttattacattatcgatatctatgaacggaacaaaagagaaaaagttgatgattttgaaataaatcatgt

>*nlp-99(ibt13)* (2083bp deletion)

gaatgtcattctttgcgcgcttcaaccttctaagccgcgtgcacaacttttcctgtgtgcctgtgtgctgctccactagaataATGGCATCCTCCACCTCCACAACACTATTCTTCCTTCTGCTCGTTGCCACGTCATCAATGATGATGGGACACGTCTCCGCGGGCCGAATTATACCACAGgtgagaatctacaaaaaattgagcttaaattccctaaaattgagttttgagccgtgggcttggggtcttaggcttaggcttaggcttagggcaacaggtttaggtccttaggctttgggccgtaggcttagggtctcagggttagggccaggcttaagaccttagaattagggtcttaggttcaaggtcttaagcttagggtcttaggttcagggtcttaagtttaggcttaggactttagacttagggctttaggcttaaggcattaggcttaggcttaggcttaggcttaggcttaggcttaggcttaggcttaggcttaggcttaggcttaggcttaggcttaggcttaggcttaaagcctcaggcttcaggttacaatgtgactgcaaaagtctggagtttcacacaattttccgctgaaagttctaatatcccaaaattcccaaaataaacttactcccaaaatatccttgttcttcccacacacttccatggtctcatcttgaacttttttttcgccaaattttttttgccatttttatcccatacccagcttaacctcccatctattctctcattttcttgcctaaatatattttttgccccattcccccatattttttcttacccaaatccctgtcaaaagccgaaagctgtgcacacacaaactaataaagacgttgaaaaaattgaaaaaatgtgaacctaaattgggcaataaaacatcacagaggttttatgacgtaatttcgataggcttttggtggtgctgtcatttgttcgaaaattggcaaaatcgtagagcaaagggtaggggaaattagaaatatacgacatga-

agatggccgtttttgaaaatttaaaatgtcccggaaaaatgtccgaaaaatttaaaaatattgctgacgcttttttgagcaattataaaagtggtttcaccaggtcaattttcaatttttttttgtttctctcgattccaaaaatattgacctgtatggtcatctaatcggggtatcatgattttgctcgtggagcccgtgggagccggaaaagtaaaggtactt

*>nlp-99(syb4792[nlp-99::SL2(gpd-2)::mKate2])*

atgagccaaatcagttcctaactgggttaccattagttttatcgaatttttttaaaattaacataacatttgttacaaggttttctataattctgtagttgaaacttatttaatatctataaaattaatcgagatataagcttaaaaaaataatataaaaattgataatagccatttaaagccaaattttcccgccaaaaatcttcaagcttcatataattttaataaaatttctagTCCTACACCGGCGTCGACCCCGGAATCCAATTCGAGCTGCTCACCGCAAAACGTGGCGACGGATACGGATGGAACGACTGCGAGTTCTCACCACTC**AGtTGt**CTGCTCAAACGACGACGACGATCAGCTTAAgctgtctcatcctactttcacctagttaactgcttgtcttaaaatctatgcttctctttagtatctaaaattttcctagaagcttacaagtatataaatggtctcttctcaataaaggttgtatatttattcatcttattgaatctgccatttcctcgtttttgcgagtttatataccttccaattttctttctattgtattttcaacttctaattttaattcagggaaactgcttcaacgcatcATGTCCGAGCTCATCAAGGAGAACATGCACATGAAGCTCTACATGGAGGGAACCGTCAACAACCACCACTTCAAGTGCACCTCCGAGGGAGAGGGAAAGCCATACGAGGGAACCCAAACCATGCGTATCAAGgtaagtttaaacatatatatactaactaaccctgattatttaaattttcagGCCGTCGAGGGAGGACCACTCCCATTCGCCTTCGACATCCTCGCCACCTCCTTCATGTACGGATCCAAGACCTTCATCAACCACACCCAAGGAATCCCAGACTTCTTCAAGCAATCCTTCCCAGAGGGATTCACCTGGGAGCGTGTCACCACCTACGAGGACGGAGGAGTCCTCACCGCCACCCAAGACACCTCCCTCCAAGACGGATGCCTCATCTACAACGTCAAGATCCGTGGAGTCAACTTCCCATCCAACGGACCAGTCATGCAAAAGAAGACCCTCGGATGGGAGGCCTCCACCGAGACCCTCTACCCAGCCGACGGAGGACTCGAGGGACGTGCCGACATGGCCCTCAAGCTCGTCGGAGGAGGACACCTCATCTGCAACCTCAAGgtaagtttaaacatgattttactaactaactaatctgatttaaattttcagACCACCTACCGTTCCAAGAAGCCAGCCAAGAACCTCAAGATGCCAGGAGTCTACTACGTCGACCGTCGTCTCGAGCGTATCAAGGAGGCCGACAAGGAGACCTACGTCGAGCAACACGAGGTCGCCGTCGCCCGTTACTGCGACCTCCCATCCAAGCTCGGACACCGTTAAtcgacacaagcaccacatattggatcagccagttaacttttttcggaaaaaatgaaatttattttctgaaaattagagtcccctttaacatcaattgtttatttttccattcaaaaagttttttccaccaacaaaaatttggttcgtcaccactcaagcaagcaatcactattttttctatccttcccagtttccagacaccactcccctccccttcttttcgtaattattatcatcatcatcgccggcgagcgtttacttgtcattttcttctacacaagatctttttttttctttattacattatcgatatctatgaacggaacaaaagagaaaaagttgatgattttgaaataaatcatgtctacatgtttttcgttttgcagtttttcaatttttgaaacaggtatatatattgctgaaacagtttgttcttcagcaaaactgtgaactgacaa

*>nlp-99(syb4879[nlp-99::linker(GSGSG)::mKate2])*

agctccaaatcggagagattccgggtcgatgcaccatgagccaaatcagttcctaactgggttaccattagttttatcgaatttttttaaaattaacataacatttgttacaaggttttctataattctgtagttgaaacttatttaatatctataaaattaatcgagatataagcttaaaaaaataatataaaaattgataatagccatttaaagccaaattttcccgccaaaaatcttcaagcttcatataattttaataaaatttctagTCCTACACCGGCGTCGACCCCGGAATCCAATTCGAGCTGCTCACCGCAAAACGTGGCGACGGATACGGATGGAACGACTGCGAGTTCTCACCACTC**AGtTGt**CTGCTCAAACGACGACGACGATCAGCTGGATCCGGATCCGGAATGTCCGAGCTCATCAAGGAGAACATGCACATGAAGCTCTACATGGAGGGAACCGTCAACAACCACCACTTCAAGTGCACCTCCGAGGGAGAGGGAAAGCCATACGAGGGAACCCAAACCATGCGTATCAAGgtaagtttaaacatatatatactaactaaccctgattatttaaattttcagGCCGTCGAGGGAGGACCACTCCCATTCGCCTTCGACATCCTCGCCACCTCCTTCATGTACGGATCCAAGACCTTCATCAACCACACCCAAGGAATCCCAGACTTCTTCAAGCAATCCTTCCCAGAGGGATTCACCTGGGAGCGTGTCACCACCTACGAGGACGGAGGAGTCCTCACCGCCACCCAAGACACCTCCCTCCAAGACGGATGCCTCATCTACAACGTCAAGATCCGTGGAGTCAACTTCCCATCCAACGGACCAGTCATGCAAAAGAAGACCCTCGGATGGGAGGCCTCCACCGAGACCCTCTACCCAGCCGACGGAGGACTCGAGGGACGTGCCGACATGGCCCTCAAGCTCGTCGGAGGAGGACACCTCATCTGCAACCTCAAGgtaagtttaaacatgattttactaactaactaatctgatttaaattttcagACCACCTACCGTTCCAAGAAGCCAGCCAAGAACCTCAAGATGCCAGGAGTCTACTACGTCGACCGTCGTCTCGAGCGTATCAAGGAGGCCGACAAGGAGACCTACGTCGAGCAACACGAGGTCGCCGTCGCCCGTTACTGCGACCTCCCATCCAAGCTCGGACACCGTTAAtcgacacaagcaccacatattggatcagccagttaacttttttcggaaaaaatgaaatttattttctgaaaattagagtcccctttaacatcaattgtttatttttccattcaaaaagttttttccaccaacaaaaatttggttcgtcaccactcaagcaagcaatcactattttttctatccttcccagtttccagacaccactcccctccccttcttttcgtaattattatcatcatcatcgccggcgagcgtttacttgtcattttcttctacacaagatctttttttttctttattacattatcgatatctatgaacggaacaaaagag

*>flp-11(syb6321[flp-11::linker(GSGSGSGSG)::sfGFP])*

CTCCTTATTGTCTTCGTTGCCGCTTCTTTTGCTCAATCTTATGATGACGTCAGgtatagttttttcttaaaacaatttttatcaattacccatataaatctattgtagTGCGGAGAAACGTGCCATGCGGAACGCCTTGGTTCGATTTGGAAGAGCTAGTGGTGGAATGAGAAATGCTCTCGTTAGATTCGGAAAGAGGTCTCCATTGGACGAGGAAGACTTTGCTCCAGAGAGCCCACTCCAGGGAAAACGGAACGGTGCCCCACAACCATTTGgtaagttgtcttaaaatttttcttccgctttttgcctttgcttcatgtgtcgtttattttgctttgcagTTCGCTTTGGCCGATCCGGTCAACTCGACCACATGCACGACCTTTTGTCGACTCTT**CAA**AAG**CTT**AAGTTC**GCT**AACAACAAGGGATCCGGATCCGGATCCGGATCCGGAATGTCCAAGGGAGAGGAGCTCTTCACCGGAGTCGTCCCAATCCTCGTCGAGCTCGACGGAGACGTCAACGGACACAAGTTCTCCGTCCGTGGAGAGGGAGAGGGAGACGCCACCAACGGAAAGCTCACCCTCAAGTTCATCTGCACCACCGGAAAGCTCCCAGTCCCATGGCCAACCCTCGTCACCACCCTCACCTACGGAGTCCAATGCTTCTCCCGTTACCCAGACCACATGAAGCGTCACGACTTCTTCAAGTCCGCCATGCCAGAGGGATACGTCCAAGAGCGTACCATCTCCTTCAAGGACGACGGAACCTACAAGACCCGTGCCGAGGTCAAGTTCGAGGGAGACACCCTCGTCAACCGTATCGAGCTCAAGGGAATCGACTTCAAGGAGGACGGAAACATCCTCGGACACAAGCTCGAGTACAACTTCAACTCCCACAACGTCTACATCACCGCCGACAAGCAAAAGAACGGAATCAAGGCCAACTTCAAGATCCGTCACAACGTCGAGGACGGATCCGTCCAACTCGCCGACCACTACCAACAAAACACCCCAATCGGAGACGGACCAGTCCTCCTCCCAGACAACCACTACCTCTCCACCCAATCCGTCCTCTCCAAGGACCCAAACGAGAAGCGTGACCACATGGTCCTCCTCGAGTTCGTCACCGCCGCCGGAATCACCCACGGAATGGACGAGCTCTACAAGGCCGCCAACGACGAGAACTACGCCCTCGCCGCCTAAtgaccgaggacgaccgtcttctgctcgaacaactcctgaggcgaattcatcattaaaaatcatatgtttttctctctcacactctcttttttcatactctctcttgctgtctagaatttgattggtgtcgcttaacccccctttccctccgaaggaaagttatctccccagatctcttttggtgttttttatcagctaacaacacacattttctgatatttctatgctctgtctatgaacaataaaggcgttgttaattactcgcaaaatcactttgtttatttttttcacattttcagatagtgaacaaaagaaaattaaattctaaaatctgaatcggaaaattcaaattaaaaattaaatttattttttttatattacacctgttttttttcaaatattagatcaaaaactattcaacaagtggcatgtaaagcatagg

>*lgc-38(syb2346goe13[flp-11p::XCaMP-R::flp-11 3’UTR])* *III:7007600*

ACGTTGTAGTTGTAACAAGTTGCTTGTCTAATTGTAATGTTTCAAGCATCAAACGTCTTAAAGTTACTGTGGATATTGTTGTTAATTGATGATCTTTTCGTTCTTTTGGCGGCTTGATGTCTGCGAAATGTGAAGAGAATGCAGCTGCAACTACAGAAGCTACATATTGGGTTCCTGTTGGTGTCACTCGAGCAAGTACTCCTGATCCTTTCTGAAATACACTTCGATATATTTTAGCAACTTATTTTTTAAATATTAGCTCTAAAAGACAACTCCTAAACACCGTTTGAGCTTGCAGTTGTTTAATAAAAATTTAAGCTCACCAAAGTTCGATCATTGAATAGAAGTGGATTCATGTCTACAAGATCTTGAGTTGCCACCTGGCATGATGCCAACGTGGCACATAATGATATCTGAAATCAAACGTTGAAAGAGTGATCTCTTGAACACAAATGGAATTTTGTTCAATTGTCAAACACATCTTACCAGTAAAAATTTTCGCATATTGAAGTAGAATCAAATGAAAAAAAAACGGAAAATTTAAGCGAGATCAGTGTTTTGTTCTTGCCAAGGTCAGTAGAGGACAATCAGATGTTTCGACACGGGAGACCACTTGAAGGTGTAATAGATGGTTTGAGTGAAAGGGTGAGAAGCCGACTGCAAACGATATTTTTGACAATATTATAGTCACATGCAAGTTTTAATTTTAACCGAGTGAAAATATAGATATGCCAGCAGTGAAATAAATCTCTTGCTAGATTATATTTTGTTCTTTAGGTTTTTAAACTTTCCGGTGGCAAGGAACATGGGGTAGTGAAAAGAGGGGAGATTTTCAAAAACATAATAAAACTTCCAAAAGATATTAAAATTAACCCGTTTTTACAGCTTAGTGGTCCTTTAAACTAATTTTAAAGTCCGATTGACAGTGGAAAAATGTCAAAAATATAGTTAAAATAATCTCTAGGAGTGCACTTTTTCAAGAATGGCAAAGTGAAAATGAAACTAATTGTAAATGAAAAACACAATAATAAATGAATCTAGATTATTATCTTTAGAATCTAAAAAAGTTAAAACAAACCTCTAGGTCAACGTATGACTTGGAATTGAATTGAAAAACAAAGAGAAAAGAGGTGGAGAATAAAGTAAAATTAATTTACTTTTTCTACTCATTGTTTGTTGAGTAAAAGCTGTTACTCGTCAAAGAGAATCAAAAGTTGTTTGTCTTTAACGTGGTCAATAATTTTATGATGACCTCAATATTGTTCTCGAGGTGATTCTAGAAGAAAGGGCTTGAAGCTGATTATTTTGAATACCTAAAACTTCAAAGATCTTTTCAAAACATTTAGTAACAAATAATAAACCGAAACAAAAAAGAAATTATCATTGGTTTGCCAATGAAACAGATATGCAAAGATGAATGGCAATAAAACTGGACATCAGGTCACTTTTGGGGCATGATCAATGTGATTGACTACTTTGTGTACTTTACAAAATGTCACTATTTGCAGGAAAAAATCAACTAAAAAGAAAGCTTCTAACTTCGACTTAAGACCCTTTCGACTTAATCACATATTTCTAAACTTCACGAGAATCAGGAGTCAGAAGAAGGACAGTGGATATTCAGAATAAACTTCAATCTTAAGAAATTGAAAATAAATTACTGGAAAATATTGGAACACTTGAAATGTTGCAGTTCCAAAGTTAAATGGACACTGCAAAAGACCTGCACTGACCACTAGATACCAATTATATAGTCTCATTCAAAACTGAAACATTTCTCTCCGTCTCCACATTCTCCCATACTATTTCTTGATTTTCGAACAAAGAAACGACCTACAAGAGGTAAATGGCTACTCTTCTATTGTTTCAGTCTTTCATTACAACACAAGGAACGAGAAGCTTGTGGCATTGACTGTAAGAGAGTTGGACAAAAAAAGAGAGTATAAGACAACGAGAGTGACCATAGATGGACCTGATGAAAGTGATTTTTATCATCTCTGAAAAACTCGGGTAAGGGACAACAATTGGTTGGGTAAGTAGAAGTAAAAAGAACTGAAAACACACTCCCCTTTGTGTTTTCTATTGTCTTTTCTTATATTATTCTTCTTTTTGATCTTTTTTTTAATGAAACAGTCTTCTGATTCTTTTTGTTGATATGAAGACAGAATGGAGGGGACACTTGTTTGTTACAATATGCTTACTGATCATATCCCAATATATATGACGTTCTTTTCAAAATTTGATTGTTTTTGATTTCAAAAATGACAACAAAGAAACTAGATTTATAGGTACTCGGTTGAAAATTTGCAAAAATTAGTGTGTAGTTTCAACTTCTACAATAGTTCTTGTACAGTAAAGATCAAAACGTAGCCGTTGGGAAGCGTATAGATATACTCGACACTGTAACTTGAAAAACTGCAGAAGTTAAAAAATGAATTTGCACAGGCTGCGCTAGCCTCTCCAGTTTTATTGGTATTTCAAATCATTTTCTCGTCTACTTGGTGCAACACGCGTGCCTCTGTAGCAAAGACTGAACTACTTCGCATACTTCCATGTATTTAACACGTCGTCTTGTAAAAATAGTTTGAGAGATTTTTTCACCATTCTCGTTTTCCGTATTCATTCAACAACCGATGAAAAATCCGTATTTTTCCCGAATAAATATTCAGTGATTGCGAGAAGAAATTATTTTAATGATCAAACTCTAATGATTCTTGATAAAGAATGTATTGTTTTGTAAAATAAAGAAAACAAGTATTCTAGGTAACATATTAACCTGGGAACAATAAGTCGGTGAAGTGATGTCATTGAAAATACATAATGGCGATGTCATTTTCATGTTATTATAGTTAAAGACTGTGATTTTTAATTATAAATTGTAATTAATTATTGATGTTCTAGTTAAGTTTGGTTGATTGAAGAAATTGTATTTTTTAACTTTTGTAAGGGATATGACTTTTTTGAGTTAAATAAATGAGGTATATAGTTAAATAATTTGTTTTTTTGAAGGATTTTTGTGCAGCGTTGTTTTAAAAAACGGTTTTCATAAGATTTTTATCACAGTTGAACTCCTATATTTCTTCCCTCACTAATTCTTGATGTGTGGACAAAACTGTAAGTCTGATCAAGGCAACAACACAACATAGACCACTTTTCTAGTCCAGTAAAAAATCGAACTCTGATATTCAGACTGGTATCACTTCTTAGAGTCAGAAGATGGTACAGCTTGGGAAAAACTTGGAAAAACTATCCCAGACTGCCAAAACTGAAGCTTAAAGTGAGCACAAAATTGCTCCAAACTTTTTTTGCAGCTCACATTCGGTCCAAGTGTTGTTCAATTTATGATGATTATCAGTCTGTTTTGCCAAATTCGGATCCAACTTTTTTTTTTTTTTTAGGAGAATTTATTTTAAAGCTCGCCATTGAAATACATGTAAAAATCTAAGCTTTTAAATTTAACAGAAAATGAGATTATCTTTTTTGGAATGAAACCTAGAATTTTCAAAATAACTAGCATCTCAAAATAACTAGCATCCAAGATGCTCTTTGTTTTCATAAATTTTAGTTGTTTCCAGTCCCGAAAAAAAATTTCAATCATTTCCTTAGGTTCAATAAGTGTTTTGTCTGGTCTCTTGTTGCAATTTTTGAAAAAGGAATAACATTGTGCTCAGACTGAAAAATGAAAAAAGATGATGTAAGTTGGTCTAGGCTTTAATTCCAGTTTCCGTTCCACTAGTGATCTTATCATTTATTCATATCAAGTTTCATTTGATGTTCGATTCATTGTAGGTTTCATTGTTCCCTCTGACTATTCATCAAGTTCTATGAAACCATTGTGGCTTACTAGTTCCCCAAAGTTGGAAAGATCTGGAGAAAGAAAAACAAGTATCCCAAAAGCCCAAACCTTTTTTTTCCAAATGTTGAGCCTCTAAAGTTGATGAATGGGGTGAGAGAGGTTTTCATGGTTTTATGGCCCATCAGTTCCAGTAGAGTTCCTCATATCTTTTCGCACTTCATTTTTCAATCTGTTAAGAGTAATTCCCCCACAACTTTTAGCATCCTCAATCCTGGTTCCACCCACAAACACCCACACTCCATTGAATATAAGATTATAAAATATAAGTTAAAATACGAAAAGATGCAAAACTGTGACGACGAAACTTAACAACCTGCTGACGTGTCAAATATTTCCGCATCTTACCTTCGTGGGCATCTTCTAACTCATAAATTTATGATGTTCGAATCCCGGCGCCAAGTGATGTAAACTCATTTAATATTTGAGGAAAAAGCATAGACGAATCCATTTTGCATCTAATTTATTTTTCTAGTTTCACACCCATGTTTCGCTTGGCATAATCAGTGTTTGAGCGCACTTTTTGCAAGGTGCGTCTTCTTGTAAAGAAAGATTTTCAAAAAAGTGCAAAAGTGGGGGAGATTGAAAAACGATAAGAAGGTTTCATGTCAAACAAGGACACACGAATATATAAATACGGGTCATTTTTCAAACTTTCCATTGTGACTTTTTGGAATATTTTTTTCGTAAAAATGAAATCGATTTTCTGACCAATATCTCAACTTTAAAACTTCTAATTCTGCTTTTTTTCAATCTTCTGTAAACAGGAGTGCGTCGAAATTTCGGCAGTTTCGGCATATTGCCAAAAGTTTATAGTCCAACAATTGCCGGAACTGGTGATTTAGCTAAACAATAAACCTCAAAATATTAGGTATTTCAGTAAGAAAATGACCTATTGATTATGACTTTGAATCAAGTTTTGATATATTTTTAATTTTTGAAATATGATGAAAATTAAAAATTATCAAAAATTCTGGCAATTTTGGCACATCGGCACACAATGCGAAATTTGCGGCAACTGCCGATTTTTAAAAAACTTTATTTGTTTGGTATAACAGTTAGGCACGCATTTTGGAAATAAACCGATCTTTTATTGGGAGAAGTGCCAGTTAAAGTGCTGGACCAAGTAAAATGTTTCCAAAAAACCAGACACGTATTAATAAAACACTTCAAACTTTTTCAAATTTTACAACAATTTTCAATTAAAGTTTTGGCAAATTGGTAAATTTTCAAGAAATTTTTTTGCAAGTTTTAATTTTTAAATATACATTATAATTATTAGAATGTTTTCTGTATAGAAAATCTCTGCATCTGTTGGAAATGAATAAAAACTTCATAACAATTCTAAATTAAAAATTCAAGAACACTAGAACAAGCGTCCTCAAAATATTCACAAAATATTGTACACATTTTGTAGTACTTTTTCCATTATTAAAAAAAGCAAAACTAGCTTTTCCTTCCTTTCCGAAATTTAATGCTATTTTCAAGATGACTTTTTTGCTTGCGTTTTTCTCAGTTTCCTCACACACACACACACACAAGTAGGCGTGGCCTGTGGAACGTTTCAGAGCGCAGAACACCTGCATTTGATCTATTCACTTCTTGCTTTTGAAAAGCCCAAAGACACCCTACACTTCGGTTTCGTTTTGGAAACCATTGACATCATCCTATTTTCCATAAGAAGTTTCCTTGAGAAGAATCCATTTCGCAAATTTTTCATTAAAACGTTCAAAACTCATCAAACCATTTGTAAATAGTAATAAAGTATGTCCTGCGGCTATTTGCTTTCTCTTCGGAATCTACAACGCCCCCTCCTAATACATCGTTTCAGGTATAAAAAGACTGCGCCTAGCCGCTCGTCTCACTTTTTGCAGTTCATACTGAATA*aaaaa*ATGGGATCCCACCACCACCACCACCACGGAATGGCCTCCATGACCGGAGGACAACAAATGGGACGTGACCTCTACGACGACGACGACAAGGACCTCGCCACCATGGTCGACGTCAAGCTCATCCCATCCCTCGCCACCGTCATCCTCGTCAAGTCCATGCTCCGTAAGCGTTCCTTCGGAAACCCATTCCCAGTCGTCTCCGAGCGTATGTACCCAGAGGACGGAGCCCTCAAGTCCGAGATCAAGAAGGGACTCCGTCTCAAGGACGGAGGACACTACGCCGCCGAGGTCAAGACCACCTACAAGGCCAAGgtaagtttaaacatatatatactaactaaccctgattatttaaattttcagAAGCCAGTCCAACTCCCAGGAGCCTACATCGTCGACATCAAGCTCGACATCGTCTCCCACAACGAGGACTACACCATCGTCGAGCAATGCGAGCGTGCCGAGGGACGTCACTCCACCGGAGGAATGGACGAGCTCTACAAGGGAGGAACCGGAGGATCCCTCGTCTCCAAGGGAGAGGAGGACAACATGGCCATCATCAAGGAGTTCATGCGTTTCAAGGTCCACATGGAGGGATCCGTCAACGGACACGAGTTCGAGATCGAGGGAGAGGGAGAGGGACGTCCATACGAGGCCTTCCAAACCGCCAAGCTCGTCGTCGTCAAGGGAGGACCACTCCCATTCGCCTGGGACATCCTCTCCCCACAATTCATGTACGGATCCAAGgtaagtttaaacagttcggtactaactaaccatacatatttaaattttcagGCCTACATCAAGCACCCAGCCGACATCCCAGACTACTTCAAGCTCTCCTTCCCAGAGGGATTCCGTTGGGAGCGTGTCATGAACTTCGAGGACGGAGGAATCATCCACGTCAACCAAGACTCCTCCCTCCAAGACGGAGTCTTCATCTACAAGGTCAAGCTCCGTGGAACCAACTTCCCACCAGACGGACCAGTCATGCAAAAGAAGACCATGGGATGGGAGGCCACCCGTGACCAACTCACCGAGGAGCAAATCGCCGAGTTCAAGGAGGCCTTCTCCCTCTTCGACAAGGACGGAGACGGAACCATCACCACCAAGGAGCTCGGAACCGTCATGCGTTCCCTCGGACAAAACCCAACCGAGGCCGAGCTCCAAGACATGATCAACGAGGTCGACGCCGACGGAGACGGAACCTTCGACTTCCCAGAGTTCCTCACCATGATGGCCCGTAAGgtaagtttaaacatgattttactaactaactaatctgatttaaattttcagATGTCCTACCGTGACACCGAGGAGGAGATCCGTGAGGCCTTCCGTGTCTTCGACAAGGACGGAAACGGATACATCGGAGCCGCCGAGCTCCGTCACGTCATGACCGACCTCGGAGAGAAGCTCACCGACGAGGAGGTCGACGAGATGATCCGTGTCGCCGACATCGACGGAGACGGACAAGTCAACTACGAGGAGTTCGTCCAAATGATGACCGCCAAGGGAGGAGGAACCGGAGGATCCGGAGGAGGAGGAGGAGGAGAGTTCCCAGTCAAGCAAACCCTCAACTTCGACCTCCTCAAGCTCGCCGGAGACGTCGAGTCCAACCCATAAAAATCATATGTTTTTCTCTCTCACACTCTCTTTTTTCATACTCTCTCTTGCTGTCTAGAATTTGATTGGTGTCGCTTAACCCCCCTTTCCCTCCGAAGGAAAGTTATCTCCCCAGATCTCTTTTGGTGTTTTTTATCAGCTAACAACACACATTTTCTGATATTTCTATGCTCTGTCTATGAACAATAAAGGCGTTGTTAATTACTCGCAAAATCACTTTGTTTATTTTTTTCACATTTTCAGATAGTGAACAAAAGAAAATTAAATTCTAAAATCTGAATCGGAAAATTCAAATTAAAAATTAAATTTATTTTTTTTATATTACACCTGTTTTTTTTCAAATATTAGATCAAAAAACGAGGTATATGGGCTTCAGATCTTCAACTGGAGAGCATCATTAAAATCAGATAACCGTCAAACAACTGGGATAGAAGTGTTTCCTTCTGAAAGTTTACCAACTTTTCAAAAATTAATCCGAAATTATTCTACCATCCAATTCGTTTGATCTTGATTTTGATTGGCAAATTGTAAAATAAATTATAAAATTATGATTTCTCCCACTTTGGACCGCCAGACATTTTTTATATTTCCACCCACTTTTTCTTGAAACGTCATATCGTGTTAGTCGAACATCCATTTTCTTTCTTGAAAGATATATCATGAAATTCAGTGATAGAAGCAAAAAAAAAACGAAAAAAAAGGAACATGCATTTCAGAAGACACTTCCTTATGAGGATCATTTATAACAGAATAACTTTGGCCACAAATATATGAATTTGCAGCCGCTTTCTCGGAAATTGCCAAATTTCAACAGTTTTCTTAACTCGAATGTTCCATGTTATTCTGAAAGTTTGGAAAGTGATCAATTTGAAAGTGTAACAGAAAATTATTCAAAAAGAAGTTCTGAATATTCGCTTATATTGCAACTGCAGCACAATATAAATATAAACTTGACAGGTTATTCTCTCTGATGACTTGTTGATCTTGTGGGACGTGCGAAACTGACACTGAATTTAGAAAACTACCTTTTTGTTTGAACAATTGGCATATTGTTCGTGTTTTGTGAAGCAGAACTGCCGTTCTATTTCGGATTCTTGAAAACTAGACTACGTTCTAGGATAATTTTTCGTCTTGAGAATATGAGTTTCTGAAGACTGATACTTTGGTTTGTTATGTTTTATGGTCACATAGCCAAATTTTGTTTGGAAAAGAATCAGAATCTAATAATTTTCTTTTATTGAAAACTTTGTTTCTTGGGGGATTTTTCCGATACATTTTCAAAATTGAATTGATTTTTCATCAATTTTTTAAATTTTCCAATACAAACTTTTTACAATTGGAGTGATGTTGGATGTACAAAAATTCAGTGACGTCCTACTATATAAGAAACAAAGGTCATTCTCTTACATTGTTTTTCTTGCACTGACTTTTCACCTTTATTGTCTTTTCTTTTCTTCTCAACCATCATGCTCCTGCAGAGATAATAACCCTTTTTTTGCTTCCAACAACTTCCAGAATTTCGAAAAATTTACACGCGGAATTCAGAAGAGGACGTCATACTCGTGTCTTCAACTTCTTCTACCGCCACCATCGTCGGACACAGAAACACACAAGACCGCCCTCTCCTCTCATGTTTTCCGGTTGAAAAAAGAAGGAGCAGAAAAGAAATGGGCGGAGCTTCGGACGGGTGCTCTCGGTGGCGCGGAGCACATGCACACACATACGCTTCGACCCCTTCTTCTGGCTCCCGAGGAGCCCAACACCATCACCACCATCACCATCATTGAGTGTGTCTACTCATATTGGTGACAGAAGATATGATGACGTGGCTATTATGGTCACTACTCATTGGTTATGTGACAACAGAACATCCACCGGTAGGTAGGGTACGGTATTGGAACACGATTGGAATTTGTATCACCTGTAAATTTATTTTTACGTTTGATTGGAAGTCACATTTGAAATAGGTACTCGTTTTTCCATACACATGAGATCAAGTGAAGTAGGATATATCTTTCTACAAACGTCAATTTGTTTAGAAGATGTCATGCAAGAAATAGGTTGAATTAACTAGATCTCTTGCTATAACTGAAATTATTTTAGATTGACTGACAATCTAAATTATCAGAAACAATTCACGAAATTATTAAAACATTTTCAGCTCATGAAAAGTGACAAACTTATCGAATACGCACCACAAGCGAGCACAGAACCTACTTTAGGGTAAGTACTTTCCTTTTTTGTCTGTTTTCCGAACATCTTTCCTCTTTAACAAGTCGTTATCCCCTCAGGGACACCTCTTTGTGGTGGGTCATTTATCTTATTTTAGACAAAAAACAAAACAACCTCTTTCTTATTCTTA

>*lgc-38(syb2346syb2493syb8234[flp-11-5'utr(620bp)::ReaChR-linker-mKate2::flp-11b-3’UTR] ) III:7007600.*

AGAAGTTAAAAAATGAATTTGCACAGGCTGCGCTAGCCTCTCCAGTTTTATTGGTATTTCAAATCATTTTCTCGTCTACTTGGTGCAACACGCGTGCCTCTGTAGCAAAGACTGAACTACTTCGCATACTTCCATGTATTTAACACGTCGTCTTGTAAAAATAGTTTGAGAGATTTTTTCACCATTCTCGTTTTCCGTATTCATTCAACAACCGATGAAAAATCCGTATTTTTCCCGAATAAATATTCAGTGATTGCGAGAAGAAATTATTTTAATGATCAAACTCTAATGATTCTTGATAAAGAATGTATTGTTTTGTAAAATAAAGAAAACAAGTATTCTAGGTAACATATTAACCTGGGAACAATAAGTCGGTGAAGTGATGTCATTGAAAATACATAATGGCGATGTCATTTTCATGTTATTATAGTTAAAGACTGTGATTTTTAATTATAAATTGTAATTAATTATTGATGTTCTAGTTAAGTTTGGTTGATTGAAGAAATTGTATTTTTTAACTTTTGTAAGGGATATGACTTTTTTGAGTTAAATAAATGAGGTATATAGTTAAATAACATTATAATTATTAGAATGTTTTCTGTATAGAAAATCTCTGCATCTGTTGGAAATGAATAAAAACTTCATAACAATTCTAAATTAAAAATTCAAGAACACTAGAACAAGCGTCCTCAAAATATTCACAAAATATTGTACACATTTTGTAGTACTTTTTCCATTATTAAAAAAAGCAAAACTAGCTTTTCCTTCCTTTCCGAAATTTAATGCTATTTTCAAGATGACTTTTTTGCTTGCGTTTTTCTCAGTTTCCTCACACACACACACACACAAGTAGGCGTGGCCTGTGGAACGTTTCAGAGCGCAGAACACCTGCATTTGATCTATTCACTTCTTGCTTTTGAAAAGCCCAAAGACACCCTACACTTCGGTTTCGTTTTGGAAACCATTGACATCATCCTATTTTCCATAAGAAGTTTCCTTGAGAAGAATCCATTTCGCAAATTTTTCATTAAAACGTTCAAAACTCATCAAACCATTTGTAAATAGTAATAAAGTATGTCCTGCGGCTATTTGCTTTCTCTTCGGAATCTACAACGCCCCCTCCTAATACATCGTTTCAGGTATAAAAAGACTGCGCCTAGCCGCTCGTCTCACTTTTTGCAGTTCATACTGAATA*aaaaa*ATGGTCTCCCGTCGTCCATGGCTCCTCGCCCTCGCCCTCGCCGTCGCCCTCGCCGCCGGATCCGCCGGAGCCTCCACCGGATCCGACGCCACCGTCCCAGTCGCCACCCAAGACGGACCAGACTACGTCTTCCACCGTGCCCACGAGCGTATGCTCTTCCAAACCTCCTACACCCTCGAGAACAACGGATCCGTCATCTGCATCCCAAACAACGGACAATGCTTCTGCCTCGCCTGGCTCAAGTCCAACGGAACCAACGCCGAGAAGCTCGCCGCCAACATCCTCCAATGGGTCGTCTTCGCCCTCTCCGTCGCCTGCCTCGGATGGTACGCCTACCAAGCCTGGCGTGCCACCTGCGGATGGGAGGAGGTCTACGTCGCCCTCATCGAGATGATGAAGTCCATCATCGAGGCCTTCCACGAGTTCGACTCCCCAGCCACCCTCTGGCTCTCCTCCGGAAACGGAGTCGTCTGGATGCGTTACGGAGAGTGGCTCCTCACCTGCCCAGTCATCCTCATCCACCTCTCCAACCTCACCGGACTCAAGgtaagtttaaacatatatatactaactaaccctgattatttaaattttcagGACGACTACTCCAAGCGTACCATGGGACTCCTCGTCTCCGACGTCGGATGCATCGTCTGGGGAGCCACCTCCGCCATGTGCACCGGATGGACCAAGATCCTCTTCTTCCTCATCTCCCTCTCCTACGGAATGTACACCTACTTCCACGCCGCCAAGGTCTACATCGAGGCCTTCCACACCGTCCCAAAGGGACTCTGCCGTCAACTCGTCCGTGCCATGGCCTGGCTCTTCTTCGTCTCCTGGGGAATGTTCCCAGTCCTCTTCCTCCTCGGACCAGAGGGATTCGGACACATCTCCCCATACGGATCCGCCATCGGACACTCCATCCTCGACCTCATCGCCAAGgtaagtttaaacagttcggtactaactaaccatacatatttaaattttcagAACATGTGGGGAGTCCTCGGAAACTACCTCCGTGTCAAGATCCACGAGCACATCCTCCTCTACGGAGACATCCGTAAGAAGCAAAAGATCACCATCGCCGGACAAGAGATGGAGGTCGAGACCCTCGTCGCCGAGGAGGAGGACAAGTACGAGTCCTCCGGAGGATCCGGAGGAGGATCCGGAGGAATGTCCGAGCTCATCAAGGAGAACATGCACATGAAGCTCTACATGGAGGGAACCGTCAACAACCACCACTTCAAGTGCACCTCCGAGGGAGAGGGAAAGCCATACGAGGGAACCCAAACCATGCGTATCAAGGCCGTCGAGGGAGGACCACTCCCATTCGCCTTCGACATCCTCGCCACCTCCTTCATGTACGGATCCAAGgtaagtttaaacatgattttactaactaactaatctgatttaaattttcagACCTTCATCAACCACACCCAAGGAATCCCAGACTTCTTCAAGCAATCCTTCCCAGAGGGATTCACCTGGGAGCGTGTCACCACCTACGAGGACGGAGGAGTCCTCACCGCCACCCAAGACACCTCCCTCCAAGACGGATGCCTCATCTACAACGTCAAGATCCGTGGAGTCAACTTCCCATCCAACGGACCAGTCATGCAAAAGAAGACCCTCGGATGGGAGGCCTCCACCGAGACCCTCTACCCAGCCGACGGAGGACTCGAGGGACGTGCCGACATGGCCCTCAAGCTCGTCGGAGGAGGACACCTCATCTGCAACCTCAAGACCACCTACCGTTCCAAGAAGCCAGCCAAGAACCTCAAGATGCCAGGAGTCTACTACGTCGACCGTCGTCTCGAGCGTATCAAGGAGGCCGACAAGGAGACCTACGTCGAGCAACACGAGGTCGCCGTCGCCCGTTACTGCGACCTCCCATCCAAGCTCGGACACCGTTAAAAATCATATGTTTTTCTCTCTCACACTCTCTTTTTTCATACTCTCTCTTGCTGTCTAGAATTTGATTGGTGTCGCTTAACCCCCCTTTCCCTCCGAAGGAAAGTTATCTCCCCAGATCTCTTTTGGTGTTTTTTATCAGCTAACAACACACATTTTCTGATATTTCTATGCTCTGTCTATGAACAATAAAGGCGTTGTTAATTACTCGCAAAATCACTTTGTTTATTTTTTTCACATTTTCAGATAGTGAACAAAAGAAAATTAAATTCTAAAATCTGAATCGGAAAATTCAAATTAAAAATTAAATTTATTTTTTTTATATTACACCTGTTTTTTTTCAAATATTAGATCAAAAAACGAGGTATATGGGCTTCAGATCTTCAACTGGAGAGCATCATTAAAATCAGATAACCGTCAAACAACTGGGATAGAAGTGTTTCCTTCTGAAAGTTTACCAACTTTTCAAAAATTAATCCGAAATTATTCTACCATCCAATTCGTTTGATCTTGATTTTGATTGGCAAATTGTAAAATAAATTATAAAATTATGATTTCTCCCACTTTGGACCGCCAGACATTTTTTATATTTCCACCCACTTTTTCTTGAAACGTCATATCGTGTTAGTCGAACATCCATTTTCTTTCTTGAAAGATATATCATGAAATTCAGTGATAGAAGCAAAAAAAAAACGAAAAAAAAGGAACATGCATTTCAGAAGACACTTCCTTATGAGGATCATTTATAACAGAATAACTTTGGCCACAAATATATGAATTTGCAGCCGCTTTCTCGGAAATTGCCAAATTTCAACAGTTTTCTTAACTCGAATGTTCCATGTTATTCTGAAAGTTTGGAAAGTGATCAATTTGAAAGTGTAACAGAA

>*lgc-38(syb2346syb2496syb8234syb10545[flp-11-5’utr::nlp-99(CAI1.0)::SL2(gpd-2)::mKate2::flp-11b-3’utr]) III:7007600*.

AGAAGTTAAAAAATGAATTTGCACAGGCTGCGCTAGCCTCTCCAGTTTTATTGGTATTTCAAATCATTTTCTCGTCTACTTGGTGCAACACGCGTGCCTCTGTAGCAAAGACTGAACTACTTCGCATACTTCCATGTATTTAACACGTCGTCTTGTAAAAATAGTTTGAGAGATTTTTTCACCATTCTCGTTTTCCGTATTCATTCAACAACCGATGAAAAATCCGTATTTTTCCCGAATAAATATTCAGTGATTGCGAGAAGAAATTATTTTAATGATCAAACTCTAATGATTCTTGATAAAGAATGTATTGTTTTGTAAAATAAAGAAAACAAGTATTCTAGGTAACATATTAACCTGGGAACAATAAGTCGGTGAAGTGATGTCATTGAAAATACATAATGGCGATGTCATTTTCATGTTATTATAGTTAAAGACTGTGATTTTTAATTATAAATTGTAATTAATTATTGATGTTCTAGTTAAGTTTGGTTGATTGAAGAAATTGTATTTTTTAACTTTTGTAAGGGATATGACTTTTTTGAGTTAAATAAATGAGGTATATAGTTAAATAACATTATAATTATTAGAATGTTTTCTGTATAGAAAATCTCTGCATCTGTTGGAAATGAATAAAAACTTCATAACAATTCTAAATTAAAAATTCAAGAACACTAGAACAAGCGTCCTCAAAATATTCACAAAATATTGTACACATTTTGTAGTACTTTTTCCATTATTAAAAAAAGCAAAACTAGCTTTTCCTTCCTTTCCGAAATTTAATGCTATTTTCAAGATGACTTTTTTGCTTGCGTTTTTCTCAGTTTCCTCACACACACACACACACAAGTAGGCGTGGCCTGTGGAACGTTTCAGAGCGCAGAACACCTGCATTTGATCTATTCACTTCTTGCTTTTGAAAAGCCCAAAGACACCCTACACTTCGGTTTCGTTTTGGAAACCATTGACATCATCCTATTTTCCATAAGAAGTTTCCTTGAGAAGAATCCATTTCGCAAATTTTTCATTAAAACGTTCAAAACTCATCAAACCATTTGTAAATAGTAATAAAGTATGTCCTGCGGCTATTTGCTTTCTCTTCGGAATCTACAACGCCCCCTCCTAATACATCGTTTCAGGTATAAAAAGACTGCGCCTAGCCGCTCGTCTCACTTTTTGCAGTTCATACTGAATA*aaaaa*ATGGCCTCCTCCACCTCCACCACCCTCTTCTTCCTCCTCCTCGTCGCCACCTCCTCCATGATGATGGGACACGTCTCCGCCGGACGTATCATCCCACAATCCTACACCGGAGTCGACCCAGGAATCCAATTCGAGCTCCTCACCGCCAAGCGTGGAGACGGATACGGATGGAACGACTGCGAGTTCTCCCCACTCTCCTGCCTCCTCAAGCGTCGTCGTCGTTCCGCCTAAgctgtctcatcctactttcacctagttaactgcttgtcttaaaatctatgcttctctttagtatctaaaattttcctagaagcttacaagtatataaatggtctcttctcaataaaggttgtatatttattcatcttattgaatctgccatttcctcgtttttgcgagtttatataccttccaattttctttctattgtattttcaacttctaattttaattcagggaaactgcttcaacgcatcATGTCCGAGCTCATCAAGGAGAACATGCACATGAAGCTCTACATGGAGGGAACCGTCAACAACCACCACTTCAAGTGCACCTCCGAGGGAGAGGGAAAGCCATACGAGGGAACCCAAACCATGCGTATCAAGGCCGTCGAGGGAGGACCACTCCCATTCGCCTTCGACATCCTCGCCACCTCCTTCATGTACGGATCCAAGgtaagtttaaacatgattttactaactaactaatctgatttaaattttcagACCTTCATCAACCACACCCAAGGAATCCCAGACTTCTTCAAGCAATCCTTCCCAGAGGGATTCACCTGGGAGCGTGTCACCACCTACGAGGACGGAGGAGTCCTCACCGCCACCCAAGACACCTCCCTCCAAGACGGATGCCTCATCTACAACGTCAAGATCCGTGGAGTCAACTTCCCATCCAACGGACCAGTCATGCAAAAGAAGACCCTCGGATGGGAGGCCTCCACCGAGACCCTCTACCCAGCCGACGGAGGACTCGAGGGACGTGCCGACATGGCCCTCAAGCTCGTCGGAGGAGGACACCTCATCTGCAACCTCAAGACCACCTACCGTTCCAAGAAGCCAGCCAAGAACCTCAAGATGCCAGGAGTCTACTACGTCGACCGTCGTCTCGAGCGTATCAAGGAGGCCGACAAGGAGACCTACGTCGAGCAACACGAGGTCGCCGTCGCCCGTTACTGCGACCTCCCATCCAAGCTCGGACACCGTTAAAAATCATATGTTTTTCTCTCTCACACTCTCTTTTTTCATACTCTCTCTTGCTGTCTAGAATTTGATTGGTGTCGCTTAACCCCCCTTTCCCTCCGAAGGAAAGTTATCTCCCCAGATCTCTTTTGGTGTTTTTTATCAGCTAACAACACACATTTTCTGATATTTCTATGCTCTGTCTATGAACAATAAAGGCGTTGTTAATTACTCGCAAAATCACTTTGTTTATTTTTTTCACATTTTCAGATAGTGAACAAAAGAAAATTAAATTCTAAAATCTGAATCGGAAAATTCAAATTAAAAATTAAATTTATTTTTTTTATATTACACCTGTTTTTTTTCAAATATTAGATCAAAAAACGAGGTATATGGGCTTCAGAT*C*TTCAACTGGAGAGCATCATTAAAATCAGATAACCGTCAAACAACTGGGATAGAAGTGTTTCCTTCTGAAAGTTTACCAACTTTTCAAAAATTAATCCGAAATTATTCTACCATCCAATTCGTTTGATCTTGATTTTGATTGGCAAATTGTAAAATAAATTATAAAATTATGATTTCTCCCACTTTGGACCGCCAGACATTTTTTATATTTCCACCCACTTTTTCTTGAAACGTCATATCGTGTTAGTCGAACATCCATTTTCTTTCTTGAAAGATATATCATGAAATTCAGTGATAGAAGCAAAAAAAAAACGAAAAAAAAGGAACATGCATTTCAGAAGACACTTCCTTATGAGGATCATTTATAACAGAATAACTTTGGCCACAAATATATGAATTTGCAGCCGCTTTCTCGGAAATTGCCAAATTTCAACAGTTTTCTTAACTCGAATGTTCCATGTTATTCTGAAAGTTTGGAAAGTGATCAATTTGAAAGTGTAACAGAA
